# Transcriptional Landscape of Vero E6 Cells during Early Swine Acute Diarrhea Syndrome Coronavirus Infection

**DOI:** 10.3390/v13040674

**Published:** 2021-04-14

**Authors:** Siying Zeng, Ouyang Peng, Ruipu Sun, Qiuping Xu, Fangyu Hu, Yan Zhao, Chunyi Xue, Yongchang Cao, Hao Zhang

**Affiliations:** 1 State Key Laboratory of Biocontrol, Life Sciences School, Sun Yat-sen University, Guangzhou 510006, China; zengsy8@mail2.sysu.edu.cn (S.Z.); pengouy@mail2.sysu.edu.cn (O.P.); hufy5@mail2.sysu.edu.cn (F.H.); zhaoy288@mail2.sysu.edu.cn (Y.Z.); xuechy@mail.sysu.edu.cn (C.X.); caoych@mail.sysu.edu.cn (Y.C.); 2 Guangdong Provincial Key Laboratory of Malignant Tumor Epigenetics and Gene Regulation, Sun Yat-sen University, Guangzhou 510120, China; sunrp3@mail2.sysu.edu.cn (R.S.); xqp1011@126.com (Q.X.)

**Keywords:** coronavirus, SADS-CoV, transcriptome, autophagy

## Abstract

Swine acute diarrhea syndrome coronavirus (SADS-CoV) is a newly emerged and highly pathogenic virus that is associated with fatal diarrhea disease in piglets, causing significant economic losses to the pig industry. At present, the research on the pathogenicity and molecular mechanisms of host-virus interactions of SADS-CoV are limited and remain poorly understood. Here, we investigated the global gene expression profiles of SADS-CoV-infected Vero E6 cells at 12, 18, and 24 h post-infection (hpi) using the RNA-sequencing. As a result, a total of 3324 differentially expressed genes (DEG) were identified, most of which showed a down-regulated expression pattern. Functional enrichment analyses indicated that the DEGs are mainly involved in signal transduction, cellular transcription, immune and inflammatory response, and autophagy. Collectively, our results provide insights into the changes in the cellular transcriptome during early infection of SADS-CoV and may provide information for further study of molecular mechanisms.

## 1. Introduction

Coronaviruses (CoVs) are enveloped, single-stranded, positive-sense RNA viruses that have a broad range of infection species, such as humans, bats, camels, and pigs [1,2,3,4]. CoVs belong to the family *Coronaviridae* and are classified into four genera: Alphacoronavirus, Betacoronavirus, Gammacoronavirus, and Deltacoronavirus [5,6]. Most importantly, the recent pandemic of Coronavirus Disease 2019, caused by SARS-CoV2, infected ~83 million people with more than 1.8 million deaths around the globe [7,8,9]. In addition, a bat HKU2-CoV-like novel porcine CoV, also called swine acute diarrhea syndrome (SADS)-CoV, is associated with diarrhea in pigs [10,11]. The replication of SADS-CoV in multiple cells indicated its potential to cross the species barrier [12], and thus it needs to be paid great attention.

According to the latest International Committee on Taxonomy of Viruses (ICTV) Master Species List (https://talk.ictvonline.org/taxonomy/, accessed on 6 April 2021), swine acute diarrhea syndrome coronavirus (SADS-CoV) belongs to the *Coronaviridae* family, *Orthocoronavirinae* subfamily, *Alphacoronavirus* genus [1,2,3]. First discovered from swine in southern China in 2017, SADS-CoV is the sixth porcine CoV identified in pigs, with others including porcine epidemic diarrhea virus (PEDV), transmissible gastroenteritis virus (TGEV), porcine respiratory coronavirus (PRCV), porcine hemagglutinating encephalomyelitis virus (PHEV) and porcine deltacoronavirus (PDCoV) [4]. Clinical signs of SADS include acute vomiting and severe acute diarrhea, which are similar to PEDV and PDCoV infection [4]. The morbidity and mortality are high in piglets, but mortality is low in older pigs [3]. In addition, several recent studies revealed a remarkably broad spectrum of SADS-CoV cell tropism, including cells from pigs, rats, monkeys, and even humans [13,14,15]. Given the frequent contact between bats, pigs, and humans, we should be alert to the possibility of cross-species transmission of SADS-CoV and emphasize the need to understand molecular mechanisms of host-virus interactions.

Transcriptome sequencing is widely used in gene expression level analysis and differential expression analysis, new gene mining, single nucleotide polymorphism search, and gene function annotation [7,8,9,10]. In recent years, the application of high-throughput RNA sequencing (RNA-Seq) technology in the field of viral infection and disease has increased in frequency. It is an effective research tool in revealing gene interaction networks to understand the host-pathogen relationship and develop new strategies that can be used for therapeutic and preventive interventions [11]. Recently, Zhang et al. [12] investigated the whole transcriptome profiles of intestinal porcine epithelial cells (IPEC-J2) at 6, 24, and 48 h post-infection (hpi) of SADS-CoV. The results provided new insights into the regulation of host metabolism during SADS-CoV infection and indicated that genes involved in signal transduction, cell growth and death, and immune response were upregulated. Other than this, transcription analysis on SADS-CoV infection is still limited.

In the present study, the Vero E6 cells, an African green monkey kidney cell line that is effective for the isolation and culture of SADS-CoV [2], were collected at 12, 18, and 24 hpi to perform transcriptomic profiling via high-throughput RNA-Seq. Our results complemented transcriptome data for SADS-CoV infection in the early stage, revealing significant changes in multiple regulators including mTOR signaling, PI3K-Akt signaling, MAPK signaling, and cytokine-cytokine receptor interaction. Interestingly, our data showed that the global gene expression tends to be a down-regulated pattern after SADS-CoV infection in Vero E6 cells, which is probably related to the pathogenicity and lethality of the virus. This study may provide a basis for further understanding the pathogenesis of SADS-CoV infection and contribute to the search for candidate genes for therapeutic agents or vaccines.

## 2. Materials and Methods

### 2.1. Cells and Viruses

Monkey kidney cell line (Vero E6) were grown in Dulbecco’s modified Eagle’s medium (DMEM, Gibco) containing 10% fetal bovine serum (Gibco), 100 U/mL penicillin, and 100 mg/mL streptomycin. SADS-CoV strain GDS04 was isolated and propagated in our laboratory, which was described by our previous study [16]. The 50% tissue culture infectious dose (TCID_50_) of virus strain is 1 × 10^6^/0.1 mL.

### 2.2. Western Blotting and TCID_50_ (50% Tissue Culture Infectious Dose) Assay

First, 1 × 10^6^ Vero E6 cells were seeded in 6-well plates per well and infected with SADS-CoV (MOI = 1); each group was performed as three biological triplicates. Cell samples were then collected at 12, 18, and 24 h post infection (hpi), followed by disruption with lysis buffer (Beyotime, P0013J). Then the lysate was centrifuged at 13,000× *g* for 10 min at 4 °C to generate a supernatant containing the extracted protein. The protein concentration was measured using a bicinchoninic acid (BCA) protein assay kit (Beyotime, P0009). A 50 μg portion of each sample was electrophoresed on polyacrylamide gel (10%) and transferred onto a polyvinylidene fluoride membrane (Millipore, IPVH00010). After blocking with 5% skim milk, the blots were incubated overnight at 4 °C with diluted primary antibodies against SADS-CoV-N protein (produced in our laboratory). The membrane was washed three times in Tris-buffered saline with Tween-20 (0.1%) and incubated with HRP-conjugated anti-mouse IgG antibody (dilution, 1:5000) for 1 h at room temperature. The membrane was washed three times for 10 min each time, and the immunoreactive proteins were detected using an enhanced chemiluminescence western blotting detection kit. The GAPDH protein served as an internal control (Abcam, ab128915, 1:6000).

Tissue culture infective dose 50 (TCID50) analyses of the supernatants from each experiment were performed using standard protocols [17].

### 2.3. Immunofluorescence Microscopy

Vero E6 cells were infected with the SADS-CoV at an MOI of 1 for 12, 18, or 24 h. Cells were fixed with ice-cold methanol for 15 min and then washed with phosphate-buffered saline (PBS) and followed by incubating with Triton X-100 (0.5%) for 15 min and PBS washing. After blocked with 1% BSA for 1 h, cells were incubated with primary antibodies against SADS-CoV N protein for 1 h at room temperature. After washing, cells were incubated with a 1:500 dilution of fluorescence-conjugated secondary antibodies for 30 min and then stained with 4′,6-diamidino-2-phenylindole dihydrochloride (DAPI; Invitrogen) for another 10 min. The cells were finally washed and observed using a fluorescence microscope (Nikon) with 40× magnification.

### 2.4. Samples Preparation and RNA Extraction

Vero E6 cells that seeded in six-well plates (1 × 10^6^ cells/well) were infected with SADS-CoV strain GDS04 at an MOI of 1 for a period of 12, 18, and 24 h, and cells which added with the same volume of DMEM were considered as mock-infected at three time-points. After infection, cells were washed with 1× phosphate-buffered saline (PBS) followed by total RNA extraction using the TRIzol reagent (Thermo Fisher, Shanghai, China).

### 2.5. Library Preparation and RNA-Sequencing

The mRNAs were purified by oligo(dT)-attached magnetic beads and then were fragmented into small pieces with a fragment buffer. The first-strand cDNA was generated using random hexamer-primed reverse transcription, followed by second-strand cDNA synthesis. Afterward, A-tailing mix and RNA Index Adapters were added by incubating to end repair. The cDNA fragments obtained from the previous step were amplified by PCR, and products were purified by Ampure XP beads, followed by dissolution in the EB solution. The products were assessed using an Agilent Bioanalyzer 2100 (Agilent Technologies, Santa Clara, CA, USA). The double-stranded PCR products from the previous step were heated, denatured, and circularized by the splint oligo sequence to get the final library. Then final library was amplified with phi29 to make a DNA nanoball (DNB) which had more than 300 copies of one molecule. DNBs were loaded into the patterned nanoarray and single end 150 base reads were generated on the BGIseq500 platform (BGI-Wuhan, China) [18,19]. 

### 2.6. Sequencing Data Quality Control and Genome Mapping

Quality control of raw reads was performed using the SOAPnuke software [20]. The following reads were filtered: (1) The reads containing the adaptor, (2) the reads whose N content is >5%, and (3) low-quality reads (reads with bases having a quality score <10 as the proportion of total bases in the reads that are >20% as low-quality reads). The filtered clean reads obtained from each sample were about42.4 million, packed in the form of FASTQ, with an average size of 6.23 Gb. Hierarchical Indexing for Spliced Alignment of Transcripts (HISTAT) was used to map RNA-sequencing reads to the reference genome. Firstly, we used HISTAT global FM index to anchor the position of partial sequences in each read on the genome, and then used the partial genome indexes of these alignment positions to align the remaining sequences of each read to extend the alignment area [21].

### 2.7. Gene Expression Analysis

For the quantitative analysis of gene expression, Bowtie2 (V2.2.5) was employed to align the clean reads to the reference gene sequences [22]. The soft parameters were set as: -q --phred64 --sensitive --dpad 0 --gbar 99,999,999 --mp 1,1 --np 1 --score-min L, 0, −0.1 -*p* 16 -k 200. Later, the RSEM (V1.2.8) was used to calculate the gene expression level in each sample by using the default parameters [23]. DEGs were selected by applying the following filtering parameters: |log2 (fold change)| ≥ 1, and Q-value ≤ 0.001.

### 2.8. Gene Set Enrichment Analysis (GSEA)

The GSEA software tool (version 3.0, https://www.gsea-msigdb.org/gsea, accessed on 26 February 2021) was used to determine whether a predefined set of genes statistically showed significant and consistent differences between the two groups. Briefly, an enrichment score was calculated for each gene set (KEGG pathway) by ranking each gene by their expression difference using Kolmogorov-Smirnov statistic, computing a cumulative sum of each ranked in each gene set, and recording the maximum deviation from zero as the enrichment score.

### 2.9. Gene Ontology (GO) Term and Pathway Enrichment Analysis of DEGs

GO term and pathway enrichment analysis was performed using the GO: TermFinder [24] and KEGG pathways database [25], respectively. For GO terms analysis, we mapped all candidate genes to each term in the GO database, calculated the number of genes in each term, and then applied the hypergeometric test to find GO terms that were significantly enriched in candidate genes compared to the background of all genes of the pig. The calculated *p*-value is corrected by Bonferroni. The calculation method to predict enriched pathways was the same as described for the GO terms. The GO terms or pathways with Q-value (corrected *p*-value) ≤0.05 were defined as significantly enriched GO terms or pathways corresponding to the candidate genes.

### 2.10. Gene Correlation Analysis

Correlations were calculated using R statistical platform version 3.4.3 (R [26]) and figures were generated using package corrplot [27].

### 2.11. Quantitative Real-Time PCR

To analyze mRNA expression, 0.2 μg of total RNA was used to reverse transcription in order to generate cDNA using the First Strand cDNA Synthesis Kit (TOYOBO) following the manufacturer’s protocol. Quantitative real-time PCR was performed using a LightCycler480 II system (Roche) and the SYBR Green Real-time PCR Master Mix (Yeasen). Amplification was performed for 2 min at 50 °C and 10 min at 95 °C, followed by 40 cycles of 95 °C for 15 s, 60 °C for 15 s, and 72 °C for 30 s. The relative expression values of candidate mRNAs were normalized to that of GAPDH in each sample using the 2^−ΔΔCt^ method. 

## 3. Results

### 3.1. Vero E6 Cells Are Permissive to Infection Caused by SADS-CoV Strain GDS04

**Figure 1:**
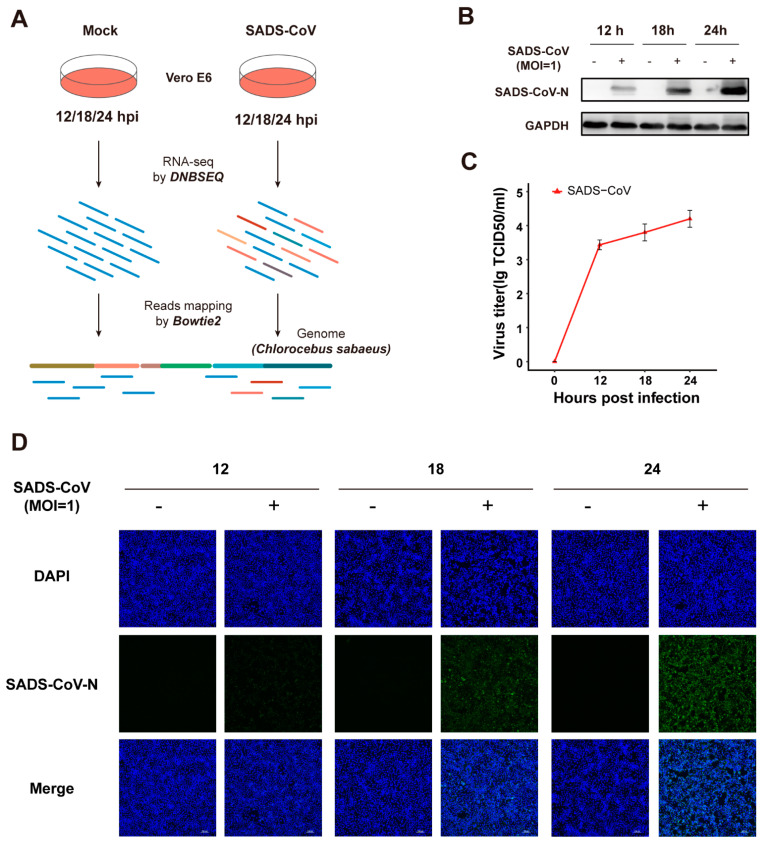
Workflow and confirmation of productive swine acute diarrhea syndrome coronavirus (SADS-CoV) infection in cultured Vero E6 cells. (**A**) Workflow. Vero E6 cells were mock-infected or infected with SADS-CoV strain GDS04 (MOI = 1), followed by sample collection at 12, 18, 24 hpi. Samples from each group were prepared in triplicate. Total RNA was extracted and BGI DNBSEQ RNA-sequencing was performed. (**B**) Western blot confirmation via viral N-protein in SADS-CoV-infected Vero E6 cells. Cells were fixed at 12, 18, or 24 hpi, and N protein was detected by Western blot; GAPDH was conducted as an internal control gene. (**C**) The virus titer of SADS-CoV in Vero E6 cells was measured by TCID_50_. (**D**) Immunofluorescence staining of the viral N-protein in SADS-CoV-infected Vero E6 cells. Cells were fixed at 12, 18, or 24 hpi, and N protein (green) was detected by indirect immunofluorescence assay. Nuclei (blue) were shown by 4′,6-diamidino-2-phenylindole (DAPI) staining. The images of cells were acquired by a fluorescence microscope (Nikon) at a 40× magnification.

To investigate the dynamics of host genes expression during the late stages of the SADS-CoV infection, cultured Vero E6 cells were mock-infected or infected with the SADS-CoV strain GDS04 at MOI of 1 for a period of 12, 18, and 24 h (Figure 1A). To validate whether Vero E6 cells had acquired successful infection, the replication of GDS04 viruses was examined by western blot (WB), and the tissue culture infectious dose 50 (TCID50) assay and the indirect immunofluorescence assay (IFA) were performed. It was observed that GDS04 replicated successfully in a time-dependent manner as assessed by an increase in the quantity of SADS-CoV N protein (Figure 1B,D) and an increase in the production of infectious viral particles (Figure 1C). In both assays, the GDS04 strain was observed to replicate efficiently (Figure 1B–D). Overall, these results indicate the establishment of SADS-CoV strain GDS04 infection in cultured Vero E6 cells.

### 3.2. Transcriptomic Analysis of the SADS-CoVstrain GDS04-Infected Vero E6 Cells

To examine the reproducibility and the specificity of each group, the gene expression levels were used to conduct a principal component analysis (PCA) for each biological replicate. Every sample from the same group was clustered closely together, which suggested that the reproducibility of each treatment was satisfactory, and the specificity between groups is apparent (Supplementary Figure S1B). 

In order to visualize the transcriptomic profile of the GDS04-infected cells, a total of 18 sequencing libraries prepared at 12, 18, and 24 h post-infection (hpi) were sequenced, and ~763 million raw reads were obtained. The number of clean reads ranged from ~36 to 44 million after filtering out the adapter and low-quality reads, and the composition of clean reads for each sample was shown in a stack barplot (Supplementary Figure S1A). To this end, a total of 18,198 genes were detected; among them, 5048 genes were considered to be significantly differentially expressed genes (DEGs) in infected samples when compared to the mock-infected samples under the parameter of q-value ≤0.001 and |log2 (fold change)| ≥ 1. Overall, the genes in each infected sample exhibiting the down-regulated expression pattern constitute ≥50% of the total DEGs identified, and the increase in the number of down-regulated genes is dependent on the time of infection. The precise numbers of DEGs at 12, 18, and 24 hpi were as follows: 35, 1676, and 2885 down-regulated and 20, 111, and 321 up-regulated genes in the GDS04-infected Vero E6 cells, and the was presented in scatter plot (Supplementary Figure S2). Taken together, these data demonstrate that the infection of cultured Vero E6 cells by SADS-CoV GDS04 strain induces widespread alterations in the expression pattern of cell genes. 

Venn diagrams were generated to examine the unique and overlapping DEGs among subgroups with the infection at three infection time-points (Supplementary Figure S1C). To this end, 45 DEGs were noticed as common among all three subgroups in the infection groups, including immune-associated genes, such as IFIT2 and SOCS3.

### 3.3. Gene Expression Trend Analysis

**Figure 2:**
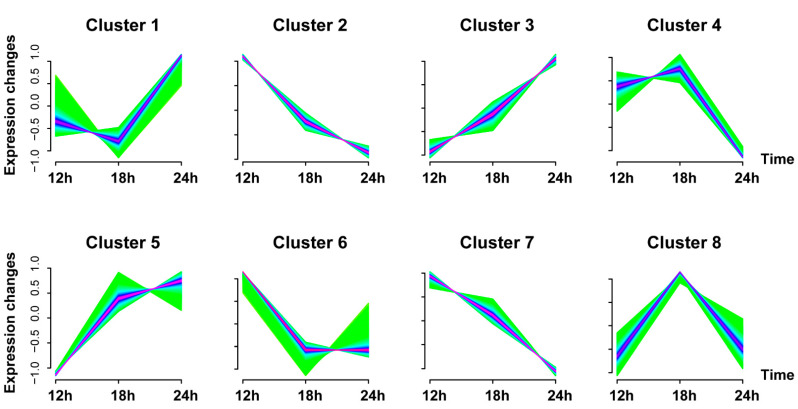
Graphs of eight clusters in SADS-CoV-infected Vero E6 cells along time series based on FPKM value of genes at 12, 18, and 24 hpi. Genes in the same cluster have a similar expression trend.

To determine how Vero E6 cells respond to the SADS-CoV infection, 16,179 genes identified in the virus infection groups were classified into 8 clusters using the Mfuzz package (V2.48.0) by considering their expression tendencies along with the time (Figure 2). The genes classified in clusters 2, 4, 6, 7, and 8 were down-regulated, whereas the genes in clusters 1, 3, and 5 demonstrated up-regulation. Those genes that clustered together were more likely considered to be classified into the same functional gene set.

### 3.4. Gene Ontology (GO) Enrichment Analysis of DEGs

**Figure 3:**
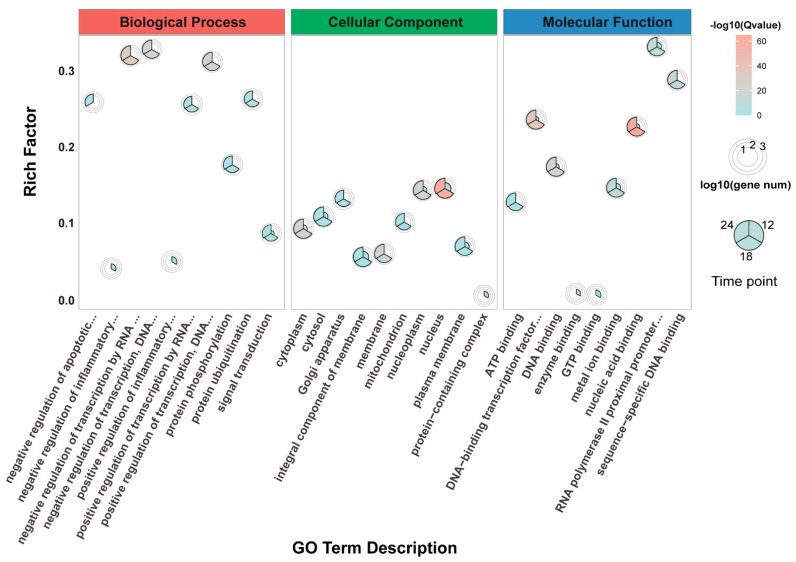
Gene ontology enrichment analysis of DEGs in SADS-CoV-infected Vero E6 cells at 12, 18, and 24 dpi. The circles of different sizes indicate the log10 value of gene numbers in each GO term. The circular sectors in different positions of circle indicate three time-points.

To explore the biological functions of the DEGs, GO enrichment analysis was performed in researching three functional categories including biological process, cellular component, and molecular function. The top ten or less highly enriched GO terms (*p*-value < 0.05) at three time points were considered (Figure 3). The categories: negative regulation of inflammatory response (GO:0050728) and positive regulation of inflammatory response (GO:0050729) were only enriched at 12 hpi in SADS-CoV-infected Vero E6 cells. With the process of infection, DEGs that belonged to all three functional categories were broadly enriched, and the gene numbers of each GO term as well increased over time. DEGs that are associated with negative regulation of apoptotic process (GO:0043066) were significantly enriched at 24 hpi.

### 3.5. GSEA Pathways Enrichment Analysis of DEGs

**Figure 4:**
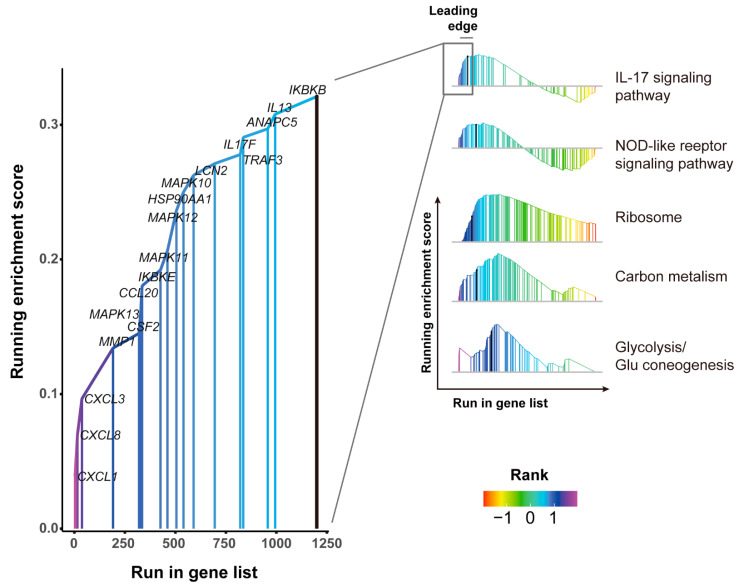
Rank-based gene set enrichment analysis of genes that were regulated upon SADS-CoV infection. The *X*-axis always indicates the rank of the change value. The change values are sorted in descending order from the left to right; namely, the maximum change value is sorted as 1. The colored fold line indicates the change curve of the gene enrichment score (ES), and the *Y*-axis is the ES value. The leading edge genes subset indicates a strong relation to the pathway.

Gene set enrichment analysis identified 120 pathways that were regulated by SADS-CoV infection among three time-points, such as IL-17 signaling pathway, NOD-like receptor, ribosome, carbon metabolism, and Glycolysis/Gluconeogenesis (Figure 4). The leading edge subset of IL-17 signaling pathway consists of several inflammatory-related genes (CXCL1, CXCL3, CXCL8, CCL20, IL13, IL17) and MAPK signaling pathway-related genes (MAPK10, MAPK11, MAPK12, MAPK13), indicating that SADS-CoV infection initiated intense inflammatory response and signal transduction.

### 3.6. Pathways Analysis of DEGs

**Figure 5:**
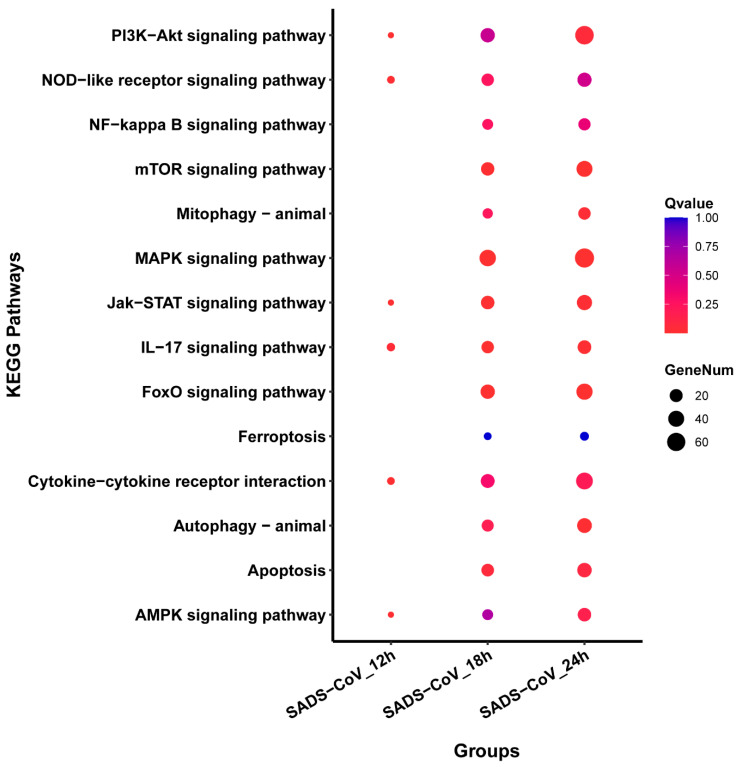
Dot map showing the compilation of top 14 enriched Kyoto encyclopedia of genes and genomes (KEGG) pathways in all SADS-CoV infection groups. Each vertical column of the dot represents a SADS-CoV infection group.

To identify the cellular pathways potentially involved during the SADS-CoV infection, the Kyoto encyclopedia of genes and genomes (KEGG) database was employed. The top ten highly enriched biological pathways corresponding to each condition in both infection groups are shown together in a bubble diagram (Figure 5). During the viral infection, the KEGG pathways were observed to be enriched in the immune-associated pathways such as Jak-STAT and IL-17 signaling. In contrast, the SADS-CoV infection resulted in marked induction of apoptosis- and autophagy-related pathways, and NOD signaling. The most commonly enriched pathways among all the infection conditions are PI3K-Akt and MAPK signaling. 

**Figure 6:**
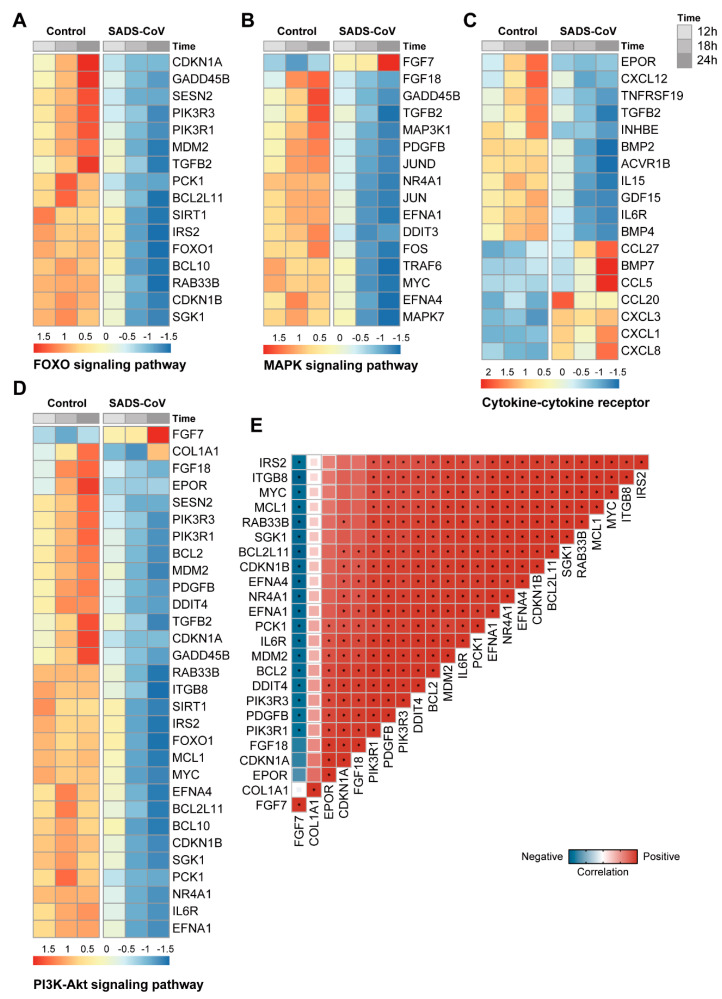
(**A**–**D**): Heatmap shows the fold change of genes in four KEGG pathways in SADS-CoV-infected cells compared with mock-infected cells at 12, 18, and 24 hpi. (**E**): Correlation plot shows the correlations between genes enriched in PI3K-Akt signaling pathway. The color and size of square denote the correlation coefficient between two genes and * represents *p*-value of correlation >0.05.

In order to further analyze the host immune defense response during SADS-CoV infection, heatmaps were used to show the gene expression trend in cytokine-cytokine receptor interaction, FOXO signaling pathway, MAPK signaling pathway, and PI3K-Akt signaling pathway (Figure 6A–D).In addition to further analysis of the genes involved in PI3K-Akt pathway, the correlation between genes in PI3K-Akt signaling pathway is shown in a correlation plot (Figure 6E). Most genes have a strong positive correlation with others, and their correlation *p* values with each other are greater than 0.05 except FGF7, COL1A1, EPOR, CDKN1A, and FGF18. Nonetheless, FGF7 has a negative correlation with other genes.

**Figure 7:**
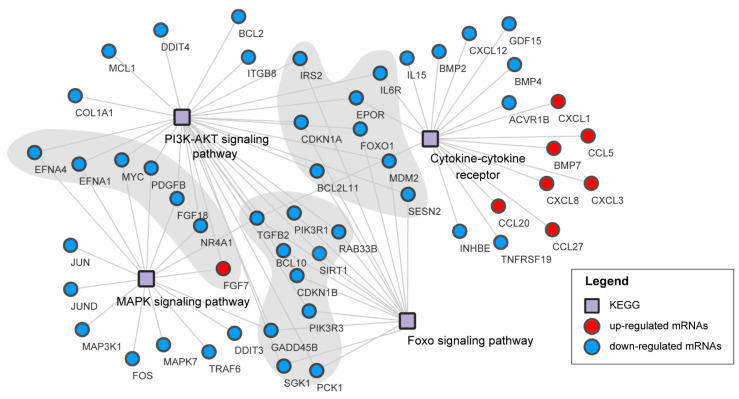
The connections between four KEGG pathways. The box represents KEGG pathway, the circle represents gene, and the circles on the shadow represent the common genes involved in two KEGG pathways.

To investigate the gene and pathway interaction, we performed gene-pathway interactions using the genes enriched from cytokine-cytokine receptor, FOXO signaling pathway, MAPK signaling pathway, and PI3K-Akt signaling pathway (Figure 7). The result showed that these pathways were closely related and most of genes were down-regulated up to our RNA-seq data except the genes from cytokine-cytokine receptor. These findings may suggest that the host immune defense response was suppressed after virus infection.

**Figure 8:**
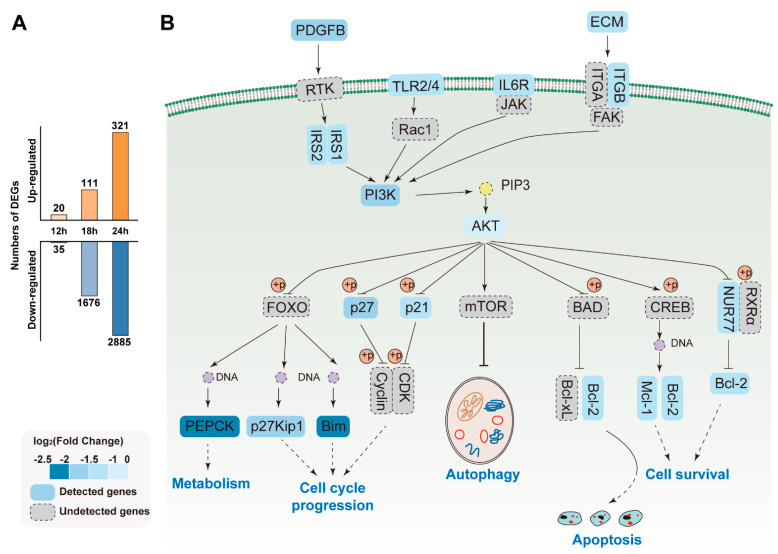
Signaling pathways affected by SADS-CoV infection. (**A**) Barplot of DEGs number in SADS-CoV infection cells at three time-points. (**B**) Schematic representation of the signaling pathways that converge on autophagy, apoptosis, cell cycle progression, and metabolism. The proteins detected in SADS-CoV-infected cells are shown in the solid boxes, whereas the proteins that remained undetected in our study are shown in the dotted boxes. Boxes in different shades of color represent proteins fold change.

In order to understand the host immune defense response upon SADS-CoV infection, the most enriched immune-signaling pathways were displayed in Figure 8B and the numbers of DEGs were also shown in Figure 8A. The statistic of DEGs revealed that most of the host genes were down-regulated after virus infection. Besides, the down-regulation of PI3K and the down-regulation of AKT, Bcl2, and Bim were noticed in a time-dependent manner. Overall, these findings predict that the increase of vRNA or viral protein may alter the transcription level of host genes with the replication of SADS-CoV.

### 3.7. Confirmation of DEGs by Quantitative Real-Time PCR

**Figure 9:**
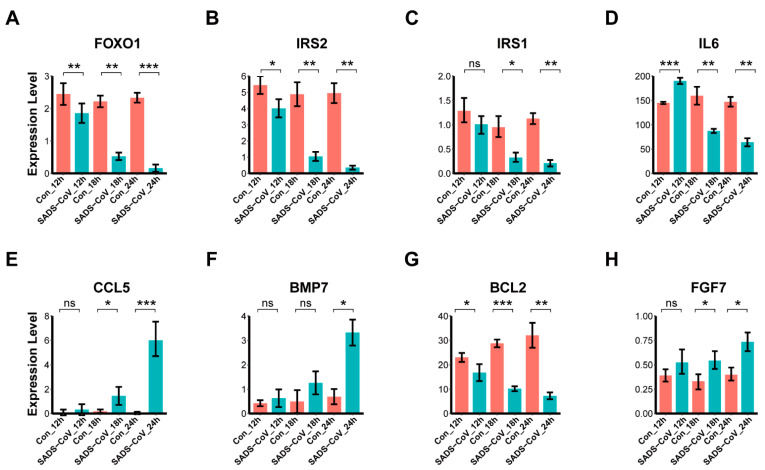
Quantitative real-time PCR validation of RNA-sequencing data. (**A**–**H**) Barplots of representative genes expression level in Vero E6 cells validated by quantitative real-time PCR, including (**A**) FOXO1, (**B**) IRS2, (**C**) IRS1, (**D**) IL6, (**E**) CCL5, (**F**) BMP7, (**G**) BCL2, (**H**) FGF7. Cultured Vero E6 cells were mock-infected or infected with SADS-CoV strain GDS04. At 12, 18, and 24 hpi, samples were harvested and relative mRNA expression levels of indicated genes were measured by quantitative real-time PCR. Data are expressed as mean ± SEM from three independent experiments. Data were analyzed using the Mann-Whitney test. ns represents not significant, *p* > 0.05, * represents 0.01 < *p* < 0.05, ** represent 0.001 < *p* < 0.01, *** represents *p* < 0.001.

To confirm the differential expression of the SADS-CoV-induced genes detected in our sequencing data, we chose eight DEGs (FOXO1, IRS2, IRS1, IL6, CC5, BMP7, BCL2, and FGF7) as predicted above to be engaged in immune response, apoptosis, and autophagy, and then validated their expression in SADS-CoV-infected or mock-infected Vero E6 cells by quantitative real-time PCR. As shown in Figure 9, these DEGs exhibited similar expression signatures as were detected in the sequencing data, indicating the robustness of our experimental settings and bioinformatic analyses. Primers used in this study were shown in Supplementary Table S1.

## 4. Discussion

First emerging in 2017, SADS-CoV caused a remarkable outbreak of diarrhea in neonatal piglets, leading to significant economic losses in the porcine industry [1,2,3]. As a newly discovered coronavirus, the research on SADS-CoV in recent years have been mainly focused on etiology, molecular epidemiology, genetic evolution, and clinical diagnosis [3,28,29,30]. The interaction between SADS-CoV and host is rarely discovered. Exploration of the overall changes of gene expression after pathogen infection via RNA-seq technology would provide clues for the study of host-pathogen interaction. Analysis of the differences in gene expression profiles after SADS-CoV infection could provide basic data for a better understanding of the viral pathogeneses and molecular mechanism. Herein, we directed our attention to host transcriptome during early infection and identified a total of 3324 significant DEGs between the infected and uninfected samples at different time-points (12, 18, and 24 hpi), which were predicted to mainly regulate signal transduction, cellular transcription, immune and inflammatory response, and autophagy. The findings obtained from this study highlighted the intricate regulation of cellular genes during the initial phases of SADS-CoV infection and suggest their potential roles in regulating the SADS-CoV pathogenesis. Further studies are required to understand the biological impact of identified DEGs during SADS-CoV infection. Whether or not targeting these genes confers a therapeutic effect against SADS-CoV infection remains to be evaluated in future studies.

Interestingly, the global gene expression showed a down-regulated pattern after SADS-CoV infection, suggesting that the virus may cause the loss of resistance of the host against pathogens by downregulating the gene expression. We hypothesize that SADS-CoV has a mechanism of immune evasion in Vero E6 cells, which prevents the activation of the immune-related genes in the early stages of infection. On the contrary, Zhang et al. [12] found more up-regulated genes in the transcriptomic profile of SADS-CoV infection while they obtained the DEGs in IPEC-J2 cells after 6, 24, and 48 hpi. The difference may be due to the different cell lines and virus strains used; in particular, Vero E6 cells could not produce type I interferon(IFN) in response to viral infections and are more susceptible to SADS-CoV than IPEC-J2 cells [13,31]. Antagonism of IFN signaling is a survival strategy of the virus against the host-innate immune response to favor of its own infection. SADS-CoV infection antagonized IFN-β production via blocking IPS-1 and RIG-I to impede the activation of IRF3 in IPEC-J2 cells [32]. SADS-CoV N protein obstructed interactions between TRAF3 and TBK1, resulting in failure of TBK1 activation and in turn reducing IFN- β production [33]. Another RNA sequence in HEK-293T cells found that two types of genes were obviously down-regulated under the action of SADS-CoV nsp1, one of which belongs to IFN signaling [34]. Therefore, in the IFN-deficient Vero cells, virus-host competition may be skewed toward the virus or even amplify this bias, thus showing a pattern of down-regulated host gene expression. The role of FOXO, mTOR, MAPK, cytokines receptor, and PI3K-AKT pathways is widely known in regulating IFN signaling [35,36,37,38]; however, most of the genes related to the pathways were down-regulated in our results, which may be due to the deficiency of IFN in Vero cells. Furthermore, we observed an elevated expression of CCL5 in the Vero cells, which is inconsistent with a previous study indicating that type I IFN induces CCL5 expression in human pulmonary vascular endothelial cells [39]. Such differences may be attributable to cell/tissue-specificity or may occur due to the activation of CCL5 via the IFN-independent pathways that are yet undefined. Hence, the establishment of new SADS-CoV-permissive and IFN-expressing cell lines and the exploration of host gene expression profiles in such cells could provide a better understanding of SADS-CoV biology.

Autophagy is a biological process that maintains intracellular homeostasis and facilitates resilience when facing environmental disturbances. Since infectious agents represent a major type of environmental threat, the autophagy pathway is the core of host-pathogen interactions [40]. The KEGG pathway analysis of infected Vero E6 cells at 12 and 24 hpi indicated that the DEGs were highly enriched in the autophagy pathway. Gene products of other coronaviruses, such as SARS-CoV, MERS-CoV, MHV, IBV, TGEV, and PEDV, activate the autophagy pathway through a variety of mediators to promote/inhibit virus replication in infected cells [40,41,42,43,44,45,46]. Thus, we speculated that autophagy plays an important role in the process of viral infection. Moreover, mTOR and PI3K-Akt pathways were also markedly modulated during the course of the SADS-CoV infection according to our results. Appelberg et al. observed significant changes of Akt/mTOR signaling at the proteo-transcriptomic levels in Huh7 cells in response to SARS-CoV-2 infection, while inhibition of the mTOR signaling pathway with an Akt inhibitor showed a significant reduction in virus production [47]. In addition, Lin et al. found that PEDV induced autophagy to facilitate viral replication via the PI3K/Akt/mTOR signaling pathway in IPEC-J2 cells, indicating that autophagy played a proviral role in viral infection [48]. Given the importance of these regulations in coronavirus infection, dysregulation of the PI3K/Akt/mTOR pathway might enhance the pathogenicity of SADS-CoV. Clearly, further mechanistic research to elucidate the precise functions are warranted.

Cytokines and chemokines play crucial roles in immune system development and homeostasis and are involved in protective or destructive immune and inflammatory responses. Under the stimulation of infection and inflammation, cytokines and chemokines can be produced in large quantities, which transmit signals to the cell through the corresponding cytokine receptors [49]. Analyzing the cytokine-cytokine receptors’ interaction in the KEGG pathway, we observed significant changes in the expression of many important cytokines and cytokine receptor genes. Chemokines CXCL1, CXCL3, CXCL8, CCL5, CCL20, and CCL27 were found to be up-regulated in response to SADS-CoV infection, which are generally classified as inflammatory and/or homeostatic. Moreover, CXCL8 was also up-regulated in other coronavirus infections in vivo or in vitro, such as in infections with MERS-CoV, SARS-CoV, SARS-CoV-2, and PEDV [50,51,52,53,54].

In conclusion, we extensively characterized the transcriptome of Vero E6 cells in the early stages of SADS-CoV infection. Integration of functional analysis revealed that differentially expressed transcripts are associated with signal transduction, cellular transcription, immune and inflammatory response, as well as autophagy. In addition, signaling pathways such as PI3K/Akt pathway and cytokine-cytokine receptor interaction may play major roles in host-virus interaction. This study may shed light on molecular mechanisms involved during the SADS-CoV pathogenesis and serve as a valuable resource for SADS-CoV-related research studies in the future.

## Data Availability

Obtained and analyzed data of this study are available from the corresponding author on request.

## Supplementary Materials

The following are available online at https://www.mdpi.com/article/10.3390/v13040674/s1, Figure S1: Basic information of RNA-seq results, Figure S2: Scatter plot of DEGs for each time point, Table S1: Primers used for quantitative real-time PCR analysis of DEGs.
