# Supplementary material for: Transcriptional Landscape of Vero E6 Cells during Early Swine Acute Diarrhea Syndrome Coronavirus Infection
Source: Viruses. 2021 Apr 14;13(4):674. doi: 10.3390/v13040674 (PMC8070899; doi:10.3390/v13040674)
Supplement: Supplementary file 1 [file viruses-13-00674-s001.pdf]

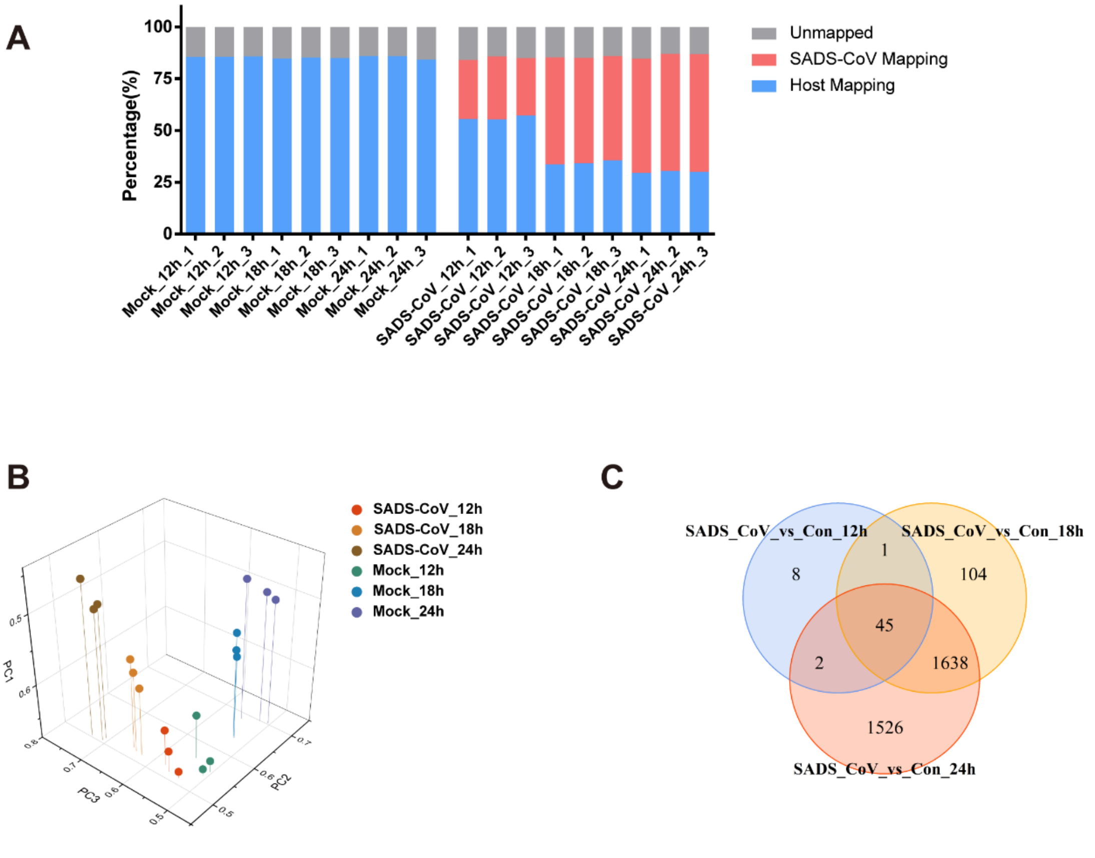

Figure S1. Basic information of RNA-seq results. (**A**) Bar plot of reads component for each sample. (**B**) Dot plot shows the principal component analysis (PCA) of each sample. (**C**) Venn diagram of DEGs for three time points, the overlap part of the circles represents common differentially expressed genes between combinations.

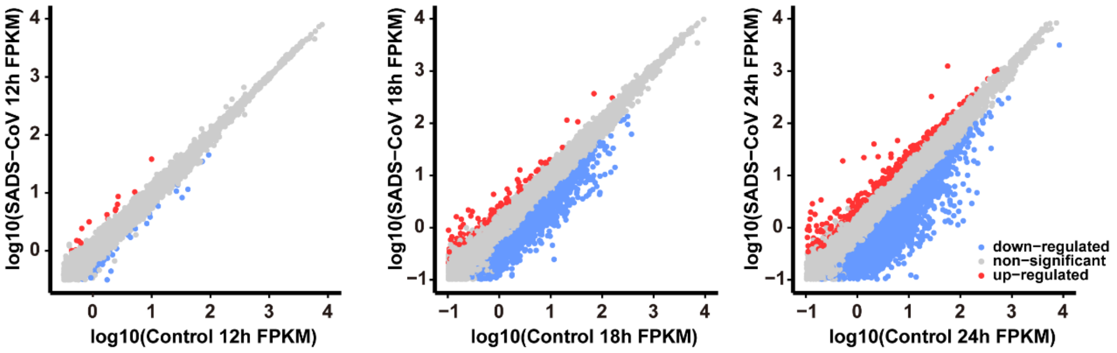

Figure S2. Scatter plot of DEGs for each time point. The X and Y axes represent the logarithmic value of gene expression. Red represents up-regulated DEG, blue represents down-regulated DEG, and gray represents non-DEG.

Table S1. Primers used for quantitative real-time PCR analysis of DEGs.

**Gene**	**Primer**	**Sequence**
FOXO1	Forward	5′-CTACGAGTGGATGGTCAAGAGC-3′
	Reverse	5′-CCAGTTCCTTCATTCTGCACACG-3′
IRS2	Forward	5′-GACGTCCGGCATCAGAGAAA-3′
	Reverse	5′-TCCACGGCTAATCGTCACAG-3′
IRS1	Forward	5′-CACAACCAGGACCTCACACC-3′
	Reverse	5′-CTTGGCACGATAGAGAGCGT-3′
IL6	Forward	5′-AGGATACCACTCCCAACAGACCT-3′
	Reverse	5′-CAAGTGCATCATCGTTGTTCATAC-3′
CCL5	Forward	5′-TCACGTTGTACGCAGCTACC-3′
	Reverse	5′-CAGTCCTCTTACAGCCTTTGG-3′
BMP7	Forward	5′-CTGTGCAGCCGCAGTGCCTACC-3′
	Reverse	5′-ATCCCTCCCCACCCCACCATCT-3′
BCL2	Forward	5′-CTCTCCCCCTCGACTTCTGA-3′
	Reverse	5′-AGTCACGCGGAACACTTGAT-3′
FGF7	Forward	5′-CTGTCGAACACAGTGGTACCTG-3′
	Reverse	5′-CCAACTGCCACTGTCCTGATTTC-3′
GAPDH	Forward	5′-GGGAGCCAAAAGGGTCATCA-3′
	Reverse	5′-CGTGGACTGTGGTCATGAGT-3′

Supplementary material: Differentially expressed genes

**Gene Symbol**	**log2(SADS-CoV_12h/ Control_12h)**	**Qvalue (SADS-CoV _12h/ Control_12h)**
PTGER4	-1.03693	6.79E-04
TMEM72	-3.08132	3.66E-04
IFIT2	-1.02918	2.11E-07
FRAT2	-1.09282	4.66E-05
GPR155	1.006876	3.67E-29
PTHLH	-1.19751	6.56E-05
TPM2	1.067257	4.44E-14
GAS1	-1.29488	1.55E-44
CYP26B1	2.495422	1.57E-28
ZFP36L2	-1.02052	4.82E-10
RHOB	-1.03925	6.54E-255
ID2	-1.28355	1.29E-55
LOC103222352	-2.03652	1.52E-126
SMAD7	-1.07002	3.57E-35
ASXL3	-1.06278	2.74E-10

ADORA2A	1.046097	1.81E-15
S100A8	2.047963	2.56E-04
DMRTA2	4.147499	8.75E-04
SLFNL1	2.093051	3.71E-04
KLHDC7A	-1.11579	1.91E-42
RHBDL1	2.288361	4.67E-07
TMEM60	-1.05044	4.33E-04
AMT	1.23133	1.53E-04
GPHB5	5.147499	2.31E-07
LOC103229154	-4.49877	4.50E-30
DNAH3	-1.45983	3.04E-04
ZNF670	-1.27877	1.58E-04
LOC103232367	7.601016	7.85E-18
LOC103233166	1.48547	1.41E-05
ZNF555	-1.26431	3.41E-07
ZBTB3	-1.15206	1.62E-05
JUNB	-1.05293	2.68E-152
SERTAD3	-1.12179	1.42E-22
TMEM190	1.138729	3.54E-05
CXCL1	1.941048	1.17E-11
PIGY	-1.77229	5.78E-133
PYURF	1.884112	4.02E-136
TIFA	-1.00972	3.80E-04
LOC103237505	3.147499	1.89E-04
SKIDA1	-1.29062	9.65E-06
OTUD1	-1.37998	7.77E-07
LOC103238388	-2.11634	1.61E-07
WSCD2	-1.13454	1.22E-04
CD24	-1.04833	6.74E-208
SCARF1	1.267631	4.67E-26
LOC103242710	1.734597	3.13E-18
LOC103243474	1.431292	5.42E-04
SOCS3	-1.48097	2.69E-46
PCK1	-1.271	8.24E-07
CASS4	-2.86373	3.15E-06
ADRB2	-1.00985	7.77E-61
ZNF879	-1.0489	4.93E-06
SPTBN5	1.006636	6.42E-06
CHAC1	-1.33327	1.79E-11
LOC103248152	-2.43139	4.89E-04

Gene Symbol	log2(SADS-CoV_18h/Control_18h)	Qvalue(SADS-CoV_18h/Control_18h)
USPL1	-1.0255	1.14E-33
STARD13	-1.24947	2.69E-164
SERTM1	-1.31557	7.08E-06
FOXO1	-2.06866	1.54E-37
KBTBD6	-1.75047	1.34E-12
KBTBD7	-2.44597	1.00E-17
TSC22D1	-1.94803	4.39E-201
COG3	-1.03151	1.80E-22
LRCH1	-1.04443	2.49E-18
HTR2A	-1.17653	1.20E-12
MED4	-1.17165	2.80E-25
TRIM13	-1.93221	1.44E-12
INTS6	-1.00863	9.49E-24
ALG11	-1.41085	5.08E-35
THSD1	-1.35999	2.29E-21
FLRT3	-2.36106	0
DACH1	-1.52162	1.57E-10
BORA	-1.26912	5.33E-51
KLF5	-2.72157	1.12E-157
RNF219	-1.99898	1.44E-33
SPRY2	-3.28436	1.70E-175
SLITRK5	-1.66132	8.30E-172
ZIC5	-1.28693	5.98E-10
ZIC2	-2.99543	3.44E-111
EFNB2	-1.71327	4.71E-59
ABHD13	-1.33291	9.37E-30
IRS2	-2.21852	4.35E-108
ING1	-2.3861	3.35E-49
ZNF133	-1.91312	5.71E-30
CHAMP1	-2.05402	6.57E-122
LRRC14B	-2.5191	2.73E-05
CCDC127	-1.67648	4.25E-27
AHRR	-1.26857	1.01E-65
SLC6A18	-2.73209	2.02E-06
CMBL	3.72907	7.05E-05
ANKRD33B	-1.38594	6.47E-25
ZNF622	-1.03769	1.30E-27
RIN2	-1.55024	1.35E-29
GOLPH3	-1.19179	8.37E-151
GDNF	-1.88622	1.98E-31
PTGER4	-2.0072	2.60E-06

CARD6	-1.16088	4.03E-17
NIM1K	-1.78472	7.28E-22
HMGCS1	-2.17836	0
C4H5orf34	-1.02532	9.10E-26
LOC103215245	-1.65634	2.60E-04
ZNF596	-1.54767	1.44E-10
KBTBD11	-1.60264	1.50E-51
LOC103215277	-1.55152	1.19E-05
KMT5B	-1.70496	3.47E-79
PPP1R3B	-3.50954	2.14E-166
CLDN23	-1.67211	4.36E-07
PRAG1	-2.39187	4.64E-18
LONRF1	-1.7769	5.99E-113
NXT1	-1.33942	1.15E-16
C8H8orf58	-2.2699	2.27E-53
TNFRSF10D	-1.22959	2.64E-10
NKX3-1	-1.31706	2.67E-17
GNRH1	-1.98702	3.60E-06
TRIM35	-1.09192	8.83E-24
PURG	-3.01387	2.97E-08
FUT10	-1.46597	3.90E-30
RNF122	-1.35512	4.73E-13
BRF2	-1.4921	2.56E-27
BAG4	-1.16009	2.16E-31
ZNF343	-1.09239	1.54E-26
KAT6A	-1.60999	8.22E-116
STK35	-1.6521	0
DLC1	-1.97408	4.93E-86
TMEM72	-2.31706	1.64E-05
DEPP1	-4.24214	0
ZNF22	-1.36656	8.01E-24
LOC103215804	1.229838	4.31E-05
LOC103215829	-1.52074	1.59E-44
ZNF488	-1.66255	1.26E-05
FAM110A	-2.58949	2.61E-16
ZNF503	-3.38714	2.94E-90
SAMD8	-1.10523	6.89E-57
KAT6B	-1.00209	9.21E-14
TBC1D20	-1.44641	3.89E-60
DDIT4	-3.60358	0
CHST3	-1.56111	7.77E-09
ADAMTS14	1.146782	9.52E-05
TET1	-2.68903	1.83E-04
SIRT1	-2.24347	6.27E-71

JMJD1C	-1.10599	9.37E-69
NRBF2	-1.48354	3.15E-58
ADO	-1.00542	7.21E-40
ARID5B	-2.39039	0
IPMK	-1.49656	3.07E-61
MTRNR2L5	1.946553	6.78E-30
CSTF2T	-1.6706	6.27E-55
IFIT2	-2.36825	1.78E-23
IFIT5	-1.05556	2.47E-04
HTR7	1.23607	2.01E-06
ANKRD1	-1.68903	7.34E-05
PPP1R3C	-3.80272	4.62E-256
HHEX	-2.14963	2.13E-16
LOC103216270	1.852868	4.40E-05
LOC103216306	-1.37393	1.39E-05
CCNJ	-1.12726	1.73E-23
ZNF518A	-1.28526	1.28E-56
FRAT1	-2.41017	2.80E-07
FRAT2	-2.20757	9.37E-12
DNMBP	-1.37445	1.96E-49
SEMA4G	1.203366	3.06E-08
HPS6	-2.67197	1.04E-82
PPRC1	-1.73565	6.21E-86
C9H10orf95	-3.93003	1.16E-06
TAF5	-1.74897	7.85E-22
TGIF2	-1.28022	6.24E-21
ITPRIP	-2.11176	1.13E-15
DUSP5	-2.70183	1.35E-59
RBM20	-1.0852	1.61E-05
ADRA2A	4.355369	3.65E-04
SOGA1	-1.57592	3.32E-52
CASP7	-1.39303	1.90E-39
ADRB1	-4.73209	8.92E-12
FAM160B1	-2.07307	8.05E-64
EMX2	-1.97608	3.25E-109
BAG3	-1.51008	1.80E-107
IKZF5	-1.16546	1.01E-22
ZRANB1	-1.32712	7.64E-59
SON	-1.1287	0
TCF7L2	-1.13577	5.20E-17
DNAJC28	-1.46132	2.66E-08
ABRAXAS2	-1.05741	5.99E-25
SFT2D3	-2.05967	2.59E-20
MAP3K2	-1.21152	6.33E-73

TMEM177	-1.91234	4.32E-81
TMEM37	-1.62005	7.71E-17
INSIG2	-1.01206	8.77E-29
LOC103216964	-1.31923	2.27E-04
CCNT2	-1.04712	3.39E-44
ACVR2A	-1.18267	1.94E-25
EPC2	-1.09169	3.44E-30
LYPD6	-1.03695	4.63E-05
RND3	-2.11904	1.20E-106
SCAF4	-1.47752	1.13E-92
BAZ2B	-1.0978	7.00E-32
7-Mar	-1.13318	1.01E-96
FIGN	-1.54029	3.06E-33
SP3	-1.03748	9.66E-93
GPR155	1.550185	9.89E-63
HOXD13	-1.19572	6.66E-17
HOXD9	-1.05636	2.58E-17
HOXD3	-1.22959	1.64E-07
HOXD4	-1.44337	5.87E-24
NFE2L2	-1.33446	6.90E-199
LOC103217397	-2.79446	4.14E-20
LOC103217405	-1.9128	3.67E-18
PLEKHA3	-1.28574	4.01E-11
BACH1	-2.40372	1.88E-110
DUSP19	-1.61864	4.95E-04
MARS2	-1.19337	3.52E-25
C10H2orf69	-1.99854	7.09E-44
CLK1	-1.78778	1.12E-80
FZD7	-1.19823	5.13E-26
FAM117B	-1.15623	3.31E-14
RAPH1	-1.06994	7.74E-12
ADAMTS1	-1.85054	0
INO80D	-1.33604	1.69E-20
FZD5	-1.12268	2.40E-14
PIKFYVE	-1.1674	8.14E-46
IKZF2	-1.34889	7.00E-09
RNF25	-1.3979	1.89E-20
CDK5R2	-2.25344	2.36E-11
KCNE4	-2.30325	3.30E-39
MRPL44	-1.30078	2.04E-50
IRS1	-1.51647	4.32E-10
HTR2B	-1.91765	5.33E-13
B3GNT7	-2.68752	2.26E-99
TIGD1	-1.83226	1.21E-11

GIGYF2	-1.0705	2.88E-53
HJURP	-1.52107	1.73E-85
LOC103218119	-2.06417	2.72E-33
C2H21orf91	-1.21232	5.92E-04
ARL4C	-1.80481	4.57E-280
GBX2	-1.46566	4.94E-06
KLHL30	-1.22959	1.04E-04
PER2	-1.24157	1.84E-05
NRIP1	-1.44943	1.64E-183
LOC103218231	-3.81576	3.91E-06
MTERF4	-1.47686	9.31E-40
LOC103218293	-1.69526	2.06E-04
LOC103218361	-1.94873	3.29E-04
FBXL14	-1.44447	4.45E-36
DCP1B	-1.58819	9.67E-83
PLEKHG6	-1.09089	5.04E-46
LPAR5	-1.3834	1.55E-12
RUNX1	-1.41886	9.14E-99
LRP6	-1.20845	8.77E-53
DUSP16	-1.20191	2.43E-70
CDKN1B	-2.95301	2.30E-241
GPRC5A	-1.98549	1.65E-39
ATF7IP	-1.24934	1.15E-30
DYRK1A	-1.52961	5.31E-97
KCNJ8	-1.85739	6.90E-223
KRAS	-1.0912	1.28E-35
RASSF8	-1.03377	6.64E-99
PTHLH	-2.22959	4.73E-12
RESF1	-1.97434	1.63E-27
ETS2	-1.33495	2.16E-64
MEGF9	-2.31476	7.99E-83
TRIM32	-2.31488	5.11E-116
HDHD3	-2.18446	2.49E-44
ZFP37	-2.82454	8.36E-17
UGCG	-1.26836	0
PTPN3	-1.36126	1.49E-186
KLF4	-3.34507	1.10E-07
ZNF189	-1.91472	1.82E-23
MSANTD3	-1.1448	1.59E-40
ALG2	-1.28601	6.51E-68
SHB	-1.27885	6.47E-164
ZBTB5	-1.99153	3.94E-47
GNE	-1.42278	3.04E-86
HRCT1	-2.1249	6.93E-23

RIPK4	-2.39952	8.94E-16
TPM2	1.506194	6.38E-35
FAM214B	-1.20389	4.65E-49
DNAJB5	-1.47245	1.73E-54
CCL27	1.376742	1.73E-08
ZBTB21	-1.39033	1.41E-41
TOPORS	-1.81863	3.16E-91
ACER2	-1.10406	2.28E-07
RRAGA	-1.36777	2.40E-145
RANBP6	-2.18926	5.87E-127
KIAA2026	-1.46986	1.35E-50
LOC103219486	-1.16013	2.39E-08
CDC37L1	-1.19553	4.31E-21
PLPP6	-1.94949	5.11E-48
VLDLR	-1.85022	3.65E-64
LOC103219534	-2.16819	3.67E-12
FAM122A	-1.77189	8.65E-34
SIK1	-3.24054	5.37E-279
KLF9	-2.17388	1.06E-31
ABHD17B	-2.07691	5.12E-33
ZFAND5	-1.82931	0
RMI1	-1.27248	4.51E-28
GAS1	-3.80594	2.34E-141
SHC3	1.075261	3.37E-05
NFIL3	-2.93862	1.55E-162
ZNF484	-1.92147	9.77E-10
ZNF658	-1.44319	5.03E-16
ZNF367	-2.34298	1.19E-61
PTCH1	-2.56065	5.40E-32
LOC103219907	-2.03695	9.64E-06
ZNF782	-1.01809	1.40E-05
KDM3A	-1.18645	3.78E-82
DOK1	-1.07553	2.82E-21
PCGF1	-1.34507	3.19E-19
TET3	-1.32646	6.68E-29
CYP26B1	3.446517	3.00E-66
MXD1	-2.25806	1.73E-48
PLEK	1.009872	9.85E-07
ETAA1	-1.25934	8.30E-43
MEIS1	-1.06965	3.26E-05
SPRED2	-1.09683	3.33E-82
SERTAD2	-2.34659	8.68E-253
AFTPH	-1.05255	1.53E-38
PELI1	-1.17431	1.39E-104

LOC103220168	-1.62398	4.72E-06
SLC19A1	-1.09601	4.72E-09
OTX1	-2.28849	1.70E-22
B3GNT2	-1.66228	3.12E-74
REL	-1.37735	6.92E-06
PAPOLG	-1.66093	5.37E-36
BCL11A	-1.67438	1.21E-06
STON1	-1.2228	4.59E-31
FOXN2	-1.11983	1.18E-16
SOCS5	-1.79758	1.46E-64
PRKCE	1.309272	1.33E-16
SIX2	-1.96656	6.95E-04
SIX3	-2.73209	3.29E-08
C1GALT1C1L	-1.47195	8.79E-15
ZFP36L2	-3.2846	9.09E-48
PKDCC	-1.20687	2.86E-27
CYP1B1	-1.05306	2.93E-19
YPEL5	-1.35462	1.65E-54
FOSL2	-2.32558	0
DNAJC27	-1.81941	5.11E-39
WDCP	-1.26191	1.43E-24
GDF7	-1.37708	1.15E-14
RHOB	-3.21248	0
PUM2	-1.68505	3.93E-229
OSR1	-2.10406	1.84E-04
RDH14	-1.41775	1.92E-21
LRATD1	-2.02858	7.40E-63
TRIB2	-2.25997	1.24E-49
E2F6	-1.41693	3.61E-37
KLF11	-1.94009	1.50E-98
LOC103220837	1.557003	4.57E-04
ID2	-3.36592	1.58E-125
ALKAL2	-1.91765	6.41E-21
LOC103221001	-2.17054	1.73E-08
CSRNP1	-2.75787	1.78E-114
ACVR2B	-1.44422	8.15E-69
SLC22A14	1.895937	8.21E-04
EPM2AIP1	-1.42878	2.76E-59
TBCCD1	-1.26455	2.26E-54
MAGEF1	-1.0696	8.14E-29
EPHB3	-1.36452	8.93E-06
B3GNT5	-1.81456	2.50E-35
KLHL24	-1.57624	8.19E-12
ZMAT3	-1.10809	9.30E-23

GHSR	5.355369	2.21E-04
SKIL	-1.99008	5.54E-161
PELO	-2.79831	4.57E-238
ZNF639	-2.12096	6.66E-48
GCLC	-1.08527	4.26E-99
GCM1	-2.76011	1.39E-06
PAQR8	-1.24473	1.63E-18
SNX18	-1.59584	1.11E-45
LOC103221425	-1.96656	9.69E-07
RBM4B	-1.3969	6.20E-26
C17H6orf223	-1.28022	2.26E-04
MAD2L1BP	-2.21592	6.38E-53
BICRAL	-1.50748	3.29E-37
TBCC	-2.28353	2.64E-38
SAYSD1	-1.13648	2.51E-05
TBC1D22B	-1.43748	1.51E-59
PIM1	-2.85063	1.48E-160
CDKN1A	-1.20102	0
BRPF3	-1.10685	6.13E-48
MAPK13	1.355369	3.85E-04
IP6K3	-2.09541	7.75E-65
ZBTB9	-2.65462	6.42E-60
KIFC1	-1.4508	9.76E-113
MAP3K1	-2.41893	0
FKBPL	-2.17976	2.49E-26
MIER3	-2.06585	1.19E-65
ZBTB12	-1.00891	2.31E-07
HSPA1B	-2.60426	0
HSPA1L	-2.25934	3.31E-05
HSPA1A	-2.47253	1.90E-154
NFKBIL1	-1.05438	6.29E-29
LOC103221768	-1.28514	3.70E-10
IER3	-2.56868	0
PLK2	-2.40965	0
PPP1R10	-2.90543	0
TRIM26	-1.63169	2.10E-31
PDE4D	-1.45199	1.20E-132
ZNF311	-1.47245	2.09E-04
ZBED9	-1.38786	1.02E-07
ZKSCAN4	-1.50261	1.04E-06
ZNF165	-4.22959	1.19E-04
ZSCAN16	-2.25344	3.30E-06
ZKSCAN8	-1.27002	2.56E-51
LOC103221947	-1.25544	3.55E-04

ZNF391	-1.28849	6.54E-05
ZNF184	-1.89761	9.69E-11
ZNF322	-1.60429	2.71E-29
ABT1	-1.42514	1.08E-29
LOC103222011	-1.16736	2.11E-29
LOC103222014	-1.36923	1.58E-12
ZSWIM6	-1.03085	2.81E-23
E2F3	-1.60341	1.72E-30
KIF13A	-1.02855	2.81E-18
NUP153	-1.23542	6.23E-117
MYLIP	-2.28022	1.64E-08
JARID2	-1.53142	1.24E-20
EDN1	-2.93003	4.97E-194
HIVEP1	-1.68416	1.33E-47
NEDD9	-2.40772	3.60E-33
TFAP2A	-2.08309	3.94E-105
SNRNP48	-1.00355	2.97E-11
RREB1	-1.36246	9.31E-134
PPP1R3G	-1.09714	2.22E-10
PXDC1	-1.23729	1.05E-41
FOXC1	-1.87217	2.83E-38
FOXF2	-1.71238	2.38E-39
FOXQ1	-3.85408	9.12E-17
TAF8	-1.33495	6.16E-04
LOC103222346	-1.05967	1.15E-04
LOC103222352	-1.0216	4.22E-22
ZSCAN12	-2.74351	1.65E-15
LOC103222374	-1.28185	5.64E-30
LOC103222379	-1.59216	6.62E-04
SMAD4	-1.05862	2.37E-52
ELAC1	-1.21098	3.85E-09
LIPG	-2.13788	1.76E-21
SMAD7	-3.62707	1.22E-133
MAST4	-1.04385	1.21E-22
ZNF397	-1.13367	3.92E-11
ASXL3	-2.17237	7.19E-29
KLHL14	-2.76011	1.39E-06
RNF138	-1.50261	2.29E-19
PIK3R1	-2.4158	0
TGIF1	-2.51937	0
LOC103222706	-2.77391	1.06E-19
ZBTB14	-1.06467	1.82E-17
ANKRD12	-1.08435	1.73E-43
CHMP1B	-1.15401	3.10E-98

LOC103222806	2.763246	1.06E-04
CEP76	-1.38278	1.65E-18
CECR2	-1.52342	3.11E-12
USP18	1.023525	5.17E-05
HIC2	-1.34754	3.72E-13
CRKL	-1.33183	1.11E-99
ZNF74	-1.24896	2.53E-09
TMEM171	-2.04456	3.12E-15
ZNF70	-1.62648	2.25E-05
FOXD1	-1.41415	1.00E-23
PHRF1	-1.12371	2.46E-75
ADORA2A	1.042037	3.10E-15
ASPHD2	-1.17515	1.60E-04
MN1	-2.26679	6.94E-71
ENC1	-1.11199	3.38E-12
CCDC117	-1.59662	4.71E-67
XBP1	-1.99324	3.76E-221
CASTOR1	-1.67705	2.49E-11
GAL3ST1	-1.28849	9.78E-10
DUSP18	-1.11452	1.51E-09
PATZ1	-1.14424	2.34E-43
EIF4ENIF1	-1.97463	3.36E-141
PRR14L	-1.30251	5.34E-108
DEPDC5	-1.23266	1.14E-51
HMGXB4	-1.74459	4.15E-88
HMOX1	-1.11957	6.42E-50
RASD2	-1.0978	1.82E-28
HMGCR	-1.87203	0
C1QTNF6	-1.05967	1.76E-06
ELFN2	-1.11499	1.21E-51
H1F0	-1.15928	0
PDGFB	-2.15332	2.18E-304
F2RL2	-1.36204	3.04E-08
F2RL1	-1.12304	5.85E-72
EP300	-1.48777	2.80E-276
TEF	-2.12005	2.11E-135
TOB2	-2.96225	6.94E-153
TCF20	-1.30154	1.31E-87
RTL6	-1.6519	6.19E-134
BRD1	-1.4706	5.79E-67
PIM3	-1.11928	1.41E-21
ZBED4	-2.75225	1.13E-84
TNRC6B	-1.2155	4.62E-26
HSPA6	-2.96656	2.72E-19

MPZ	-1.16489	1.22E-04
PIGM	-1.31803	1.90E-08
TMEM151A	-1.42753	2.45E-06
ETV3	-1.93765	7.21E-80
PRCC	-1.26002	2.40E-70
BGLAP	1.1271	7.74E-04
PAQR6	1.468579	1.92E-07
MEF2D	-1.2481	1.37E-32
MEX3A	-1.42663	9.38E-14
HCN3	1.091211	3.07E-17
EFNA1	-2.27561	5.95E-58
EFNA3	-1.28022	5.10E-11
ZBTB7B	-1.30038	5.51E-58
EFNA4	-2.01387	3.53E-19
IL6R	-1.60201	2.98E-31
GATAD2B	-1.14247	2.47E-40
S100A8	1.402674	1.55E-04
RORC	-1.36084	6.50E-05
SNX27	-1.16336	5.98E-06
POGZ	-1.25356	4.25E-88
ZNF687	-1.72567	9.47E-114
TXNIP	-3.42585	0
ANKRD34A	-2.64463	1.48E-07
PEX11B	-1.36084	1.82E-16
PDZK1	-1.70886	3.87E-80
CD160	-1.0718	3.46E-18
ZNF697	-2.58082	1.13E-20
VCAN	-1.02169	0
VANGL1	-1.09157	2.21E-38
NGF	-1.39842	3.57E-101
RSBN1	-2.50545	7.11E-98
LRIG2	-1.28618	5.25E-19
CTTNBP2NL	-1.3951	1.12E-64
DDX20	-1.99143	2.54E-68
LRIF1	-1.3302	3.36E-32
RBM15	-1.61548	1.16E-11
AMIGO1	-2.61864	4.21E-12
ATXN7L2	-1.54275	2.32E-09
GPSM2	-1.13725	2.13E-45
PRPF38B	-1.4567	7.76E-129
FAM102B	-1.36842	1.87E-19
VAV3	-1.88792	4.77E-105
SASS6	-1.44264	3.25E-57
MFSD14A	-1.28817	6.94E-87

F3	-2.04098	1.21E-27
ARHGAP29	-1.19635	0
ZNF644	-1.03369	3.10E-39
LYSMD3	-1.41299	7.83E-69
RBMXL1	-1.92438	6.43E-60
LOC103224490	-3.8443	8.34E-22
LMO4	-1.38481	2.44E-97
ZNHIT6	-1.17984	6.03E-24
CCN1	-1.70745	0
BCL10	-2.07146	4.84E-97
C20H1orf52	-1.78213	2.61E-28
SYDE2	-1.05735	1.53E-18
ARRDC3	-2.96186	0
RPE65	-2.25934	3.31E-05
GADD45A	-1.28364	1.31E-43
PDE4B	-2.85179	9.51E-70
NR2F1	-1.22271	1.74E-08
JUN	-3.54051	0
PARS2	-2.1346	4.40E-46
CPT2	-1.3161	6.99E-78
KTI12	-2.20942	5.17E-36
CDKN2C	-1.26047	2.37E-48
DMRTA2	6.677297	3.02E-09
TRABD2B	-1.30602	0
FOXD2	-2.02725	2.17E-12
PIK3R3	-1.24694	1.53E-28
CCDC17	1.878931	5.48E-05
TOE1	-1.12398	9.28E-16
ZNF691	-1.54275	1.69E-13
FOXO6	-1.5097	1.50E-13
LOC103225021	-2.48593	3.93E-05
ZNF684	-1.4289	1.16E-06
RLF	-2.27656	1.42E-144
MFSD2A	1.008147	7.68E-11
MYCL	-1.14106	4.35E-06
LOC103225049	2.331366	2.53E-14
MTF1	-1.67765	9.03E-39
LOC103225095	-3.83915	1.21E-31
ZSCAN20	-2.04318	2.48E-18
TRIM62	-1.12036	8.57E-04
IQCC	-2.21752	1.06E-20
COL16A1	1.076215	1.18E-07
PUM1	-1.88307	0
YTHDF2	-1.8761	1.29E-177

GMEB1	-1.37422	7.04E-15
PHACTR4	-1.1259	1.56E-80
MED18	-1.01387	4.53E-14
SESN2	-2.31858	6.55E-269
PPP1R8	-1.37516	3.46E-80
FAM76A	-1.31517	1.16E-08
AHDC1	-1.84757	4.88E-116
TENT5B	-2.09152	1.25E-18
LOC103225309	-1.5771	2.20E-17
ARID1A	-1.67622	4.73E-253
PDIK1L	-2.47245	2.03E-14
PAQR7	-1.15871	1.21E-14
AUNIP	-1.49263	5.29E-08
SRRM1	-1.08349	1.72E-162
IFNLR1	-1.20535	3.73E-05
PNRC2	-2.92142	1.45E-244
ID3	-2.28094	4.94E-123
ZNF436	-1.43645	9.83E-39
LUZP1	-1.43858	2.08E-98
C1H11orf68	-1.51039	1.11E-73
MUL1	-1.95206	2.74E-77
FAM43B	-1.63121	8.54E-13
IFFO2	-1.3481	4.00E-50
KLHDC7A	-2.50067	5.47E-107
ARHGEF10L	-1.22009	1.05E-43
FBXO42	-1.47971	1.83E-28
SPEN	-1.72296	1.07E-177
FOSL1	-1.26497	2.09E-33
PRDM2	-1.4716	9.53E-57
TNFRSF8	-1.93741	1.83E-04
RHBDL1	3.442832	4.81E-18
CLCN6	-1.10016	1.04E-50
UBIAD1	-1.54487	5.97E-23
SPSB1	-2.01589	1.79E-18
ERRFI1	-2.14229	1.53E-212
PER3	-1.24938	2.41E-41
THAP3	-1.15135	3.38E-25
PHF13	-2.38879	9.52E-110
KLHL21	-1.74129	2.23E-95
C20H1orf174	-1.83509	1.55E-37
SKI	-1.92559	2.70E-101
ANKRD65	-1.80669	8.91E-82
LOC103225820	-2.1852	1.85E-11
PLEKHN1	1.361764	5.95E-13

LOC103225896	-2.45199	5.66E-05
STIL	-1.48151	1.32E-52
LOC103225945	-2.74437	7.14E-59
RNF19B	-1.30952	8.21E-21
LOC103225955	-1.32816	3.76E-18
LOC103226004	-2.74015	9.39E-39
ZNF273	-2.33113	3.87E-37
ZNF107	-1.58608	5.76E-10
ZNF680	-1.95396	4.44E-17
FIGNL1	-1.88685	1.06E-51
PURB	-1.08926	5.53E-71
LOC103226149	-1.05749	5.77E-63
HECW1	-1.31706	3.99E-05
LOC103226163	-1.55817	5.67E-40
INHBA	-1.0331	0
CRAMP1	-1.54638	1.28E-43
CDK13	-1.05433	8.13E-134
KBTBD2	-1.46142	1.50E-129
AVL9	-1.2638	1.13E-91
EME2	1.450006	1.67E-08
HOXA13	-2.42599	3.96E-06
HOXA1	-1.20884	2.78E-08
HOXA3	-2.23591	3.43E-39
HOXA4	-1.3184	2.05E-06
HOXA5	-2.16807	1.16E-28
HOXA6	-2.00582	9.94E-26
HOXA9	-1.25196	1.81E-65
LOC103226329	-2.32197	2.69E-07
HOXA10	-1.39024	3.19E-12
SP4	-1.84124	3.19E-75
SP8	-4.61042	1.61E-20
ITGB8	-1.17269	1.36E-20
AHR	-2.15529	2.70E-76
ARL4A	-2.60611	8.20E-23
GLCCI1	-1.29878	2.51E-24
DLX5	-1.65183	2.77E-12
DLX6	-2.10406	4.55E-18
SAMD9	-4.16819	1.79E-04
RBM48	-2.67648	5.99E-48
MTERF1	-1.4377	2.46E-19
FZD1	-1.53562	2.92E-55
DBF4	-1.43925	4.58E-42
RSBN1L	-2.60913	4.60E-29
TMEM60	-1.37255	1.46E-05

NAPEPLD	-1.20466	6.94E-23
KMT2E	-2.04404	5.69E-206
CCDC71L	-2.67438	3.88E-16
HBP1	-2.45889	1.21E-66
CBLL1	-1.05428	6.14E-21
DNAJB9	-1.42284	2.01E-16
THAP5	-1.73548	3.50E-39
LRRN3	-1.60449	3.54E-103
CTTNBP2	-1.61221	8.69E-58
ING3	-1.18895	8.14E-06
ZNF800	-1.01589	2.49E-07
GCC1	-1.26029	3.41E-15
HILPDA	-2.72777	3.60E-210
AP5B1	-1.29864	2.06E-09
CNOT4	-1.15836	1.66E-48
LOC103226962	1.028313	4.88E-78
KAT5	-2.18145	2.74E-127
TRIM24	-1.32626	5.68E-174
TMEM139	-1.0462	7.27E-12
TCAF2	-1.05723	1.34E-06
LOC103227175	-1.87596	1.93E-15
ZNF786	-1.97455	5.72E-14
ZNF398	-2.17742	6.22E-58
ZNF282	-1.1362	4.65E-41
ZNF212	-2.78213	3.91E-76
ZNF205	-1.27561	5.99E-18
ZNF746	-2.01167	6.38E-65
ZNF862	-1.08623	9.76E-19
ZNF775	-2.63228	3.66E-36
KMT2C	-1.12145	2.22E-83
INSIG1	-2.21141	0
ZNF200	-1.3184	1.07E-43
MNX1	-1.02314	1.11E-08
ZNF263	-1.67135	2.22E-50
LOC103227406	-1.88304	3.56E-11
TIGD7	-2.05771	1.82E-19
EIF1B	-1.57972	1.84E-72
ZNF619	-1.28323	2.61E-06
ZNF621	-1.69526	8.74E-08
ZNF174	-1.20102	5.75E-08
SNRK	-1.46188	2.67E-28
ZNF197	-1.28077	2.76E-15
ZNF597	-2.16819	9.33E-05
ZNF35	-1.04492	2.53E-07

SLX4	-1.11412	1.83E-16
CCDC12	-1.0706	9.37E-19
SETD2	-1.38487	6.18E-102
TREX1	-1.66728	2.93E-37
IP6K2	-1.10905	2.49E-49
WDR6	-1.01506	4.36E-201
QRICH1	-1.10923	1.01E-119
GLIS2	-2.42448	1.23E-110
CCDC71	-2.15275	3.59E-40
VASN	-2.32223	5.63E-269
INKA1	-4.03695	4.10E-04
TMEM115	-1.49555	5.75E-82
CISH	-2.11326	1.40E-06
LOC103227687	1.252049	3.73E-30
RBM15B	-2.17817	3.89E-112
DUSP7	-1.73308	3.87E-88
ZNF500	-1.3076	8.40E-05
KCTD6	-1.58323	2.58E-08
ADAMTS9	-1.13702	3.88E-246
KBTBD8	-1.50261	1.04E-06
LOC103227869	2.528737	1.30E-151
FRMD4B	-1.7585	6.04E-110
LOC103227890	-2.26976	1.83E-09
RYBP	-1.28552	2.04E-61
EBLN2	-3.03695	2.01E-08
PPP4R2	-1.02775	6.96E-90
BHLHE40	-3.66876	0
SETD5	-1.06316	7.13E-126
TMEM186	-1.41402	2.53E-24
WNT7A	-1.13283	2.05E-30
CCDC174	-1.9399	1.90E-49
C5H16orf72	-1.0842	3.04E-85
TMCC1	-1.00245	3.22E-52
ATF7IP2	-1.09435	3.41E-25
GATA2	-1.26441	1.18E-27
KLF15	-2.41017	2.80E-07
SLC12A8	4.262259	7.76E-07
MUC13	3.525294	4.14E-04
DTX3L	-1.27646	6.16E-26
WDR5B	-1.41622	1.05E-11
HCLS1	1.577761	7.46E-04
FBXO40	5.525294	8.16E-05
SOCS1	-4.73209	2.02E-06
USF3	-2.15098	6.30E-21

NEPRO	-1.71292	2.94E-73
CBLB	-1.58301	3.53E-116
SNN	-1.63981	5.52E-34
NFKBIZ	-3.26015	0
ZBTB11	-1.49481	4.81E-52
TRMT10C	-1.67298	2.19E-88
FILIP1L	-2.54664	1.38E-115
GPR15	-1.84894	1.92E-78
RIOX2	1.225488	1.15E-38
C22H3orf38	-2.21981	8.08E-74
ERCC4	-1.34393	6.88E-27
LOC103228631	-2.41722	8.12E-05
LOC103228639	-3.76901	5.59E-06
IRF9	-1.58715	2.30E-33
NFKBIA	-2.95152	6.77E-304
INSM2	-1.45199	2.74E-04
NKX2-8	-1.78759	2.33E-04
PAX9	-2.12036	5.01E-08
FOXA1	-2.34811	4.72E-17
FBXO33	-1.77708	3.47E-29
C24H14orf28	-2.13006	2.86E-14
KLHL28	-1.68355	3.95E-16
FANCM	-1.33978	9.94E-18
MGAT2	-1.15318	2.27E-70
DNAAF2	-1.25253	9.55E-13
ARF6	-1.76129	0
SAV1	-1.00901	1.58E-39
BMP4	-3.57537	2.94E-09
FBXO34	-2.7114	2.12E-166
ATG14	-1.84627	5.66E-28
PELI2	-1.56217	1.47E-04
ITPRIPL2	-1.65009	8.08E-16
ARID4A	-1.45389	5.25E-36
SIX1	-1.65844	1.03E-18
SIX4	-1.13767	7.14E-06
TRMT5	-1.6605	2.80E-52
GPHB5	7.309565	2.92E-13
WDR89	-1.30733	2.46E-45
ESR2	4.940331	7.11E-11
AKAP5	2.498444	1.01E-102
ZBTB25	-2.08623	9.41E-32
ZBTB1	-1.00162	1.25E-25
HSPA2	-2.32913	6.88E-72
ZFP36L1	-2.9799	0

ZFYVE1	-1.14182	4.91E-44
ACOT6	-1.04517	1.07E-04
ELMSAN1	-1.8042	4.79E-82
ZC2HC1C	-1.64012	1.17E-18
FOS	-2.29998	1.64E-06
LOC103229355	-1.61936	2.13E-16
GPATCH2L	-1.00011	3.20E-12
IRF2BPL	-2.7455	8.77E-115
DCUN1D3	-1.70475	6.90E-24
STON2	-1.03458	3.49E-05
TIGD3	-2.45199	1.37E-07
NRDE2	-1.86404	3.19E-75
TMEM251	-1.02606	5.16E-09
BTBD7	-1.89637	3.12E-205
SERPINA10	-4.22959	1.19E-04
DICER1	-1.22394	1.79E-127
CDR2	-1.05461	1.87E-35
CDC42EP2	-1.72065	2.30E-34
SCNN1B	1.969478	1.08E-14
WDR20	-1.1112	1.50E-19
BAG5	-1.12913	3.70E-41
CDCA4	-1.74001	3.60E-72
LOC103229802	3.525294	4.14E-04
RBBP6	-1.85879	1.01E-222
TNRC6A	-1.31115	9.11E-119
LOC103229898	-3.22959	5.16E-04
MLH3	-1.46092	3.68E-16
ALKBH1	-1.17664	3.25E-08
HIST3H2A	-1.77995	8.88E-42
TRIM11	-1.40064	3.30E-43
LOC103229978	-1.8045	4.21E-10
ZKSCAN2	-1.77057	1.10E-40
FBXO28	-1.26469	9.24E-68
TLR5	-2.41236	4.47E-37
DUSP10	-1.68416	1.47E-09
HLX	-2.01809	9.11E-18
TGFB2	-1.1614	8.92E-136
ATF3	-1.49366	4.62E-55
NEK2	-1.25255	6.21E-43
TRAF5	-1.04551	2.15E-10
C25H1orf74	-2.4354	5.91E-33
YOD1	-3.20102	9.61E-99
C25H1orf116	-2.12771	1.00E-10
DYRK3	-2.01028	1.37E-20

ELK4	-1.01719	1.55E-12
NUAK2	-3.14814	0
PPP1R15B	-2.4528	3.88E-165
ZBED6	-1.58404	7.72E-43
BTG2	-3.38448	2.78E-172
KDM5B	-2.07298	2.92E-119
ARL8A	-1.50205	1.50E-63
ELF3	-3.49591	0
TNNI1	2.691228	3.19E-15
CAMSAP2	-1.09971	3.27E-75
ZNF281	-2.61601	1.21E-165
LOC103230365	-1.27232	2.31E-45
ZBTB41	-1.0635	2.09E-22
ASPM	-1.34037	2.78E-151
RGS2	-2.68387	6.97E-231
RGL1	-1.36181	7.68E-90
RNASEL	-1.17084	1.32E-14
GLUL	-1.75501	0
IER5	-3.60702	1.94E-72
KIAA1614	-1.12576	2.55E-07
ABL2	-1.74417	4.08E-55
RASAL2	-1.05474	1.52E-25
PIGC	-1.21538	2.56E-22
GORAB	-1.2769	1.16E-11
METTL18	-2.17278	4.53E-39
TADA1	-1.76106	2.69E-62
POGK	-1.36827	4.41E-63
RGS5	1.288254	3.37E-05
RHOU	-1.89256	6.69E-05
CCSAP	-2.21449	9.98E-17
URB2	-1.26578	3.32E-25
EXOC8	-2.69886	2.94E-54
MAP3K21	-1.57624	1.64E-22
PAGR1	-1.07912	1.82E-28
IRF2BP2	-2.95929	0
ARID4B	-1.48097	2.42E-120
ZNHIT2	-1.69908	2.43E-07
ZBTB18	-2.21262	3.52E-112
ZNF771	-1.10406	2.27E-04
ZNF48	-1.75316	8.63E-18
KIF28P	-1.45199	6.88E-07
ZNF670	-2.16819	1.30E-06
ZNF124	-1.71992	1.53E-04
LOC103230923	-1.33052	4.67E-07

LOC103230939	-1.08045	3.15E-36
LOC103230963	-1.4698	2.17E-16
PGBD2	-1.95542	3.76E-10
SRCAP	-1.03353	1.07E-114
H3F3A	-1.32234	4.80E-64
WHAMM	-1.12896	2.75E-09
ZNF592	-1.1716	3.75E-71
ZSCAN2	-1.44461	5.17E-10
AKAP13	-1.25114	0
DET1	-1.07588	1.04E-15
AEN	-1.54756	5.78E-84
SETD1A	-1.17655	5.05E-73
GDPGP1	-2.21232	6.56E-08
ZNF774	-1.07304	2.38E-08
CHD2	-1.7462	2.09E-270
NR2F2	-1.53279	5.38E-24
ARRDC4	-1.57565	7.68E-67
ZNF646	-2.36586	2.48E-63
ZNF668	-2.3671	1.73E-30
LYSMD4	-2.99715	1.59E-17
ASB7	-1.41965	2.00E-36
CHSY1	-1.72436	4.74E-34
SALL2	-2.18749	8.85E-108
ARMC5	-2.13425	5.17E-39
C29H14orf119	-1.21681	1.50E-36
HOMEZ	-2.84726	9.11E-128
NGDN	-1.00748	1.61E-20
ZFHX2	-2.42397	8.68E-34
TICRR	-1.67411	1.62E-51
ZNF267	-1.84167	2.00E-25
SHROOM2	-1.43027	2.90E-75
CDKL5	-1.22342	2.41E-09
BCLAF3	-1.53279	5.38E-24
KLHL34	2.255833	4.08E-07
LOC103231695	-1.36405	2.87E-67
KLHL15	-1.09529	1.51E-20
CREBBP	-1.02574	1.77E-45
MID1IP1	-1.57481	2.53E-51
BCOR	-2.11722	1.90E-112
DDX3X	-1.01446	0
PALB2	-2.10108	1.88E-75
LOC103231885	-1.19581	2.69E-08
ZNF41	-1.26544	2.86E-13
LOC103231911	-1.20942	1.69E-08

SUV39H1	-1.04278	3.28E-28
PIM2	-1.13648	2.88E-15
CCDC120	-1.34507	9.32E-20
UBQLN2	-1.1945	1.01E-64
LOC103232070	1.227605	1.43E-06
SPIN4	-1.08942	9.62E-10
AMER1	-1.37694	7.40E-10
PJA1	-1.16065	2.06E-10
RTL5	-1.11508	3.81E-15
ERCC6L	-2.01908	1.04E-156
CITED1	2.548014	4.80E-05
RLIM	-1.52727	4.50E-42
NEXMIF	-2.59883	3.89E-04
MAGEE1	-1.37454	1.23E-12
LOC103232262	-1.20428	5.13E-56
PABPC5	-1.84477	1.26E-13
PCDH11X	1.247733	2.41E-63
GPRASP1	-1.09019	3.51E-07
PWWP3B	-2.3076	2.37E-04
SIAH1	-1.81706	9.53E-38
CLDN2	-1.06919	2.71E-34
RBM41	1.60472	2.75E-129
TSC22D3	-1.9316	1.30E-223
RTL9	-1.45199	3.47E-08
APLN	1.198643	1.96E-06
BCORL1	-1.40136	5.75E-61
ZNF423	-1.43719	1.77E-05
CNEP1R1	-1.79031	3.75E-45
ZIC3	-2.33853	2.00E-20
LOC103232659	-4.22899	2.97E-08
MCF2	4.355369	3.65E-04
LOC103232720	-1.90247	9.23E-08
SALL1	-2.66206	2.11E-60
ZFP92	-1.93003	1.83E-06
ZFX	-1.18241	1.57E-14
USP27X	-2.61042	1.47E-13
IRX3	-2.71684	4.22E-15
IRX5	-3.3816	2.43E-11
HERPUD1	-1.30524	8.63E-81
RSPRY1	-1.2053	8.32E-101
RASGRP2	1.795941	4.21E-04
PDP2	-1.41739	2.72E-63
RRAD	-2.45199	6.76E-09
CMTR2	-3.16819	8.45E-43

LOC103233166	8.222771	3.31E-22
ZNF19	-1.65844	7.50E-13
ATXN1L	-1.31049	7.36E-106
ZFHX3	-1.08381	2.23E-78
COG8	-1.2402	1.14E-58
LOC103233222	-1.16346	6.66E-07
SMPD3	-1.48113	7.99E-06
DDX28	-2.48704	1.88E-48
THAP11	-1.34361	1.75E-41
C5H16orf70	-1.86598	7.42E-64
ZFP1	-2.13006	4.98E-23
LOC103233308	2.162724	6.42E-04
BCAR1	-1.6492	2.07E-141
MAF	-2.45199	5.66E-05
ATMIN	-1.40741	1.74E-65
MLYCD	-1.04342	2.04E-16
OSGIN1	-1.5652	3.48E-20
CRISPLD2	-1.04999	1.32E-32
FOXC2	-2.41722	1.38E-08
SLC7A5	1.037141	9.34E-164
ZFPM1	-1.49092	2.72E-17
CDT1	-1.63487	6.81E-90
FLRT1	-1.67705	3.38E-06
LOC103233486	1.414262	6.89E-04
MISP	-1.25526	1.38E-59
MIDN	-2.7254	2.49E-158
PCSK4	1.999225	1.06E-05
LOC103233620	-4.45199	2.31E-05
MOB3A	-1.50104	2.30E-34
MKNK2	-1.14606	1.26E-115
GADD45B	-1.46784	5.12E-121
ZNF554	-2.10406	1.39E-09
ZNF555	-2.57274	1.54E-14
ZNF57	-1.76011	7.99E-06
LOC103233665	-2.31706	2.11E-14
LRG1	-1.39952	1.34E-04
PLIN4	-1.92866	3.27E-19
ZBTB7A	-1.73622	3.56E-95
FEM1A	-3.05483	1.54E-105
TICAM1	-2.84103	5.36E-55
SAFB	-1.0177	3.44E-133
SAFB2	-1.18456	3.47E-81
CAMSAP3	-1.46746	1.22E-18
LRRC8E	-1.5717	4.89E-29

ZNF558	-1.70048	2.08E-19
ZNF317	-2.12771	1.44E-39
ZNF699	-2.20535	6.57E-06
ZNF266	-2.81456	9.26E-13
ZNF121	-3.31706	5.95E-14
LOC103233870	-1.45733	3.88E-26
ZBTB3	-5.61042	1.96E-21
S1PR5	-1.59459	1.42E-05
CDKN2D	-1.0271	6.21E-07
ANGPTL8	-1.86186	2.53E-13
SWSAP1	-2.09089	4.13E-26
ZNF627	-2.46585	5.96E-50
ZNF823	-2.95542	2.61E-30
LOC103233944	-1.59696	1.56E-10
LOC103233945	-1.38879	6.06E-04
LRRN4CL	-1.13547	7.03E-18
ZNF136	-1.30359	9.05E-06
ZNF44	-1.73814	2.18E-15
ZNF709	-1.96656	9.69E-07
LOC103233973	-2.64463	9.99E-24
ZNF490	-1.9458	6.87E-06
JUNB	-4.35421	0
INTS5	-2.11787	6.54E-91
IER2	-1.78623	1.05E-88
DNAJB1	-2.79868	0
TUT1	-1.0791	1.10E-17
NOTCH3	-1.06716	1.43E-06
MED26	-2.50045	1.07E-32
HAUS8	-1.29056	1.51E-34
MAP1S	-1.17432	4.49E-46
RAB3A	-1.43313	2.13E-11
IQCN	-1.60043	4.62E-13
JUND	-2.74072	0
GDF15	-1.82834	3.09E-48
ELL	-1.27217	7.73E-31
KLHL26	-1.7587	1.27E-42
ZNF14	-2.31139	4.15E-12
ZNF101	-2.01809	1.96E-09
TSSK6	-4.22959	1.19E-04
ZNF253	-2.0293	5.14E-08
LOC103234239	-3.72358	9.72E-06
LOC103234241	-2.18451	2.48E-13
LOC103234249	-1.98702	2.82E-11
LOC103234255	-2.74897	3.71E-10

LOC103234265	-1.50713	5.74E-05
ZNF430	-1.64463	4.83E-13
ZNF675	-3.03695	5.75E-06
MIER2	-1.20415	3.58E-30
PIAS4	-1.20586	2.85E-30
LDLR	-1.1591	1.13E-188
ZNF441	-2.73209	1.48E-13
ZNF791	-3.82785	2.05E-16
PLEKHF1	-1.84975	1.72E-06
CEBPA	-1.18653	1.86E-14
LOC103234475	-1.08662	1.47E-06
ZNF599	-2.12036	5.01E-08
ZNF792	-3.03695	5.75E-06
KMT2B	-1.51176	1.46E-74
LIN37	-1.60207	7.17E-19
NFKBID	-1.5191	2.06E-05
LRFN3	-1.89461	9.15E-25
SDHAF1	-1.8045	4.21E-10
ZNF565	-2.21449	6.57E-09
ZFP82	-3.1134	1.50E-19
ZNF567	-1.86186	2.53E-13
ZNF382	-1.0271	5.69E-05
ZNF420	-1.73209	3.59E-08
ZNF383	-1.5451	9.67E-07
ZNF875	-2.19153	2.77E-16
ZNF570	-1.12462	2.40E-04
LRFN1	-1.01387	5.17E-10
ZFP36	-3.39952	8.84E-95
EID2B	-1.46746	1.22E-18
EID2	-1.33701	3.22E-19
DYRK1B	-1.37216	1.02E-39
SERTAD1	-2.31448	8.18E-62
SERTAD3	-3.69774	3.92E-92
ZNF574	-2.4767	5.20E-84
ZNF526	-2.79508	3.01E-56
DEDD2	-1.18132	2.46E-46
ERF	-2.23401	3.21E-137
CEACAM8	2.199418	9.99E-05
PHLDB3	-1.54029	3.48E-17
ZNF45	-1.17118	4.35E-09
ZNF221	-1.93741	1.83E-04
ZNF284	-2.49263	6.63E-08
ZNF226	-2.14859	3.81E-27
ZNF235	-1.52915	7.45E-06

ZNF112	-1.75113	3.18E-17
ZNF180	-2.11278	5.57E-14
BCL3	-2.68258	1.01E-115
CD3EAP	-1.00951	1.12E-19
PPP1R13L	-1.04732	6.08E-30
FBXO46	-1.79313	1.07E-17
SIX5	-1.20121	3.18E-32
IRF2BP1	-2.16723	6.96E-61
NOVA2	-1.34865	2.04E-09
CCDC8	-2.07833	2.85E-119
ARHGAP35	-1.04378	8.80E-116
ZC3H4	-2.39045	1.09E-79
BBC3	-3.30138	3.52E-156
BICRA	-1.7619	1.76E-17
DBP	-1.17897	1.08E-06
PRR12	-2.08365	5.78E-92
NUP62	-1.00292	3.53E-70
ZNF473	-1.02699	3.61E-25
CTU1	-1.39357	2.54E-06
ZNF613	-2.55152	9.19E-13
ZNF350	-1.20045	3.93E-06
ZNF614	-2.29998	2.23E-17
ZNF836	-2.04456	3.70E-08
ZNF616	-2.77391	1.55E-08
ZNF766	-1.48335	2.26E-14
ZNF347	-1.13817	1.92E-04
ZNF331	-1.95967	8.30E-16
MYADM	-1.13447	6.32E-32
LENG1	-1.31448	2.06E-09
KMT5C	-1.78456	7.39E-16
TMEM190	1.930871	1.60E-14
ZNF628	-2.70833	2.81E-32
ZNF579	-1.74293	3.93E-21
FIZ1	-1.14713	1.34E-13
ZNF524	-1.58547	2.23E-12
ZNF865	-1.45199	2.10E-21
ZNF784	-2.73209	9.17E-28
ZNF581	-1.8406	1.46E-109
CLP1	-1.97153	2.43E-42
ZNF583	-1.79147	6.16E-05
YPEL4	-2.10406	1.84E-04
ZNF71	-2.61662	1.80E-21
ZNF805	-1.61146	1.07E-05
ZNF304	-1.47315	8.51E-11

ZNF547	-2.48593	3.28E-09
ZNF17	-2.78213	7.31E-20
ZNF549	-1.99759	4.73E-18
ZNF211	-1.58608	7.97E-19
ZNF134	-2.3604	4.38E-43
ZNF530	-1.43313	1.68E-04
ZNF416	-3.77391	7.67E-11
LOC103235422	-2.61146	2.31E-35
ZNF586	-2.3816	1.16E-04
LOC103235431	-2.65844	1.96E-22
ZNF551	-1.97962	5.19E-09
ZNF274	-1.44049	3.67E-18
ZNF8	-2.06913	1.66E-16
ZSCAN22	-2.21752	6.62E-11
ZNF837	-1.28849	6.16E-04
ZNF446	-1.03629	4.96E-16
LOC103235452	-1.88622	2.81E-16
ZBTB45	-1.3227	2.18E-25
MZF1	1.107441	1.65E-08
LOC103235480	-3.16819	7.73E-04
TNKS1BP1	-1.27981	1.45E-106
CEBPG	-1.46399	2.42E-57
ZNF584	-1.0361	1.29E-12
LOC103235581	-3.34507	2.30E-04
CENPC	-1.13072	9.54E-15
REST	-1.34507	1.87E-47
RASL11B	-2.99715	1.59E-17
UTP3	-1.54158	6.44E-63
ANKRD17	-1.3441	4.11E-245
CXCL1	1.392843	2.15E-05
STBD1	-1.82071	6.67E-136
SHROOM3	-1.04291	2.19E-14
CCNG2	-2.9045	2.34E-163
CNOT6L	-1.09669	1.16E-70
RASGEF1B	-2.17149	1.13E-18
FNBP4	-1.35639	3.40E-70
HELQ	-1.33853	5.26E-19
AFF1	-1.50685	1.03E-46
NAP1L5	-1.0014	6.54E-13
TIGD2	-1.86702	7.11E-16
GPRIN3	-1.43262	7.93E-48
KBTBD4	-1.14226	9.45E-42
TET2	-1.8252	6.13E-62
INTS12	-1.27141	9.00E-25

LOC103236086	-1.1306	9.99E-51
SEC24B	-1.51661	1.42E-143
RAPSN	2.071576	1.57E-05
PITX2	-1.03167	7.31E-12
TRAM1L1	-1.99041	1.85E-05
BBS12	-3.10406	1.81E-45
SPRY1	-3.25934	2.12E-25
ANKRD50	-2.04235	3.04E-130
PLK4	-1.74694	3.81E-95
JADE1	-2.22756	0
PCDH10	-1.26912	7.88E-04
PABPC4L	-1.88304	3.56E-11
NOCT	-1.72223	2.21E-48
ELF2	-1.82561	1.77E-76
RAB33B	-1.47006	2.06E-12
MAML3	-1.25934	1.06E-04
IL15	-1.52671	7.93E-17
USP38	-1.43304	5.92E-66
GAB1	-1.04152	7.89E-25
SMAD1	-1.21872	2.42E-24
MMAA	-1.22206	7.71E-08
LOC103236339	2.618403	3.93E-04
PRMT9	-1.39896	5.39E-29
LOC103236354	-1.8327	2.30E-47
FHDC1	-2.76011	1.39E-06
LOC103236415	-1.9378	1.05E-52
HARBI1	-2.56349	6.38E-13
GASK1B	-3.64463	2.02E-05
FNIP2	-1.10406	2.92E-20
DUSP8	-1.99255	3.30E-17
CDKN2AIP	-1.63954	3.22E-32
ANKRD37	-2.3302	1.01E-51
CRY2	-1.11458	1.76E-30
CEBPD	-3.61627	0
SNAI2	-3.71686	6.68E-66
TGS1	-1.14988	1.72E-96
PLAG1	-1.42407	1.96E-51
FAM110B	-1.68026	4.21E-08
RRS1	-1.23344	4.83E-28
VCPIP1	-2.50422	1.13E-86
ARFGEF1	-1.31898	1.79E-161
MSC	-2.64463	1.48E-07
ZFHX4	-1.29115	9.02E-17
ZBTB10	-2.37846	8.24E-111

PAG1	-1.32954	1.47E-21
NECAB1	-1.12576	2.09E-09
RUNX1T1	-1.49616	2.82E-119
RBM12B	-2.08801	1.20E-96
PDP1	-1.14157	2.28E-39
GEM	-1.16241	5.44E-62
TP53INP1	-2.49929	1.11E-124
PLEKHF2	-2.36147	1.53E-87
OSR2	-3.09309	1.33E-16
RNF19A	-1.51221	1.31E-89
KLF10	-2.94528	0
RSPO2	5.8148	1.16E-05
TMEM74	-1.14852	3.51E-12
KCNV1	-2.11278	5.57E-14
TRPS1	-1.05312	4.32E-54
ZHX2	-1.04157	2.95E-24
ZHX1	-1.02705	1.96E-52
FBXO32	-2.03177	0
RNF139	-2.09529	5.63E-190
TRMT12	-2.25934	3.00E-38
SQLE	-1.53102	0
ZNF572	-1.75797	9.13E-15
TRIB1	-3.66584	0
LRATD2	-2.44729	1.58E-29
MYC	-2.31054	0
SLA	-2.55152	1.89E-05
LOC103237505	5.442832	3.74E-08
CHRAC1	-1.28383	4.93E-60
JRK	-1.08175	3.18E-13
TRAF6	-2.01095	4.12E-61
TIGD5	-1.81877	6.44E-23
ZNF623	-1.4758	1.16E-35
ZNF707	-1.61245	3.07E-08
FAM83H	-1.51441	3.75E-16
NRBP2	1.215421	1.56E-63
LRRC14	-1.31008	2.42E-16
KIFC2	1.167899	2.57E-37
FJX1	-2.31696	0
ZNF34	-1.8305	1.27E-18
COMMD5	-1.29352	4.98E-26
ZNF250	-1.65449	8.08E-17
LOC103237660	-1.49808	4.19E-24
KLF6	-3.43739	0
NET1	-1.32444	1.25E-127

IL15RA	1.132976	8.02E-08
KIN	-1.37719	2.91E-24
EHF	1.19589	8.11E-08
LOC103237885	-2.08244	3.07E-04
PROSER2	-1.14377	2.98E-19
RPP38	-1.87729	1.16E-41
SKIDA1	-2.6699	6.33E-13
OTUD1	-2.56349	6.38E-13
BAMBI	-3.44049	5.76E-66
MTPAP	-1.58505	2.44E-84
ARHGAP12	-1.21065	1.78E-48
EPC1	-2.25639	2.28E-83
FZD8	-1.74617	2.77E-07
LARP4B	-1.17694	1.74E-57
PRICKLE1	-2.79041	6.21E-147
ARID2	-1.39068	9.29E-54
SLC38A2	-2.7407	0
AMIGO2	-1.56108	3.41E-33
ASB8	-1.04429	2.58E-17
KANSL2	-1.00668	2.74E-62
CCNT1	-1.49041	1.63E-119
TROAP	-1.30423	5.24E-29
PAX6	-1.53676	3.90E-169
METTL7A	-1.0516	1.39E-17
ACVR1B	-1.58959	7.56E-77
GRASP	-3.03695	5.75E-06
NR4A1	-1.45199	6.10E-05
ZNF740	-1.22192	1.90E-47
MFSD5	-1.4251	2.71E-46
SP1	-1.14685	5.11E-112
HOXC13	-1.52295	2.87E-07
HOXC6	-1.23475	3.38E-17
HOXC9	-1.37567	2.75E-18
DNAJC14	-1.80413	8.51E-62
PYM1	-1.2015	1.41E-25
SUOX	-1.3295	2.28E-23
IKZF4	-1.95156	3.56E-21
ZBTB39	-2.95622	2.49E-48
NAB2	-1.76263	1.26E-47
LGR4	-1.37255	7.66E-64
DDIT3	-1.88519	3.20E-40
9-Mar	-1.48918	5.11E-33
LRIG3	-1.02155	3.35E-20
C11H12orf66	-1.18294	1.56E-22

TBK1	-1.57408	1.53E-44
LEMD3	-1.88203	2.16E-78
HELB	-1.22959	6.33E-05
DYRK2	-1.94125	3.59E-48
MDM2	-1.53281	5.40E-89
FRS2	-1.31206	1.07E-43
PTPRR	1.852868	4.40E-05
THAP2	-1.12412	5.93E-06
ATXN7L3B	-1.81094	7.53E-203
PHLDA1	-2.32575	0
BBS10	-1.35947	1.07E-13
E2F7	-1.72674	1.75E-128
ALX1	-2.10406	5.51E-13
KITLG	1.048187	3.04E-05
DUSP6	-3.02844	2.11E-197
GALNT4	-1.41362	2.68E-32
BTG1	-2.66334	0
NUDT4	-1.26825	1.29E-145
TMCC3	-1.27468	6.48E-11
APAF1	-1.39595	5.22E-23
E2F8	-2.25566	1.10E-132
TCP11L2	-2.10406	1.57E-15
MTERF2	-1.04863	4.41E-07
FICD	-1.10078	3.13E-14
TMEM86A	-1.443	2.15E-08
LOC103239100	-1.03185	7.14E-05
SPTY2D1	-1.3912	2.71E-68
B4GALNT4	1.031139	2.27E-61
TBX3	-2.35833	2.76E-59
BICDL1	-1.05685	3.60E-125
SETD1B	-2.42927	1.38E-41
BCL7A	-1.08405	1.53E-11
RSRC2	-1.17177	2.05E-54
VPS37B	-1.41282	3.77E-78
C11H12orf65	-1.1448	2.94E-14
SNRNP35	-1.65958	3.08E-28
ZNF10	-1.36548	1.45E-08
ZCCHC8	-1.74042	1.63E-63
ZMYND19	-1.11473	1.15E-17
TOR4A	-1.18379	3.46E-04
TPRN	-1.34613	3.97E-29
C12H9orf139	2.342949	3.44E-04
NOTCH1	-1.69462	7.21E-113
SOX6	-1.11909	4.19E-38

C12H9orf116	-1.13179	1.06E-16
PPP1R26	-1.00449	1.95E-77
RALGDS	-2.01547	1.19E-88
GTF3C4	-1.59043	1.79E-57
FAM78A	-1.03695	6.63E-04
PRDM12	-1.65958	1.62E-06
IER5L	-3.47982	2.53E-83
DOLK	-1.29807	1.11E-18
LRRC8A	-1.44244	1.05E-21
FAM102A	-1.53892	1.44E-52
ZNF79	-1.68211	5.29E-10
ZBTB34	-2.96656	1.25E-05
ZBTB43	-2.31706	3.00E-23
ZBTB6	-2.07442	4.08E-63
ZBTB26	-2.15098	5.12E-11
TENT5A	-1.75083	1.85E-10
TPBG	-1.63094	5.01E-150
RWDD2A	-1.26912	4.99E-08
LOC103240069	-2.09983	3.03E-27
CNR1	-2.37799	5.05E-13
PNRC1	-3.5411	0
CASP8AP2	-1.49086	1.63E-24
BACH2	-1.45199	6.45E-06
EPHA7	-1.40949	1.99E-151
LOC103240143	1.516875	8.02E-04
LATS1	-1.5097	5.25E-21
LOC103240189	-1.61864	5.16E-10
FBXO30	-1.76247	6.02E-26
CITED2	-3.30575	0
TNFAIP3	-1.20578	1.17E-69
SLC35D3	2.525294	1.68E-05
SGK1	-2.87559	2.36E-38
SLC2A12	-1.16489	3.12E-08
ADM	-5.54187	5.69E-98
THEMIS	3.525294	4.14E-04
RNF217	-1.60896	1.93E-37
WEE1	-2.79496	2.72E-134
MCM9	-1.82624	6.80E-25
RSPH4A	-1.70352	5.79E-04
TSPYL1	-1.54244	9.07E-85
MARCKS	-1.42159	3.06E-114
REV3L	-1.19466	8.66E-43
ZBTB24	-3.0516	8.85E-36
SESN1	-2.26845	1.24E-61

FOXO3	-1.31613	3.23E-86
CD24	-2.27101	0
ZBTB2	-1.90516	1.81E-24
SYNE1	-1.04186	1.78E-71
FBXO5	-2.37288	2.10E-99
SCAF8	-1.61707	2.30E-107
TULP4	-1.06174	9.54E-34
PRKN	1.305183	2.00E-12
FAM120B	-1.00381	1.77E-49
LOC103241052	-3.96656	7.76E-07
ZNF2	-3.44337	8.78E-23
ITPRIPL1	-1.07945	1.42E-14
NEURL3	-1.89256	3.46E-06
ANKRD23	-1.05967	3.28E-04
SEMA4C	-1.3099	8.19E-26
LOC103241090	-3.22959	5.16E-04
LIPT1	-1.49707	3.39E-04
C14H2orf49	-1.17793	1.81E-39
SOWAHC	-2.48593	1.78E-29
CKAP2L	-1.50244	4.28E-207
LOC103241298	-2.81456	5.87E-05
EIF2AK3	-1.47866	5.59E-76
SHOX2	-1.06106	7.12E-11
CCNL1	-1.35675	4.66E-68
TIPARP	-2.35851	6.61E-260
ARHGEF26	-1.41942	7.52E-11
RAP2B	-2.2149	2.25E-73
P2RY1	-2.03236	1.09E-12
SIAH2	-1.79511	3.95E-47
TSC22D2	-2.63942	2.33E-168
DIPK2A	-1.00633	1.84E-31
ZBTB38	-1.00106	2.60E-95
PXYLP1	-1.21617	7.73E-35
NCK1	-1.12506	1.60E-22
MSL2	-2.13357	1.22E-78
AMOTL2	-2.81954	0
ASTE1	-1.54275	2.32E-09
SATB1	-1.14952	6.40E-27
NR1D2	-1.13675	1.98E-66
OXSM	-1.22959	6.12E-48
EOMES	-1.90097	6.76E-07
TGFBR2	-1.22462	4.55E-114
LOC103241812	-2.55152	2.65E-11
BCL6	-2.5347	1.00E-99

GMNC	-2.48593	3.93E-05
MB21D2	-1.32148	1.36E-38
HES1	-2.90874	4.39E-61
FAM43A	-1.58379	9.00E-23
LOC103241951	-4.7673	1.44E-06
WDR53	-1.52455	3.16E-14
RNF168	-1.37614	4.73E-16
LOC103242077	-1.49012	1.37E-06
GEMIN4	-1.65148	5.45E-48
C16H17orf97	-2.13402	1.43E-12
TLCD2	-1.13402	1.17E-05
SCARF1	2.347008	6.12E-157
MNT	-1.50024	3.87E-23
HASPIN	-2.95967	1.70E-48
SMTNL2	-1.35965	4.26E-30
ZFP3	-1.62552	2.10E-17
ZNF594	-1.95691	5.08E-22
MIS12	-1.77464	9.01E-32
SLC16A13	-1.02114	1.67E-12
PHF23	-2.54348	9.34E-140
KCTD11	-2.59505	2.44E-114
TMEM102	-1.5336	4.51E-21
KDM6B	-1.94185	2.73E-37
PER1	-2.08481	8.55E-41
BORCS6	-2.77708	1.34E-49
SOX18	-2.50261	1.34E-06
ADPRM	-1.09953	7.98E-06
ZNF18	-1.63641	2.66E-20
HS3ST3A1	-1.13408	2.95E-39
HS3ST3B1	-2.42933	1.70E-154
MED9	-1.48188	1.99E-40
RAI1	-1.24093	4.59E-38
ALKBH5	-1.06672	1.07E-80
GID4	-1.22084	3.09E-53
MIEF2	-1.8825	9.59E-68
SMCR8	-1.92445	9.22E-50
FAM83G	-1.39465	3.89E-17
MAPK7	-2.04541	5.32E-71
AKAP10	-1.66719	1.46E-55
NATD1	-1.48709	5.00E-11
LOC103242566	-1.00218	3.23E-04
GMEB2	-1.09268	3.38E-27
RSKR	1.177831	1.40E-22
PROCA1	1.174796	6.80E-05

FAM222B	-2.33409	2.31E-81
PHF12	-1.62445	4.27E-163
NUFIP2	-1.06679	4.53E-257
CORO6	1.20629	4.69E-36
SSH2	-1.1178	5.99E-27
TEFM	-1.16819	1.70E-18
CDK5R1	-1.83531	1.24E-14
LOC103242710	1.193401	6.80E-10
ZNF830	-3.24633	4.27E-76
SLFN5	-1.95852	1.09E-13
SLFN12	-1.9825	1.37E-07
PEX12	-1.23686	1.38E-22
CCL5	2.988353	6.38E-13
HNF1B	-1.98102	0
LHX1	-2.66705	0
GGNBP2	-1.42089	2.21E-144
PPM1D	-2.23286	0
MED13	-1.27731	1.34E-188
PTRH2	-1.09462	1.94E-39
YPEL2	-2.08002	2.90E-37
SMG8	-2.27127	4.33E-153
MTMR4	-1.1021	7.37E-166
VEZF1	-1.75477	1.35E-224
YTHDF1	-2.21023	1.57E-122
COIL	-1.60078	1.36E-128
TOB1	-3.07609	1.42E-153
COL1A1	-3.34507	5.40E-11
DIDO1	-1.54297	7.99E-120
SGCA	-1.17806	9.74E-05
TNRC6C	-1.04673	1.64E-23
JMJD6	-1.12842	2.53E-50
QRICH2	4.162724	2.20E-06
FOXJ1	-1.0709	9.28E-05
TRIM47	-1.25231	1.14E-38
OTOP2	2.618403	3.93E-04
GPRC5C	-1.26005	1.54E-123
SSTR2	2.014432	1.03E-06
CDC42EP4	-2.31775	1.57E-46
GNA13	-1.77281	0
TLK2	-1.02727	1.59E-66
KANSL1	-1.93023	3.05E-258
MAP3K14	-1.58423	3.93E-202
HEXIM2	-1.39187	2.95E-11
HEXIM1	-1.56685	5.60E-233

KIF18B	-1.28682	1.95E-42
PPP1R3D	-3.03695	4.48E-23
FAM217B	-1.38255	8.91E-23
GJC1	-1.02719	1.43E-31
FZD2	-2.48095	0
C16H17orf53	-1.44439	2.46E-44
ARL4D	-2.84103	4.43E-07
RUNDC1	-1.12763	5.58E-24
PLEKHH3	-1.37991	1.25E-28
RETREG3	-1.72158	4.95E-118
KRT19	-1.19185	3.39E-15
LOC103243474	1.525294	1.21E-05
LOC103243485	-2.0709	2.57E-04
GJD3	-1.31923	2.27E-04
RARA	-1.16707	2.46E-43
NR1D1	-2.50813	5.30E-51
GRB7	-2.00324	3.76E-125
PLXDC1	-1.37502	3.69E-04
CWC25	-1.20491	4.18E-35
EPOP	-1.18742	4.93E-17
SP2	-1.11434	3.84E-31
HOXB5	-1.62326	6.76E-23
HOXB4	-1.00818	1.50E-06
HOXB8	-1.88014	4.81E-28
IGF2BP1	-1.84627	4.76E-04
ZNF652	-1.36485	6.98E-12
SOCS3	-4.54348	4.80E-167
CANT1	-1.12098	8.39E-222
CBX2	-1.49834	2.59E-13
CBX8	-2.46372	3.40E-47
CBX4	-2.25633	6.76E-102
PCK1	-2.93741	3.62E-23
TEX19	-2.0852	6.99E-07
BMP7	1.36763	4.14E-09
METRNL	-1.80562	1.20E-41
TFAP2C	-1.75316	1.26E-13
TAOK1	-1.12053	1.10E-136
OXLD1	-1.43313	1.68E-04
CASS4	-1.86702	9.18E-05
AURKA	-1.24149	7.57E-138
CSTF1	-1.58516	2.25E-53
MC3R	-2.96656	9.42E-15
PHF3	-1.27468	5.93E-114
KIAA1586	-1.04222	8.09E-05

FAM83B	-2.47752	1.92E-06
ADNP2	-2.92098	3.49E-179
NFATC1	-1.05626	7.26E-17
ZNF217	-3.13292	1.86E-267
ZNF407	-1.15559	1.60E-19
TIMM21	-1.52619	2.99E-75
SOCS6	-2.40254	4.26E-66
DSEL	-2.02417	2.54E-42
BCL2	-1.49646	0
PHLPP1	-1.20323	5.64E-74
ZCCHC2	-1.61966	4.13E-66
ZFP64	-1.12145	1.56E-16
MALT1	-1.67599	0
NFATC2	-1.17188	2.64E-04
ONECUT2	-1.75769	3.48E-13
RGMB	-2.29292	4.32E-15
CHD1	-1.29731	2.25E-120
MOCS3	-2.17301	3.43E-25
GIN1	-1.55152	8.04E-15
C23H5orf30	-1.00274	1.12E-17
ADNP	-1.99186	3.20E-299
EPB41L4A	-1.19866	1.80E-67
PARD6B	-3.53772	2.87E-56
LOC103244341	-1.33765	5.11E-05
FEM1C	-2.96222	2.51E-114
SEMA6A	-1.68272	0
TNFAIP8	-1.41875	4.69E-43
ZNF608	-2.46055	7.49E-80
CEBPB	-3.65675	0
GRAMD2B	-1.72709	1.13E-26
FNIP1	-1.54709	7.03E-67
IL5	2.355369	8.93E-04
IRF1	-1.49647	1.80E-26
SPATA2	-2.21014	1.82E-55
FSTL4	-1.33651	1.77E-04
JADE2	-1.16744	2.02E-32
B4GALT5	-1.09035	1.32E-76
LOC103244556	-1.03728	1.43E-30
SLC25A48	1.644875	3.56E-04
EGR1	-3.03367	1.54E-154
BRD8	-1.15299	8.92E-110
CXXC5	-1.00873	2.04E-151
IGIP	-1.2533	2.06E-39
TAF7	-1.06817	4.28E-148

NR3C1	-1.29477	5.45E-79
ZMYND8	-1.10213	3.42E-68
RBM27	-1.27138	3.05E-93
FBXO38	-1.08474	5.09E-66
ADRB2	-3.08233	1.72E-195
PCYOX1L	-1.69807	1.56E-26
PPARGC1B	-2.61345	2.97E-179
TIGD6	-2.06053	4.11E-09
CNOT8	-1.07205	1.11E-54
MED7	-1.74678	1.32E-48
TP53RK	-1.52539	4.51E-20
RNF145	-1.79737	1.14E-220
PWWP2A	-1.02282	1.15E-24
CCNJL	-1.1452	5.74E-17
ZBED8	-1.34791	1.49E-11
ZNF334	-1.1941	1.13E-10
LOC103244958	-2.40884	5.01E-06
TLX3	-1.84103	1.25E-04
FGF18	-1.93741	1.83E-04
NEURL1B	-1.13854	1.78E-20
DUSP1	-2.76235	2.96E-251
MSX2	-2.92592	9.91E-21
HK3	2.878931	1.38E-07
UIMC1	-1.02287	1.75E-29
CLK4	-1.69004	7.33E-42
NCOA5	-2.01417	6.20E-118
ZNF354B	-1.0733	9.48E-04
ZNF879	-3.4943	7.97E-24
LOC103245129	-2.29998	1.64E-06
ZFP62	-1.33011	1.34E-43
LOC103245175	-3.22959	5.16E-04
ZSWIM1	-2.29292	4.98E-43
MEX3B	-1.93003	9.63E-04
IREB2	-1.58188	6.35E-187
ZSWIM3	-2.21538	1.02E-17
ISL2	-1.14713	1.62E-06
WFDC3	1.236724	2.74E-04
SIN3A	-1.7165	6.31E-231
IMP3	-1.52756	3.30E-32
SNX33	-1.54275	7.12E-26
C26H15orf39	-2.5455	3.01E-24
SEMA7A	-1.30382	0
PML	-1.29069	1.88E-165
SENP8	-1.57624	8.19E-12

LARP6	-1.18292	1.92E-18
KIF23	-1.03895	1.86E-84
SPESP1	-1.71353	1.06E-23
FEM1B	-2.35532	2.33E-176
PIAS1	-1.19246	4.13E-57
C26H15orf61	-1.44355	1.14E-16
PLEKHO2	-1.75626	5.31E-54
PIF1	-1.00669	1.84E-11
CGNL1	-1.21211	1.52E-52
RFX7	-1.0806	4.55E-93
OSER1	-2.0096	1.79E-107
BLOC1S6	1.864825	0
LCMT2	-1.43051	4.16E-10
ZSCAN29	-1.67631	1.85E-62
TTBK2	-1.00838	2.78E-28
FADD	-1.48593	1.88E-15
SPTBN5	1.026532	1.25E-09
MGA	-1.13006	1.97E-67
CHAC1	-1.94231	2.07E-13
VPS18	-1.66135	1.20E-88
C26H15orf62	-2.32646	8.15E-08
RPUSD2	-2.1757	1.41E-47
CCDC32	-1.31532	1.81E-13
INAFM2	-1.03624	6.79E-15
BMF	-3.0297	0
PGBD4	-2.47752	1.92E-06
FMN1	-1.02281	1.11E-160
NSMCE3	-1.28194	2.71E-51
GABRA5	-1.26912	4.99E-08
FAM83D	-2.61535	1.02E-88
TEC	-1.34765	1.27E-12
DCAF4L1	-1.13006	1.46E-04
LOC103246172	2.293968	3.89E-06
TLR1	-2.45199	5.66E-05
KLF3	-2.22858	6.83E-238
DCAF16	-1.16072	1.03E-27
FBXL5	-1.16204	2.60E-79
CPEB2	-1.04222	8.09E-05
HS3ST1	-2.8155	0
ZNF518B	-2.35641	2.27E-46
ZBTB49	-1.83446	3.33E-15
MSX1	-1.18068	7.40E-09
C27H4orf50	-1.81456	3.43E-30
MRFAP1L1	-1.30608	2.84E-38

BLOC1S4	-1.34285	1.24E-27
KIAA0232	-1.5875	5.47E-96
CCDC96	-2.14713	9.58E-04
TADA2B	-2.05382	1.32E-68
TRMT44	-1.19112	3.65E-16
HAUS3	-1.33211	2.68E-12
JAG1	-1.05967	4.30E-10
LOC103246525	-1.02806	3.96E-11
KCTD7	-1.10268	2.26E-32
LOC103246604	-2.15559	1.30E-05
AUTS2	-1.87217	2.64E-131
CASTOR2	-1.12436	1.20E-28
STX1A	1.076521	9.81E-56
DNAJC30	-1.26122	7.23E-44
VPS37D	-1.16489	1.22E-04
ALKBH4	-1.86483	6.47E-30
UFSP1	-1.52794	1.15E-14
POP7	-1.21036	3.49E-32
BMP2	-2.02194	1.30E-18
MEPCE	-1.568	3.93E-176
ZSCAN21	-1.23508	6.15E-39
ZNF3	-2.26815	1.45E-84
ZSCAN25	-2.01809	2.05E-34
FAM200A	-1.79557	5.62E-20
ZNF394	-2.40082	2.83E-51
USP42	-2.68903	2.46E-157
ZNF12	-2.08757	3.17E-54
FBXL18	-1.46663	5.47E-15
TNRC18	-1.25489	1.40E-81
FOXK1	-1.57112	4.08E-93
TMEM184A	-1.70352	5.79E-04
GPR146	-2.1592	7.36E-14
TMEM230	2.428532	0
ZFY	-1.41838	2.85E-06
NANP	-1.82007	8.74E-18
LOC103247079	2.523019	1.54E-20
ZNF419	-2.29769	2.60E-04
NOA1	1.038933	1.59E-38
BOLA1	-2.12145	3.87E-53
FASTKD5	-1.37468	2.01E-33
CIART	-2.16629	2.09E-31
MCL1	-2.09018	0
GOLPH3L	-1.04386	2.15E-38
CTSK	1.219164	3.38E-10

RPRD2	-1.62102	2.44E-132
BCL9	-1.38138	1.32E-95
NKRF	-2.0297	1.47E-59
RNF113A	-1.28509	3.97E-05
ZBTB33	-1.37169	1.15E-27
ADAM33	1.30353	4.60E-42
CENPB	-1.16411	1.21E-61
LOC103247559	-1.75075	2.59E-10
ZCCHC3	-2.64177	1.27E-102
LOC103247767	-2.40437	1.25E-14
SOX12	-1.53548	2.28E-79
LOC103247796	-1.22959	6.33E-05
LOC103247810	-1.93003	9.63E-04
LOC103247823	-1.46764	1.03E-04
LOC103247851	-1.52548	7.34E-10
TRIM68	-1.46585	1.62E-25
TRIM21	-1.71609	1.18E-32
RHOG	-1.36432	1.20E-70
CHRNA10	5.162724	6.11E-04
ID1	-2.15708	5.82E-32
LOC103248030	-1.1529	1.03E-09
LIPT2	-1.93086	3.21E-13
RNF169	-1.71671	2.49E-61
EMSY	-1.51147	5.19E-41
LRRC32	-1.88655	2.14E-145
TSKU	-2.19156	9.06E-196
B3GNT6	6.262259	2.69E-07
RSF1	-1.22959	1.29E-82
THRSP	-1.87636	1.13E-21
KCTD21	-1.41104	3.43E-30
RAB30	-1.58805	1.30E-11
PCF11	-2.58622	0
LOC103248152	-4.72784	2.10E-06
CREBZF	-1.35558	1.55E-72
LOC103248163	-2.12702	5.80E-16
PLAGL2	-1.61042	8.44E-75
FZD4	-1.45511	1.75E-22
LOC103248202	-1.20406	6.15E-05
ASXL1	-1.83656	1.10E-299
NOL4L	-1.26188	1.47E-19
MED17	-1.18102	1.98E-29
ANKRD49	-1.67949	1.47E-22
FUT4	-1.58949	3.97E-05
KDM4D	-1.14713	1.62E-06

MAML2	-1.38392	5.74E-10
JRKL	-1.64109	1.62E-23
MSANTD4	-1.96271	8.46E-54
RAB39A	-1.71704	6.73E-18
NPAT	-1.60968	9.80E-80
EXPH5	-2.18942	2.39E-84
ZC3H12C	-1.1731	4.81E-40
RBM7	-1.18607	5.50E-77
BUD13	-1.72555	1.23E-59
APOA1	3.525294	4.14E-04
SCN2B	1.746854	6.58E-05
KMT2A	-1.86494	0
PHLDB1	-1.05181	0
DDX6	-1.47027	7.18E-113
BCL9L	-1.79159	0
HINFP	-1.08474	4.26E-14
RNF26	-1.20424	6.82E-159
GRAMD1B	-1.05882	5.88E-17
ZNF202	-1.91644	3.94E-28
HYLS1	-1.45896	2.40E-20
PUS3	-1.43905	2.44E-31
KIRREL3	1.548014	3.20E-05
NCOA6	-1.07809	3.48E-51
KCNJ1	-1.36249	2.59E-06
ADAMTS15	-1.20757	1.38E-05
LOC103248979	-1.57537	3.12E-04
TNFRSF19	-1.43206	2.47E-150
TAF4	-1.79728	2.99E-32
ZNFX1	-1.58973	1.06E-53
RASL11A	-1.03165	9.82E-16

Gene Symbol	log2(SADS-CoV_24h/Control_24h)	Qvalue(SADS-CoV_24h/Control_24h)
UBL3	-1.71576	9.43E-128
USPL1	-1.75725	1.78E-71
MEDAG	-3.03587	7.64E-04
BRCA2	-1.23576	4.99E-30
FRY	-1.03179	2.00E-10
N4BP2L1	-1.36071	2.93E-11
STARD13	-2.25682	0
PDS5B	-1.40333	7.50E-96
N4BP2L2	-1.90079	9.89E-90
SERTM1	-1.47328	4.12E-04
LHFPL6	1.014755	1.76E-05
FOXO1	-3.88275	2.84E-72
ELF1	-1.8485	3.46E-70
KBTBD6	-1.34606	6.23E-09
KBTBD7	-3.25017	2.89E-21
NAA16	-1.33133	4.46E-17
TSC22D1	-2.75479	0
COG3	-2.47328	9.39E-84
ZC3H13	-1.78133	7.99E-82
LRCH1	-1.67714	9.93E-36
HTR2A	-3.07662	3.09E-46
MED4	-2.80751	4.05E-80
ARL11	-1.07913	4.24E-09
KCNRG	-3.84323	6.26E-07
TRIM13	-2.45091	4.60E-17
INTS6	-1.61495	1.77E-46
UTP14C	-1.78144	4.50E-37
ALG11	-1.82917	5.04E-57
THSD1	-2.67825	1.68E-49
FLRT3	-4.65731	0
TDRD3	-1.09257	2.41E-15
PCDH9	-1.92221	5.78E-66
DACH1	-2.27957	2.26E-29
BORA	-1.81938	8.55E-79
KLF5	-3.42525	1.62E-154
KIF16B	-1.2778	6.83E-64
KLF12	-1.18209	5.16E-12
FBXL3	-1.16538	6.56E-39
CLN5	-1.10566	3.89E-64
RNF219	-2.71525	1.09E-45
SPRY2	-5.16858	9.51E-198

LOC103214626	1.58357	8.29E-05
SLITRK5	-2.70824	0
DCT	-2.15135	3.39E-07
ZIC5	-1.28098	2.76E-08
ZIC2	-3.58184	1.11E-114
EFNB2	-3.27109	3.75E-137
LIG4	-1.38532	8.10E-30
ABHD13	-2.27444	1.49E-67
IRS2	-3.79776	1.15E-182
COL4A1	-1.06748	0
ING1	-3.28018	3.24E-97
LOC103214847	-2.62083	5.98E-10
ZNF133	-3.12167	3.16E-51
CHAMP1	-3.35663	2.39E-152
LRRC14B	-4.0055	8.58E-08
CCDC127	-2.14817	4.86E-36
AHRR	-1.62542	5.63E-117
SLC9A3	-1.21246	3.87E-67
SLC6A18	-2.53837	1.44E-07
TERT	1.299113	2.99E-05
LPCAT1	-1.39444	3.34E-42
ICE1	-1.65268	3.97E-112
FASTKD3	-1.6128	7.05E-20
LOC103214994	-1.28569	5.94E-19
ATPSCKMT	-2.17883	2.59E-05
CMBL	5.30184	8.85E-20
ANKRD33B	-2.65444	6.70E-61
OTULINL	-1.42706	4.20E-22
ZNF622	-1.811	3.57E-62
CDH6	-1.57205	0
RIN2	-2.00037	1.51E-40
GOLPH3	-2.30101	0
MTMR12	-1.03262	8.52E-13
NPR3	-1.08077	2.53E-11
DNAJC21	-1.71372	3.39E-150
CFAP61	-1.11064	5.96E-10
NIPBL	-1.05355	8.15E-99
GDNF	-2.28741	1.24E-42
RICTOR	-1.17219	1.14E-63
DAB2	-1.11483	1.45E-194
PTGER4	-3.4051	6.00E-09
PRKAA1	-1.02713	1.58E-22
CARD6	-2.38101	3.32E-50
C4H5orf51	-1.19623	3.60E-83

NIM1K	-2.09861	3.05E-15
ZNF131	-1.47385	1.44E-44
HMGCS1	-4.06096	0
C4H5orf34	-1.40138	6.60E-34
LOC103215245	-2.56192	1.87E-09
ZNF596	-1.6733	1.57E-13
MYOM2	-1.78533	3.52E-15
KBTBD11	-1.90749	1.56E-67
LOC103215277	-1.3578	8.61E-05
KMT5B	-2.4051	1.39E-134
MCPH1	-1.4051	1.47E-27
XKR5	-1.03587	3.87E-04
FDFT1	-1.06389	2.03E-261
PPP1R3B	-4.82703	2.95E-204
CLDN23	-2.51392	4.60E-12
PRAG1	-3.00208	9.46E-25
LONRF1	-2.81466	8.26E-184
MTUS1	-1.00129	7.82E-29
NXT1	-1.97894	1.55E-23
GZF1	-1.08131	5.17E-23
PIWIL2	-1.48653	5.89E-05
C8H8orf58	-3.54087	1.43E-75
TNFRSF10B	-1.34689	2.89E-103
LOC103215470	-1.26921	1.88E-20
TNFRSF10D	-1.21846	5.67E-11
TNFRSF10A	-1.0963	1.40E-21
NKX3-1	-1.26782	3.21E-12
GNRH1	-2.43276	2.96E-08
TRIM35	-1.73586	6.30E-48
KIF13B	-1.03132	1.81E-83
PURG	-2.88831	3.47E-08
FUT10	-1.19595	5.73E-23
TTI2	-1.56737	4.05E-50
RNF122	-2.73631	5.44E-25
UNC5D	-1.18438	4.68E-162
ZNF703	-4.62083	1.28E-06
BRF2	-2.46991	1.05E-49
RAB11FIP1	-1.5582	3.04E-30
BAG4	-1.90251	2.36E-61
PLEKHA2	-1.2516	3.14E-47
ZNF343	-1.18567	1.13E-29
GINS4	1.052938	2.29E-22
KAT6A	-2.7477	3.03E-221
SLC20A2	-1.57487	1.41E-72

THAP1	-1.02076	2.13E-13
STK35	-2.13861	0
DLC1	-2.72071	1.13E-121
LOC103215734	-1.34134	3.95E-12
LOC103215745	-1.27597	4.08E-14
ZNF248	-1.53051	1.06E-49
LOC103215770	-1.16832	3.91E-12
TMEM72	-2.66814	2.03E-10
DEPP1	-6.33637	0
ZNF22	-1.83045	3.71E-36
NCOA4	-1.57853	0
LOC103215829	-1.9705	2.48E-63
FRMPD2	-1.4051	4.04E-05
BMPR1A	-1.4325	2.53E-64
WAPL	-1.89282	1.71E-240
GRID1	-3.4051	4.92E-05
CCSER2	-1.10385	7.68E-50
LOC103215907	-2.15135	3.61E-05
PPIF	1.522374	1.25E-210
ZMIZ1	-1.2058	1.24E-53
FAM110A	-2.32865	1.10E-13
ZNF503	-4.98424	1.60E-115
SAMD8	-1.78675	4.91E-120
KAT6B	-1.88731	9.25E-33
FUT11	-1.37572	3.36E-31
TBC1D20	-1.86826	5.30E-81
DDIT4	-4.56195	0
CHST3	-1.72775	1.90E-08
EIF6	1.138222	2.83E-295
ADAMTS14	1.658716	1.04E-07
TET1	-3.3578	7.31E-05
HNRNPH3	-1.53837	2.67E-283
SIRT1	-3.3283	1.03E-101
JMJD1C	-2.24769	2.63E-199
NRBF2	-2.41174	7.80E-107
ADO	-1.26431	6.26E-53
ARID5B	-4.38091	0
CCDC6	-1.27073	4.75E-94
IPMK	-1.87203	2.53E-77
SPAG4	1.108519	9.19E-05
MTRNR2L5	4.044281	5.45E-240
DKK1	-3.62083	6.78E-06
LOC103216188	-3.99255	2.06E-04
CSTF2T	-2.89913	1.62E-111

A1CF	-3.03587	7.64E-04
KLLN	-2.74859	3.22E-07
PTEN	-1.34688	6.10E-98
FAS	-1.59084	1.20E-37
IFIT2	-4.55943	1.51E-42
IFIT3	-2.11387	8.57E-10
IFIT1	-2.66036	8.26E-05
IFIT5	-2.0027	2.84E-10
SLC16A12	-1.97447	2.55E-04
KIF20B	-1.60555	1.57E-214
HTR7	1.701095	8.79E-08
ANKRD1	-4.05082	6.35E-15
PPP1R3C	-5.21151	7.92E-238
TNKS2	-1.23842	1.99E-94
CPEB3	-1.01162	3.99E-04
KIF11	-1.12442	3.11E-105
HHEX	-4.99007	5.58E-30
LOC103216270	3.08966	5.87E-12
TBC1D12	-1.10533	3.14E-19
LOC103216306	-1.2232	1.98E-04
CCNJ	-1.70964	4.22E-39
ZNF518A	-2.99098	1.49E-176
LCOR	-1.27916	9.80E-50
FRAT1	-3.47328	1.86E-09
FRAT2	-3.1624	1.45E-16
RRP12	1.351758	2.33E-158
CNNM1	3.613222	7.97E-19
ABCC2	-1.2804	1.36E-61
DNMBP	-2.01162	2.66E-84
SCD	-1.43623	0
SLF2	-1.27776	1.84E-99
BTRC	-1.58116	2.17E-95
OGA	-1.55329	0
HPS6	-2.92298	8.39E-76
PPRC1	-2.70263	1.57E-115
WBP1L	-2.35888	6.99E-116
CNNM2	-1.94395	1.24E-62
TAF5	-1.76681	2.15E-15
TGIF2	-1.96302	1.68E-35
SFR1	-1.36026	4.43E-13
ITPRIP	-2.71561	1.16E-18
SORCS3	1.277287	5.01E-10
DUSP5	-2.13129	3.51E-39
RBM20	-1.47081	7.56E-08

PDCD4	-1.42159	4.29E-60
SHOC2	-1.30889	1.40E-47
GPAM	-1.00793	1.80E-75
NRAP	-4.0055	8.58E-08
HABP2	-1.82933	3.54E-190
SOGA1	-2.51578	3.05E-83
CASP7	-2.0089	7.53E-61
ADRB1	-4.66036	2.01E-12
CCDC186	-1.19598	5.54E-17
FAM160B1	-2.85301	3.66E-95
EMX2	-3.24505	5.37E-157
NANOS1	-1.16451	2.49E-41
EIF3A	-1.94963	0
BAG3	-1.84894	1.17E-112
SLC5A3	-1.16057	3.87E-06
IKZF5	-2.03587	3.59E-50
CHST15	-1.08267	3.88E-17
ZRANB1	-1.92807	6.81E-94
SON	-1.80975	0
DOCK1	-1.57249	2.66E-242
PPP2R2D	-1.62219	5.92E-57
LOC103216760	1.342641	8.75E-12
TCF7L2	-2.15662	1.95E-43
DNAJC28	-3.51268	1.38E-22
ABRAXAS2	-2.01384	3.01E-56
IMP4	1.016914	1.56E-80
SAP130	-1.47328	5.22E-87
AMMECR1L	-1.33995	2.05E-61
WDR33	-1.3358	3.50E-130
SFT2D3	-2.40933	2.27E-31
MAP3K2	-2.10451	4.96E-173
NIFK	-1.33955	2.48E-52
GLI2	-1.53027	1.58E-52
PTPN4	-1.04032	5.49E-27
TMEM177	-3.90094	1.78E-127
SCTR	-1.94276	3.51E-04
TMEM37	-1.88473	6.52E-23
INSIG2	-1.42449	2.06E-43
LOC103216964	-3.15135	2.54E-10
LYPD1	1.030511	2.63E-100
SYNJ1	-1.47566	1.58E-49
CCNT2	-1.70217	1.82E-98
ZEB2	-2.1455	1.12E-57
ACVR2A	-1.83535	8.38E-47

MBD5	-1.36231	1.35E-46
EPC2	-2.78114	5.06E-109
RND3	-3.99587	7.56E-210
RIF1	-1.16873	2.29E-66
NR4A2	-3.77284	7.79E-04
TANC1	-1.37337	1.22E-54
SCAF4	-2.14003	4.99E-127
BAZ2B	-1.43625	1.91E-48
7-Mar	-1.992	1.46E-228
ITGB6	-1.14817	3.01E-08
TANK	-1.24243	3.14E-36
FIGN	-1.99364	1.96E-43
SCN2A	-1.43506	6.19E-37
LOC103217237	-4.25362	3.21E-05
NOSTRIN	6.249531	3.66E-07
TLK1	-1.36576	2.39E-71
SLC25A12	-1.48044	5.04E-103
SP3	-1.50597	2.23E-154
GPR155	1.080444	1.04E-32
ATF2	-1.24968	3.38E-84
HOXD13	-1.34834	2.16E-16
HOXD9	-1.50803	9.10E-27
HOXD3	-2.60845	1.31E-23
HOXD4	-2.34609	3.88E-43
NFE2L2	-2.09274	0
LOC103217397	-3.74914	6.82E-33
LOC103217405	-3.25775	1.77E-31
PLEKHA3	-1.50234	2.67E-13
SESTD1	-1.00604	4.65E-43
CWC22	-1.5809	1.43E-78
ITPRID2	-1.07077	6.24E-73
BACH1	-4.4468	6.75E-209
PDE1A	-1.11833	2.31E-04
CALCRL	2.442176	1.45E-04
COL3A1	1.897015	8.19E-04
SLC39A10	-1.0725	0
STK17B	-1.42173	3.69E-227
ANKRD44	-1.88295	1.82E-21
LTN1	-1.05554	5.29E-73
MARS2	-1.62083	5.21E-30
SATB2	-1.01938	7.09E-08
C10H2orf69	-3.82884	4.85E-69
SGO2	-1.13584	7.24E-45
CLK1	-2.56483	8.55E-147

MPP4	5.134054	1.16E-06
FZD7	-2.46471	3.68E-58
NOP58	1.023436	2.35E-183
BMPR2	-1.31738	8.84E-165
FAM117B	-1.5136	2.52E-18
RAPH1	-1.48743	4.88E-16
ADAMTS1	-2.56643	0
INO80D	-2.3609	2.63E-41
GPR1	2.847621	1.49E-09
ZDBF2	-1.3049	1.21E-28
KLF7	-1.61471	2.66E-111
CREB1	-1.37814	2.63E-82
FZD5	-1.82014	2.79E-22
PLEKHM3	-1.04561	4.96E-21
PIKFYVE	-1.87148	2.89E-90
KANSL1L	-1.10073	1.10E-21
IKZF2	-2.28661	1.57E-22
BARD1	-1.0031	8.21E-22
ABCA12	-3.4953	2.24E-05
RUFY4	1.549091	4.76E-05
CTDSP1	-1.06227	6.20E-63
RNF25	-1.7293	7.10E-22
CDK5R2	-2.84323	1.51E-14
CARNS1	-2.59387	2.86E-06
KCNE4	-4.24316	2.45E-97
MRPL44	-1.89442	2.72E-83
CUL3	-1.84559	2.95E-269
IRS1	-2.43797	4.42E-21
HTR2B	-4.17429	1.11E-46
B3GNT7	-3.74246	2.38E-158
TIGD1	-2.2861	3.05E-14
EIF4E2	-1.77916	7.15E-165
GIGYF2	-2.22499	1.68E-155
HJURP	-1.94841	5.90E-92
LOC103218119	-4.56985	1.12E-63
C2H21orf91	-1.40868	1.75E-05
ARL4C	-2.84563	0
SH3BP4	-1.47983	3.56E-68
GBX2	-1.77284	6.02E-06
CXADR	-1.00943	2.04E-70
KLHL30	-1.74369	9.01E-10
ERFE	1.712423	7.73E-30
HES6	1.016596	2.42E-05
PER2	-1.28231	1.20E-05

NRIP1	-2.65198	0
LOC103218231	-2.84803	1.22E-05
MTERF4	-2.42862	2.18E-75
LOC103218293	-2.06562	9.70E-05
HSPA13	-1.57133	2.05E-147
KDM5A	-1.22401	1.01E-49
FBXL14	-2.06434	2.97E-42
DCP1B	-2.09253	1.82E-120
FKBP4	1.232497	0
TULP3	-1.04131	2.23E-71
DYRK4	5.356446	2.28E-04
AKAP3	-1.0992	1.20E-10
PLEKHG6	-1.5516	3.39E-71
TNFRSF1A	-1.5471	0
LPAR5	-2.99007	3.41E-32
LRRC23	-1.06123	4.75E-09
RUNX1	-1.93067	6.29E-130
C1RL	-1.3672	1.39E-32
FOXJ2	-1.27943	1.24E-46
C3AR1	-1.47911	2.54E-08
CLCF1	-1.20973	6.79E-23
CBR3	-1.15135	4.81E-06
MORC3	-2.175	1.45E-125
ETV6	-1.13209	1.66E-12
LRP6	-2.59631	4.99E-152
MANSC1	-1.79561	2.64E-28
BORCS5	-1.01384	8.63E-08
DUSP16	-2.00776	3.19E-158
CDKN1B	-5.01351	0
GPRC5A	-3.24965	3.48E-61
ATF7IP	-2.16737	2.35E-64
PLEKHA5	-1.78773	2.82E-194
DYRK1A	-2.64112	6.98E-183
AEBP2	-1.88504	5.69E-129
PDE3A	-2.22852	6.31E-09
KCNJ8	-2.68607	0
KRAS	-1.72437	3.85E-63
RASSF8	-2.17412	0
PTHLH	-3.9587	6.76E-14
DENND5B	-1.01758	3.45E-27
RESF1	-3.13647	5.31E-46
ETS2	-2.00454	1.52E-106
SYT10	-1.65304	1.10E-220
LOC103218846	-1.01162	1.17E-08

LOC103218871	-1.82942	2.19E-04
SINHCAF	-1.28204	5.93E-30
FGD4	-1.23052	1.00E-04
LOC103218894	-1.8569	1.61E-04
MORN5	-3.09476	5.18E-04
TTLL11	-1.22098	9.82E-08
BRWD1	-1.62708	9.86E-99
CNTRL	-1.75335	1.39E-48
PSMD5	-1.34276	6.69E-41
MEGF9	-3.6805	3.53E-125
BRINP1	-1.3188	2.63E-15
TRIM32	-3.29221	2.90E-157
AKNA	-1.30607	2.40E-30
ZNF618	-1.18282	6.90E-63
RGS3	-1.15014	1.16E-56
HDHD3	-3.33499	1.18E-62
ZFP37	-4.58019	2.95E-22
UGCG	-2.20799	0
LPAR1	-1.41402	1.11E-136
PTPN3	-2.7171	0
ZNF462	-1.15545	2.88E-45
ABCA1	-1.54813	4.57E-23
ZNF189	-3.66814	2.63E-50
MSANTD3	-1.79857	6.20E-63
ALG2	-1.94514	4.88E-134
TGFBR1	-1.37079	4.16E-222
TBC1D2	-1.04877	2.67E-49
SHB	-1.42009	1.24E-208
ZBTB5	-2.2316	2.69E-44
RNF38	-1.10511	3.60E-52
GNE	-1.80463	2.59E-121
HRCT1	-2.80846	2.01E-35
RIPK4	-2.53064	1.39E-18
TPM2	1.352949	1.87E-37
RUSC2	-1.02857	2.48E-57
KDM2A	-1.27763	1.72E-160
FAM214B	-1.48194	6.59E-79
C12H9orf131	-1.2394	8.49E-04
DNAJB5	-1.88991	1.97E-69
CCL27	1.223691	1.49E-09
MYORG	-1.10976	8.27E-22
UBAP2	-1.00871	1.66E-122
NOL6	1.549091	4.32E-227
NFX1	-1.26398	3.91E-74

ZBTB21	-2.05906	5.89E-65
TOPORS	-3.29665	7.43E-172
ACER2	-1.66296	2.32E-16
DENND4C	-1.35071	1.87E-94
RRAGA	-1.89408	1.10E-207
SAXO1	-2.17175	6.49E-29
BNC2	-1.21569	2.67E-27
WDR4	1.30068	7.07E-36
RANBP6	-3.53064	7.98E-192
KIAA2026	-2.18921	5.57E-86
RIC1	-1.28021	4.30E-62
LOC103219486	-1.15135	7.30E-07
CDC37L1	-2.05294	2.84E-44
PLPP6	-3.26004	9.61E-99
VLDLR	-3.45337	2.14E-138
DMRT1	1.616206	1.61E-04
KANK1	-1.03716	1.40E-30
LOC103219534	-1.34021	1.20E-07
PIP5K1B	1.243842	9.55E-05
FAM122A	-3.37541	1.56E-72
SIK1	-4.1284	2.00E-285
SMC5	-1.21226	1.26E-61
TRPM3	-1.47328	1.11E-42
KLF9	-4.55644	2.63E-73
CEMIP2	-1.17929	1.36E-89
ABHD17B	-3.19317	7.55E-55
ZFAND5	-3.06011	0
RORB	-1.71394	7.26E-04
GCNT1	-1.93369	2.04E-30
PRUNE2	-1.23646	3.45E-97
PSAT1	1.149359	0
TLE4	-1.04564	2.60E-04
TLE1	-1.38873	5.10E-187
KIF27	-1.15135	3.08E-18
RMI1	-1.91558	1.05E-37
TRAPPC10	-1.17891	5.23E-72
TUT7	-1.43619	1.40E-69
GAS1	-8.41958	2.63E-135
C1H11orf86	-1.74859	1.32E-04
LOC103219727	-1.59557	3.61E-59
NFIL3	-4.38919	1.34E-226
IPPK	-1.03587	6.98E-13
LOC103219782	-1.36856	3.70E-37
ZNF484	-3.39577	2.12E-20

ZNF658	-2.43411	2.72E-39
CDC14B	-1.15427	1.53E-70
ZNF367	-2.5494	3.01E-38
PTCH1	-3.25826	7.57E-35
AOPEP	-1.5327	2.09E-56
ZNF782	-1.06141	7.49E-06
KDM3A	-1.86574	2.86E-147
DQX1	-1.07674	3.43E-06
DOK1	-1.31181	1.49E-29
PCGF1	-1.12076	9.26E-14
C14H2orf81	-1.66036	3.45E-05
TET3	-1.73355	8.26E-39
LOC103220050	-3.84323	5.20E-04
EXOC6B	-1.00053	4.97E-72
CYP26B1	2.759127	1.26E-47
ZNF638	-1.23833	5.53E-99
LOC103220073	-1.12898	6.11E-09
MPHOSPH10	-2.41408	1.66E-86
C14H2orf42	-1.37094	5.00E-17
PCBP1	-1.33851	0
MXD1	-3.17527	1.71E-72
GMCL1	-1.02976	8.57E-32
AAK1	-1.03756	1.14E-23
FBXO48	-1.95341	3.92E-08
ETAA1	-2.57711	2.41E-112
LOC103220141	-3.09476	5.18E-04
MEIS1	-1.52986	2.28E-09
SPRED2	-2.21953	9.19E-196
CEP68	-1.36207	1.20E-28
SERTAD2	-4.06005	0
LOC103220160	-3.30889	1.08E-04
AFTPH	-2.16319	1.30E-114
PELI1	-1.87116	5.56E-211
LOC103220168	-2.19578	2.97E-09
OTX1	-5.12333	5.80E-33
TMEM17	1.167457	1.82E-14
B3GNT2	-2.48908	2.84E-115
REL	-3.53837	1.00E-18
PAPOLG	-2.47678	1.76E-57
BCL11A	-2.53837	3.58E-09
LOC103220237	-1.81693	1.20E-19
ERLEC1	-1.20515	3.33E-132
STON1	-2.00416	5.29E-58
FOXN2	-1.84176	3.89E-32

MSH6	-1.90731	1.66E-157
FBXO11	-1.39436	1.49E-117
SOCS5	-2.55677	2.21E-90
PRKCE	1.372665	5.44E-16
SIX2	-2.73631	3.94E-05
SIX3	-1.99523	4.50E-06
PPM1B	-1.19789	3.32E-111
C1GALT1C1L	-3.02456	5.97E-44
ZFP36L2	-4.39037	1.98E-73
PKDCC	-1.14845	2.30E-24
C14H2orf91	-1.54607	8.73E-06
THUMPD2	-1.05611	5.99E-11
MAP4K3	-1.85783	7.83E-80
GEMIN6	-2.02878	1.03E-15
ATL2	-1.14398	2.45E-58
CYP1B1	-2.0143	4.11E-62
CDC42EP3	-1.59832	1.26E-206
PRKD3	-1.49875	1.43E-118
CEBPZ	-1.47782	2.79E-90
HEATR5B	-1.43777	2.23E-68
BIRC6	-1.45257	1.24E-194
YPEL5	-1.65053	1.68E-93
WDR43	-1.93348	0
FOSL2	-3.33493	0
CCDC121	-1.12898	4.84E-04
SLC5A6	1.389692	1.54E-79
PREB	1.017716	6.00E-85
DPYSL5	1.333805	7.91E-116
OTOF	-1.23089	2.92E-10
DRC1	-1.17753	1.15E-05
SELENOI	-1.24853	5.00E-43
DNAJC27	-2.38872	1.18E-54
WDCP	-2.16269	9.92E-41
ATAD2B	-1.74831	3.08E-52
GDF7	-1.82014	2.79E-22
RHOB	-5.01705	0
PUM2	-2.6905	0
OSR1	-4.62083	1.28E-06
RDH14	-2.12333	6.53E-36
LRATD1	-2.86173	3.75E-100
TRIB2	-4.2257	5.68E-96
LPIN1	-1.16178	0
E2F6	-1.97541	2.92E-54
GREB1	1.205842	3.66E-15

ODC1	1.030127	0
LOC103220823	-2.23227	1.32E-05
KLF11	-2.69995	2.19E-130
LOC103220837	2.25536	1.32E-08
ID2	-4.76791	1.22E-134
SOX11	-2.00111	6.08E-07
RBM39	-1.66451	3.33E-176
ALKAL2	-2.81877	5.93E-38
SH3YL1	-1.13896	7.00E-12
LOC103221001	-3.88387	4.13E-13
ASXL2	-1.08347	6.11E-56
CSRNP1	-3.57519	4.48E-150
ACVR2B	-1.92304	3.12E-107
SLC22A14	1.812126	2.19E-04
GOLGA4	-1.04773	5.29E-99
EPM2AIP1	-2.18819	2.70E-119
TRANK1	-1.47328	4.12E-04
TBCCD1	-2.15604	6.63E-119
MAGEF1	-1.31118	2.88E-37
LINC00205	-1.23256	1.82E-22
B3GNT5	-3.19115	1.84E-70
KLHL24	-4.08235	2.39E-50
SOX2	4.008523	1.08E-05
RBM4	-1.41949	6.61E-122
ZMAT3	-2.49998	2.51E-86
TBL1XR1	-1.40971	1.71E-111
LOC103221232	1.674747	5.40E-08
NLGN1	-1.08562	1.27E-44
FNDC3B	-1.63993	0
PLD1	-1.33952	5.36E-43
SLC2A2	-1.59387	1.28E-06
SLC7A14	6.639945	6.36E-09
SKIL	-3.42016	0
PHC3	-1.09592	3.51E-86
PELO	-4.3591	0
LRRC31	-1.05052	3.43E-09
MYNN	-1.6324	2.26E-34
MECOM	-1.38709	1.65E-09
ZNF639	-3.24792	9.99E-68
FST	-1.71394	1.21E-06
GCLC	-1.9796	8.81E-225
GCM1	-1.91034	4.81E-04
PAQR8	-2.05011	5.34E-38
PKHD1	-1.11387	6.13E-08

SNX18	-2.00703	2.67E-67
ADGRF2	-1.28203	1.59E-12
ADGRF4	-1.31705	2.16E-23
ADGRF5	-1.87237	5.37E-06
RUNX2	-1.32596	1.95E-33
LOC103221425	-2.49044	8.60E-15
RBM4B	-2.86985	1.91E-64
MAD2L1BP	-2.73759	1.23E-58
POLH	-1.3578	4.83E-70
ZNF318	-1.29928	6.36E-59
BICRAL	-2.89298	7.75E-90
TBCC	-3.17402	7.37E-54
TFEB	-1.27688	8.40E-10
NFYA	-1.22716	4.36E-69
SAYSD1	-1.46373	2.71E-06
TMEM217	-2.43221	3.00E-08
TBC1D22B	-2.02737	6.15E-98
PIM1	-3.60172	8.56E-177
CDKN1A	-1.55272	0
BRPF3	-1.61831	7.91E-91
IP6K3	-2.42099	3.97E-94
ZBTB9	-3.61582	4.11E-76
KIFC1	-1.32205	3.29E-68
ZBTB22	-1.40843	2.81E-38
B3GALT4	-1.09137	2.62E-10
MHC-DOA	5.106134	1.50E-06
MAP3K1	-4.27382	0
PBX2	-1.01992	3.45E-86
FKBPL	-3.53365	2.29E-40
C4B	-1.4705	4.46E-06
MIER3	-3.2682	2.75E-106
ZBTB12	-1.46062	1.19E-14
HSPA1B	-4.33363	0
HSPA1L	-1.87717	6.57E-04
HSPA1A	-3.55976	2.97E-151
LY6G5B	1.019982	4.12E-07
LOC103221768	-1.17619	8.24E-08
MUCL3	-2.4051	7.22E-04
IER3	-3.60014	0
PLK2	-3.78119	0
PPP1R10	-4.47881	0
PRR3	-1.0025	1.16E-16
LOC103221842	-1.28231	1.20E-05
TRIM26	-2.15662	1.95E-43

MOG	3.997992	1.18E-14
PDE4D	-2.87759	0
ZBED9	-1.72775	1.90E-08
ZKSCAN3	-1.20338	1.68E-04
PGBD1	-1.13103	1.92E-05
ZSCAN26	-1.52835	4.69E-15
ZKSCAN4	-1.39844	9.79E-06
ZNF165	-4.62083	1.28E-06
ZSCAN16	-5.25826	5.51E-10
ZKSCAN8	-1.78589	3.73E-81
CCS	1.165176	7.86E-09
HIST1H1B	5.134054	7.11E-04
LOC103221946	-1.61237	4.52E-10
LOC103221947	-2.8644	1.85E-14
ZNF391	-2.65254	2.92E-10
ZNF184	-2.4599	8.93E-16
ZNF322	-2.83585	9.22E-63
ABT1	-2.10371	1.78E-43
LOC103222011	-1.97626	2.17E-84
LOC103222014	-2.27655	6.25E-34
LOC103222020	-1.25826	4.87E-17
SLC17A2	-2.03587	1.34E-04
C17H6orf62	-1.22827	2.66E-114
TDP2	-1.02245	1.01E-17
DCDC2	-1.07315	3.94E-87
ZSWIM6	-2.32538	1.72E-75
E2F3	-4.0106	1.88E-80
KIF13A	-2.17378	1.14E-56
NUP153	-2.46035	1.30E-291
MYLIP	-3.09476	7.95E-10
KIF2A	-1.15304	2.94E-94
JARID2	-2.00964	6.21E-32
EDN1	-4.09476	4.97E-235
HIVEP1	-3.49239	1.07E-126
NEDD9	-4.15135	3.01E-53
TFAP2A	-2.75146	8.99E-138
SNRNP48	-2.02281	1.77E-32
RREB1	-2.26179	1.76E-302
PPP1R3G	-1.58669	3.19E-18
PRPF4B	-1.918	0
FAM50B	-1.41343	2.91E-14
PXDC1	-2.0028	8.27E-95
SLC22A23	-1.32051	6.62E-47
FOXC1	-2.45451	2.52E-37

FOXF2	-3.15303	3.02E-101
FOXQ1	-5.60066	1.29E-23
IRF4	-3.53837	1.50E-05
PPWD1	-1.47498	7.76E-40
TAF8	-1.47081	1.78E-04
LOC103222346	-1.47197	1.73E-07
LOC103222352	11.1235	6.45E-134
TRIM23	-1.56795	5.27E-32
ZSCAN12	-3.10579	4.55E-19
LOC103222374	-2.32069	6.03E-53
LOC103222379	-4.03587	5.76E-08
MEX3C	-1.28054	2.13E-68
ERBIN	-1.59415	1.42E-132
SMAD4	-1.32861	7.09E-69
LIPG	-2.5213	1.23E-24
SMAD7	-5.08511	4.96E-186
RNF165	1.881667	1.25E-07
EPG5	-1.44865	1.20E-114
SETBP1	-1.18362	7.68E-05
PIK3C3	-1.23041	2.61E-155
MAST4	-2.13541	1.21E-69
CELF4	-1.41027	3.88E-04
ZNF24	-1.70041	1.24E-129
ZNF397	-1.8536	2.59E-23
ASXL3	-2.7952	6.03E-35
GAREM1	-1.48567	3.05E-32
KLHL14	-3.4358	3.60E-13
RNF138	-2.62083	7.58E-38
TRAPPC8	-1.32563	2.47E-74
PIK3R1	-4.63714	0
KCTD1	-1.40209	1.11E-46
ZNF521	-1.92123	4.70E-10
HRH4	-1.02694	2.45E-05
CABYR	-1.57987	4.79E-115
GATA6	-3.52416	3.50E-14
ESCO1	-1.40774	3.32E-71
EMILIN2	-2.53837	3.58E-09
TGIF1	-3.36374	0
LOC103222706	-4.04337	1.99E-28
DLGAP1	1.134054	4.83E-14
ZBTB14	-1.23298	6.60E-19
ARHGAP28	-1.23518	1.11E-07
ANKRD12	-1.5769	8.26E-73
CDK7	-2.09975	1.01E-119

CHMP1B	-1.71148	5.43E-173
CCNB1	-1.02581	2.79E-185
CEP76	-1.54728	3.62E-17
FAM210A	-1.35224	2.42E-32
IL17RA	-1.02535	1.56E-57
TMEM121B	-1.87717	6.57E-04
BDP1	-1.04288	1.43E-28
CECR2	-1.04799	4.41E-07
USP18	1.146993	2.92E-06
LOC103222906	-1.82595	2.46E-06
YDJC	1.34416	2.49E-89
HIC2	-1.86941	4.97E-19
CRKL	-1.9063	1.84E-141
ZNF74	-1.11387	2.26E-07
RANBP1	1.020783	0
CDC45	1.466319	8.40E-138
TMEM171	-1.40732	3.78E-08
TSSK2	-1.78878	1.12E-06
ESS2	-1.3739	1.18E-32
ZNF70	-2.06562	2.29E-08
FOXD1	-2.38241	2.42E-42
PHRF1	-1.55301	1.11E-115
ADORA2A	1.022771	3.45E-13
LOC103223066	-1.09701	4.03E-15
ARHGEF28	-1.41868	6.15E-224
ASPHD2	-2.16515	2.35E-09
TFIP11	-1.16383	8.40E-51
SRRD	-1.35539	2.59E-25
MN1	-2.59152	5.53E-99
TTC28	-1.31863	5.52E-63
ENC1	-2.64502	2.07E-42
CCDC117	-1.83142	4.02E-62
XBP1	-2.75542	8.45E-304
ZNRF3	-1.44446	1.43E-76
NSA2	-1.4622	0
MTMR3	-1.19664	5.13E-83
LIF	-1.2076	1.14E-175
CASTOR1	-4.03587	2.94E-28
TBC1D10A	-1.82266	1.56E-48
GAL3ST1	-2.55105	1.73E-33
DUSP18	-1.2838	9.05E-12
PIK3IP1	-1.91034	8.69E-05
PATZ1	-1.75347	3.75E-79
SFI1	1.336895	6.32E-34

GCNT4	-3.25826	1.60E-04
EIF4ENIF1	-3.31951	2.34E-250
PRR14L	-2.51121	1.17E-253
DEPDC5	-2.02234	1.47E-106
HMGXB4	-2.3503	5.50E-116
HMOX1	-1.42313	2.87E-61
MCM5	1.304423	0
HMGCR	-3.11589	0
NCF4	1.154813	2.34E-04
C1QTNF6	-1.98073	3.33E-17
SSTR3	-1.49044	1.36E-04
COL4A3BP	-1.38222	5.60E-53
ELFN2	-1.31397	6.20E-72
CDC42EP1	-1.01911	7.35E-87
PDXP	1.350947	2.52E-43
H1F0	-1.59365	0
PDGFB	-3.02842	0
F2RL2	-1.27688	6.45E-07
ATF4	-1.13276	0
CACNA1I	-3.60066	1.78E-10
MRTFA	-1.26617	3.96E-54
F2RL1	-2.12761	9.27E-193
EP300	-2.55889	0
CHADL	2.018577	6.74E-05
TEF	-2.64918	2.62E-177
TOB2	-4.40421	1.88E-238
TCF20	-2.68847	1.01E-235
LOC103223421	1.549091	2.43E-05
EFCAB6	-1.83697	2.12E-08
RTL6	-2.51568	1.20E-228
NUP50	-1.55488	3.82E-118
BRD1	-2.09888	1.29E-105
PIM3	-1.02245	1.01E-17
ZBED4	-4.04896	2.58E-110
MAPK11	1.05411	4.14E-52
LHFPL2	-1.2672	2.83E-42
TYMP	1.330911	8.41E-07
JMY	-1.7235	2.89E-65
TNRC6B	-2.01676	6.08E-49
HSPA6	-4.38165	3.38E-19
MPZ	-1.58841	6.25E-08
TENT2	-1.42413	5.86E-149
USP21	-1.22888	8.47E-40
PFDN2	1.077559	2.05E-52

PIGM	-2.59139	8.14E-20
MTX3	-1.62635	2.27E-56
TMEM151A	-1.93204	3.46E-10
SERINC5	-1.14064	8.10E-22
ETV3	-3.13134	7.78E-126
PRCC	-2.27168	6.48E-152
PAQR6	1.381981	4.06E-08
TMEM79	-1.56639	6.49E-13
ANKRD34B	-1.67477	3.30E-19
MEF2D	-1.78232	4.56E-56
MEX3A	-1.58947	3.72E-15
KHDC4	-1.64704	2.23E-99
RIT1	-1.34107	3.42E-40
SYT11	-2.66036	8.26E-05
ASH1L	-1.34387	6.23E-136
EFNA1	-2.8536	4.30E-75
EFNA3	-1.62655	1.43E-14
ZBTB7B	-1.22427	2.00E-54
EFNA4	-2.7194	1.28E-22
CHRNB2	-1.38951	5.02E-04
IL6R	-1.89596	2.91E-40
LOC103223928	-2.13941	8.82E-29
UBAP2L	-1.57385	4.53E-283
GATAD2B	-1.50257	3.34E-57
CHTOP	-1.35143	1.76E-64
LOC103223983	2.499314	8.03E-04
LOC103224002	2.87102	2.84E-04
RORC	-2.44269	6.50E-17
TUFT1	-1.08818	4.50E-88
POGZ	-2.25546	2.60E-210
ZNF687	-2.3761	1.22E-186
LYSMD1	-1.78589	2.93E-21
TXNIP	-4.82584	0
ANKRD34A	-3.4953	1.26E-09
PEX11B	-2.31105	2.97E-29
NUDT17	-1.11721	6.14E-05
PDZK1	-2.03514	1.21E-142
CD160	-1.53837	1.46E-37
NBPF8	-1.7916	9.61E-40
ZNF697	-3.72703	6.00E-22
TBX15	-3.33355	1.93E-08
VCAN	-1.9772	0
CD58	-1.12661	1.72E-19
VANGL1	-1.81395	5.73E-82

NGF	-2.61479	2.73E-231
DENND2C	-1.84323	8.83E-12
TRIM33	-1.47758	4.21E-76
RSBN1	-4.2964	9.00E-152
KLC2	1.037752	2.58E-73
LRIG2	-2.52332	2.53E-54
CTTNBP2NL	-2.22174	5.41E-132
DDX20	-2.83995	6.92E-94
RASA1	-1.05801	2.51E-97
WDR77	1.067612	1.33E-89
LRIF1	-3.03172	3.70E-79
STRIP1	-1.40792	7.68E-45
AMIGO1	-1.91034	9.94E-08
ATXN7L2	-2.82595	1.15E-18
GPSM2	-1.39714	2.26E-54
PRPF38B	-1.75977	5.95E-137
FAM102B	-1.62404	5.99E-25
VAV3	-2.79988	1.06E-157
PRMT6	-1.55741	1.88E-14
COL11A1	-1.04474	3.09E-105
S1PR1	-2.09032	3.04E-13
SLC30A7	-1.52381	2.89E-67
DPH5	1.008523	7.37E-24
SASS6	-2.18926	7.50E-83
MFSD14A	-2.56537	5.19E-250
PTBP2	-1.16535	4.98E-16
RWDD3	-1.0812	2.09E-09
CNN3	-1.1003	3.62E-216
F3	-1.88735	2.23E-17
ARHGAP29	-1.79673	0
DNTTIP2	-1.20111	2.93E-75
MTF2	-2.07963	7.38E-85
DIPK1A	-2.41638	2.72E-52
EVI5	-1.37496	1.08E-37
BTBD8	-1.571	6.33E-17
TGFBR3	-1.33005	2.28E-25
HFM1	-1.38045	4.39E-32
ZNF644	-1.95056	1.25E-100
LRRC8D	-1.0813	6.68E-53
LRRC8C	-1.26992	4.28E-36
LYSMD3	-2.91531	9.69E-190
RBMXL1	-2.72992	6.37E-87
GTF2B	-1.73119	6.50E-70
LOC103224490	-3.22701	1.47E-17

LMO4	-1.6163	3.45E-117
MBLAC2	-1.18037	5.23E-24
ZNHIT6	-2.00612	1.12E-41
CCN1	-2.07052	0
BCL10	-2.65354	5.66E-115
C20H1orf52	-1.54222	1.56E-19
SYDE2	-1.8193	3.09E-36
RPF1	-1.97444	1.66E-17
ADGRL2	-1.77863	0
LOC103224561	-2.38951	3.45E-05
DNAJB4	-1.19248	3.35E-56
RABGGTB	-1.24066	6.37E-27
ARRDC3	-4.5116	0
ERICH3	-1.19733	1.13E-09
ANKRD13C	-1.17913	1.53E-98
HHLA3	-3.03587	7.64E-04
LRRC7	-1.32725	3.11E-11
RPE65	-2.29891	3.32E-07
GADD45A	-1.65505	2.27E-63
PDE4B	-5.02582	1.17E-89
LEPR	-1.04475	6.77E-10
NR2F1	-2.61078	4.82E-15
ROR1	-1.65419	5.44E-39
JUN	-5.4206	0
PLPP3	-1.19971	1.61E-40
PARS2	-2.94029	6.47E-65
ACOT11	1.408229	7.96E-16
CPT2	-1.89978	1.11E-116
TUT4	-1.41427	2.22E-109
KTI12	-2.7831	6.80E-45
CDKN2C	-1.03152	4.24E-27
DMRTA2	6.797019	9.49E-10
BEND5	-1.43276	4.04E-08
TRABD2B	-1.86236	0
LOC103224857	-1.41077	7.23E-15
FOXD2	-3.57006	2.92E-21
LURAP1	-3.45091	3.31E-05
PIK3R3	-2.08995	1.38E-67
CCDC17	1.342641	3.65E-04
MMACHC	-1.05357	4.53E-10
TOE1	-1.44104	1.62E-20
EIF2B3	1.124	8.35E-39
PTCH2	1.627094	4.45E-04
RNF220	-1.00245	2.11E-58

KDM4A	-1.10806	3.05E-83
ZNF691	-1.7528	5.41E-18
RIMKLA	1.92501	2.83E-59
FOXJ3	-1.36672	5.28E-54
EDN2	-2.58841	1.23E-09
FOXO6	-1.42888	1.47E-12
CTPS1	1.165698	0
LOC103225021	-2.4953	3.53E-04
LNPEP	-1.42572	4.81E-134
ZNF684	-2.39342	5.40E-12
ZFP69	-1.41311	6.57E-11
RLF	-4.03336	1.79E-243
MFSD2A	1.417302	1.02E-15
MYCL	-1.57744	8.22E-08
LOC103225049	1.09908	1.69E-04
KIAA0754	-1.9974	4.96E-187
FHL3	-1.10534	3.52E-24
MTF1	-2.39902	2.08E-63
LOC103225095	-4.08096	1.23E-33
ZMYM1	-1.02864	1.02E-16
GJB4	-1.50123	1.12E-32
GJB3	-1.36535	3.82E-106
GJB5	-1.11387	4.26E-05
C20H1orf94	2.218943	2.11E-09
ZSCAN20	-2.5116	6.40E-26
ZNF362	-1.27034	9.05E-32
TRIM62	-2.60741	1.51E-11
KIAA1522	-1.1995	0
SYNC	-1.26484	9.82E-33
IQCC	-2.86369	6.75E-24
COL16A1	1.175874	4.01E-11
NKAIN1	1.328215	3.61E-63
PUM1	-3.4277	0
SDC3	-1.31695	2.49E-180
SRSF4	-1.22584	1.19E-138
YTHDF2	-3.1023	0
GMEB1	-1.50455	3.98E-14
RAB42	-1.87717	6.57E-04
PHACTR4	-1.7772	2.69E-139
SESN2	-2.73252	0
RPA2	1.136753	3.41E-144
PPP1R8	-2.62818	5.86E-154
FAM76A	-1.48486	3.74E-09
AHDC1	-2.23227	1.25E-164

TENT5B	-3.32303	2.80E-26
LOC103225309	-1.5213	2.70E-15
ARID1A	-2.2512	0
PDIK1L	-4.02582	2.43E-21
PAQR7	-1.70847	6.01E-31
MACO1	-1.60214	3.19E-76
SRRM1	-1.2891	1.83E-150
IFNLR1	-1.38502	1.26E-05
PNRC2	-4.05511	0
ID3	-3.2655	3.18E-241
ZNF436	-1.90176	5.26E-67
LUZP1	-2.47555	7.38E-189
EPHA8	-2.53837	5.87E-06
C1H11orf68	-1.89604	1.09E-92
MUL1	-2.49132	1.60E-100
FAM43B	-2.87717	8.31E-29
OTUD3	1.99714	8.76E-223
IFFO2	-2.16712	6.24E-101
KLHDC7A	-3.54172	1.72E-167
ARHGEF10L	-1.83118	1.04E-82
FBXO42	-2.5582	1.84E-56
CPLANE2	1.090604	7.69E-13
RHOT2	1.382094	4.39E-147
FAM131C	1.760596	5.81E-05
SPEN	-3.45458	0
DDI2	-1.7786	3.05E-16
RSC1A1	-1.06234	8.89E-18
DNAJC16	-1.06159	4.41E-27
FOSL1	-1.89019	9.28E-43
PRDM2	-1.81481	1.46E-67
DHRS3	-1.30373	4.20E-17
RHBDL1	1.818278	1.07E-09
CLCN6	-1.44352	1.00E-95
MTHFR	-1.15855	1.07E-51
UBIAD1	-2.72889	1.79E-40
SRM	1.166844	2.95E-249
MASP2	-1.58607	9.72E-14
PEX14	-1.19985	2.06E-37
SPSB1	-3.44445	2.72E-29
ERRFI1	-2.57981	3.25E-179
PER3	-1.84508	1.85E-74
THAP3	-1.22794	1.93E-26
PHF13	-3.2838	1.41E-152
KLHL21	-2.04354	1.42E-109

NOL9	-1.00918	4.88E-16
TNFRSF25	1.237147	4.15E-04
CHTF18	1.182122	3.99E-46
C20H1orf174	-2.29221	6.12E-37
SOX8	1.760596	5.81E-05
SKI	-2.95112	3.87E-162
ANKRD65	-2.04001	2.58E-126
CPTP	-1.04253	1.70E-18
DVL1	1.043228	1.55E-114
LOC103225820	-3.94276	3.30E-26
SAMD11	-1.5262	7.26E-07
NOC2L	1.119353	2.28E-213
PLEKHN1	1.154331	1.90E-09
VSIG8	2.356446	1.80E-06
LOC103225896	-2.62563	1.87E-06
STIL	-1.73483	2.18E-57
MACF1	-1.50789	0
LOC103225945	-4.29445	3.12E-71
ZMYM6	-1.72441	3.58E-58
RNF19B	-1.63185	4.48E-29
LOC103225955	-1.35928	6.06E-21
UBE4B	-1.46085	2.47E-171
ATAD3A	1.634387	5.43E-201
LOC103226004	-3.84323	2.58E-41
ZNF273	-4.0123	9.35E-61
ZNF107	-2.43276	2.52E-15
ZNF680	-2.28185	1.58E-16
ZNF713	-1.09727	2.53E-05
FIGNL1	-1.79463	1.73E-29
UPP1	1.389354	8.85E-167
TNS3	-1.3249	0
PURB	-1.98758	2.57E-165
LOC103226149	-1.2859	5.12E-87
HECW1	-1.74553	2.27E-10
LOC103226163	-2.0513	1.48E-55
INHBA	-1.82216	0
CRAMP1	-1.21995	4.25E-30
CDK13	-2.12685	0
ELMO1	1.549091	7.17E-04
KIAA0895	-1.44975	1.16E-111
EEPD1	-1.29011	2.66E-18
HERPUD2	-1.05703	9.66E-87
TBX20	-1.13741	2.69E-04
KBTBD2	-1.95878	6.32E-166

AVL9	-2.33034	1.02E-247
EME2	1.050286	8.94E-05
HOXA13	-3.58019	1.01E-05
HOXA1	-2.07769	1.11E-16
HOXA2	-1.00672	1.23E-19
HOXA3	-3.28018	3.10E-73
HOXA4	-1.29418	9.48E-06
HOXA5	-4.10146	2.69E-46
HOXA6	-2.10817	9.89E-22
HOXA7	-1.93204	1.16E-05
HOXA9	-1.78342	1.49E-99
HOXA10	-2.23968	3.54E-23
FAHD1	-1.11679	4.44E-06
CCDC126	-1.20894	4.88E-05
IL6	-1.21015	1.30E-196
SP4	-2.33618	4.72E-104
SP8	-3.5098	5.16E-14
ITGB8	-2.06472	5.92E-45
AHR	-2.33395	1.63E-77
SNX32	1.076025	3.30E-05
TBL3	1.012455	2.51E-89
ARL4A	-7.04097	2.34E-30
GLCCI1	-1.69037	2.46E-34
MIOS	-1.15464	2.16E-83
C1GALT1	-1.02467	1.16E-60
DLX5	-2.56639	5.14E-16
DLX6	-4.25826	1.31E-25
RBM48	-4.15135	1.14E-60
CYP51A1	-1.37707	0
MTERF1	-3.2058	9.34E-43
FZD1	-2.42374	2.00E-94
CLDN12	-1.23994	2.11E-61
DBF4	-2.44107	1.50E-66
PCLO	-1.59977	1.04E-32
CASKIN1	1.729871	1.84E-07
RSBN1L	-3.52772	7.67E-36
TMEM60	-2.45091	2.66E-11
BRICD5	3.549091	1.05E-05
NAPEPLD	-1.37222	6.88E-24
KMT2E	-3.38684	0
CCDC71L	-3.0865	3.79E-21
HBP1	-2.88734	4.28E-87
CBLL1	-2.31922	3.22E-60
PNPLA8	-1.19204	7.52E-28

DNAJB9	-2.34707	1.42E-35
THAP5	-2.02641	1.76E-44
LRRN3	-2.6901	1.70E-230
TMEM168	-1.20374	1.16E-24
BMT2	-1.6043	3.61E-19
TEDC2	1.154813	7.27E-27
CTTNBP2	-2.91667	2.26E-149
LOC103226788	-1.22578	5.73E-04
ING3	-1.14021	3.99E-05
CADPS2	-1.07769	8.33E-22
WASL	-1.3201	2.82E-57
GPR37	-1.55044	4.26E-04
ZNF800	-2.66296	1.20E-28
GCC1	-2.22123	4.85E-35
HILPDA	-3.58464	1.50E-169
LRGUK	-1.24023	9.14E-07
LOC103226932	1.017944	0
CYREN	-1.90408	1.93E-46
TMEM140	-1.58705	1.06E-22
CNOT4	-1.1939	5.21E-46
LOC103226962	1.031379	1.71E-59
KAT5	-3.9678	7.88E-203
TRIM24	-2.28504	0
KIAA1549	-1.29553	1.26E-74
ZC3HAV1	-2.32659	5.43E-202
PKMYT1	1.296927	2.69E-80
KREMEN2	-2.4051	7.22E-04
TMEM139	-1.39975	3.98E-21
TCAF2	-2.03587	1.96E-18
TCAF1	-1.17024	2.36E-83
ARHGEF5	-1.23244	3.57E-72
LOC103227159	-4.09476	1.03E-04
LOC103227175	-1.9217	3.57E-18
ZNF786	-2.77284	7.94E-22
ZNF398	-2.85818	3.35E-73
ZNF282	-1.0798	1.43E-39
ZNF212	-3.39577	1.00E-77
LOC103227190	-3.4051	4.92E-05
LOC103227191	-4.15135	6.93E-05
ZNF777	-1.35283	2.78E-30
ZNF205	-1.3199	1.54E-21
ZNF746	-2.25458	8.04E-65
ZNF862	-1.4316	1.27E-33
ZNF775	-2.47932	6.36E-34

KMT2C	-1.39532	6.46E-123
HTR5A	-1.2058	1.27E-06
INSIG1	-3.63424	0
RBM33	-1.09257	1.43E-57
ZNF200	-2.35545	1.97E-106
MNX1	-1.08454	3.63E-07
ZNF263	-2.34063	2.19E-79
WDR60	-1.04272	5.04E-18
NPC1L1	-1.08013	7.82E-20
LOC103227384	-1.52957	7.53E-25
LOC103227406	-2.10802	1.60E-13
TIGD7	-2.62083	2.10E-27
EIF1B	-2.12229	5.54E-105
ZNF619	-1.48679	1.84E-06
ZNF621	-1.51392	9.47E-07
LOC103227445	-2.40074	6.78E-04
ZNF174	-1.93769	1.54E-15
SS18L2	-1.33323	1.56E-44
ZSCAN32	-1.11572	6.50E-09
SNRK	-2.25335	7.19E-49
ABHD5	-1.00068	2.47E-37
ZNF197	-3.03186	1.39E-41
ZNF597	-4.0055	8.58E-08
ZNF35	-2.19167	3.20E-21
KIAA1143	-1.55044	7.84E-25
TMEM158	1.989664	4.91E-05
SLX4	-1.59109	2.73E-23
CCDC12	-2.24476	7.53E-53
SETD2	-2.85014	2.83E-244
CCDC51	-1.07302	6.51E-21
TREX1	-1.83901	2.54E-33
UCN2	1.045049	1.85E-05
IP6K2	-1.75393	7.90E-99
WDR6	-1.375	1.25E-291
QRICH1	-2.00077	1.19E-256
GLIS2	-2.96187	2.10E-151
CCDC71	-2.70182	6.43E-55
VASN	-3.15792	0
BSN	-1.12584	1.60E-31
TRAIP	1.12407	3.55E-20
HMOX2	-1.1645	3.81E-36
RBM5	-1.75932	5.95E-226
IFRD2	1.291766	1.39E-91
UBALD1	-1.48066	9.63E-15

TMEM115	-2.44865	4.62E-173
CISH	-2.94648	1.39E-13
RBM15B	-4.08221	1.49E-211
RRP9	1.263365	2.24E-85
DUSP7	-2.33758	1.66E-115
WDR82	-1.44784	3.64E-132
ZNF500	-2.03587	4.35E-08
WNT5A	-1.48214	1.09E-97
ARHGEF3	-1.14481	3.30E-18
IL17RD	-1.05294	9.20E-06
DNAH12	-2.01162	1.52E-09
DENND6A	-1.39705	2.29E-54
ABHD6	-1.14723	1.30E-06
KCTD6	-2.4953	2.37E-13
ATXN7	-1.67742	7.44E-40
PRICKLE2	-1.79226	3.53E-240
ADAMTS9	-2.02712	0
KBTBD8	-2.77284	1.59E-11
LOC103227869	1.240663	1.48E-70
TMF1	-1.49547	1.01E-103
FRMD4B	-2.70964	2.38E-189
LOC103227890	-2.41429	1.40E-12
FOXP1	-1.63768	6.97E-215
RYBP	-1.95279	2.65E-106
EBLN2	-2.3188	2.36E-07
PPP4R2	-1.88284	4.58E-219
CRBN	-1.59668	1.05E-40
BHLHE40	-5.85757	0
SETD5	-1.67341	1.39E-244
JAGN1	-1.02944	4.20E-36
TMEM186	-3.15965	2.86E-62
VGLL4	-1.08171	1.63E-34
IQSEC1	-1.27135	2.78E-166
WNT7A	-1.24705	6.27E-42
USP7	-1.43543	2.37E-216
CCDC174	-2.36197	9.70E-61
C5H16orf72	-1.7439	1.66E-164
NR2C2	-1.41494	1.84E-89
TMCC1	-1.50439	8.34E-93
LOC103228071	-3.73631	2.06E-06
ATF7IP2	-2.08673	2.62E-67
GATA2	-1.57758	5.31E-36
MCM2	1.195524	0
KLF15	-5.38165	8.34E-11

SLC12A8	2.797019	5.66E-07
ZNF148	-1.27616	2.69E-81
HSPBAP1	-1.8108	1.15E-10
PARP14	-1.31357	4.00E-56
DTX3L	-1.97736	8.08E-53
WDR5B	-2.86184	4.11E-26
GOLGB1	-1.20976	1.88E-163
STXBP5L	-1.65281	3.46E-60
GSK3B	-1.29585	7.40E-122
LOC103228214	-1.69545	1.31E-04
ZDHHC23	-1.4953	8.48E-06
SOCS1	-3.45091	3.31E-05
USF3	-3.02985	4.22E-28
NEPRO	-2.51748	9.79E-141
TAGLN3	5.134054	7.11E-04
PLCXD2	-1.16292	2.90E-80
PHLDB2	-1.18173	2.16E-269
NECTIN3	-1.27697	2.19E-52
CBLB	-1.93772	1.79E-154
SNN	-2.62319	1.27E-60
NFKBIZ	-5.29492	0
CEP97	-1.07258	8.13E-18
ZBTB11	-2.59727	2.34E-123
TRMT10C	-3.22113	1.24E-169
LOC103228439	-1.2291	9.05E-68
LNP1	-1.10491	2.87E-04
FILIP1L	-4.56512	2.96E-229
GPR15	-2.87325	4.12E-177
RIOX2	1.532603	2.16E-50
ZNF654	-1.73384	6.62E-35
C22H3orf38	-3.20407	5.52E-100
ROBO2	-1.03298	2.64E-105
ERCC4	-2.81412	8.34E-68
ZNRD2	1.053505	1.44E-23
LOC103228631	-2.69884	5.71E-05
LOC103228639	-2.93786	3.72E-06
LOC103228659	-1.43093	2.99E-04
MRTFB	-1.06076	7.54E-73
NRL	-1.7083	7.19E-10
IRF9	-1.94706	2.98E-52
NFATC4	-1.22584	7.95E-12
NOVA1	-1.75672	2.02E-06
STRN3	-1.18369	5.90E-46
EGLN3	-1.10583	8.32E-31

NFKBIA	-4.17509	0
INSM2	-3.7177	1.68E-11
BRMS1L	-1.33446	1.68E-32
NKX2-8	-1.8569	1.61E-04
PAX9	-2.86595	7.22E-15
FOXA1	-2.39696	9.03E-14
FBXO33	-2.37936	3.40E-42
LRFN5	-1.66036	2.54E-21
C24H14orf28	-2.77284	1.19E-15
TOGARAM1	-1.12768	2.89E-21
KLHL28	-2.0754	2.92E-20
FANCM	-2.4051	1.16E-36
MGAT2	-2.29706	5.62E-183
DNAAF2	-1.63806	3.51E-19
ARF6	-2.87836	0
SOS2	-1.94612	4.90E-81
SAV1	-1.96932	6.14E-97
LOC103228964	-3.84323	6.26E-07
FRMD6	-1.97968	0
GPR137C	-1.06562	3.27E-05
BMP4	-5.4953	1.28E-11
CGRRF1	-1.33401	1.41E-16
SOCS4	-1.29251	1.16E-61
MAPK1IP1L	-1.45291	4.12E-130
FBXO34	-3.983	4.93E-214
ATG14	-2.93817	1.43E-52
ITPRIPL2	-2.11929	2.23E-21
NAA30	-1.8803	3.03E-157
ARID4A	-2.23018	1.24E-70
PPM1A	-1.12261	2.21E-75
SIX1	-2.60066	1.58E-28
SIX4	-2.48066	1.22E-14
TRMT5	-2.78481	4.06E-101
SLC25A45	-1.13334	2.56E-13
HIF1A	-1.27197	0
SNAPC1	-1.44897	2.94E-34
GPHB5	5.356446	1.24E-07
WDR89	-2.44018	8.30E-133
SYNE2	-1.03274	0
AKAP5	5.196901	0
ZBTB25	-3.14021	3.64E-46
ZBTB1	-1.70931	1.84E-71
HSPA2	-3.64512	1.05E-87
MAX	-1.09957	2.45E-27

MPP5	-1.12709	1.78E-161
LOC103229226	-3.91034	2.83E-07
ZFP36L1	-4.16195	0
LOC103229237	2.983831	9.11E-04
SUSD6	-1.92796	4.14E-56
LOC103229267	-1.43475	5.56E-05
MED6	-1.20026	1.38E-46
MAP3K9	-1.63602	4.92E-62
LOC103229289	3.71259	1.18E-04
ZFYVE1	-1.97526	3.52E-126
ELMSAN1	-3.05539	2.76E-144
ENTPD5	-1.2203	6.69E-09
FAM161B	-1.61011	3.60E-08
FCF1	-1.22279	1.24E-24
YLPM1	-1.35535	5.45E-143
RPS6KL1	1.166844	1.67E-09
ZC2HC1C	-1.94276	1.84E-22
ACSM3	-1.13677	1.08E-23
FOS	-3.82595	1.58E-12
LOC103229355	-2.4599	2.68E-30
TGFB3	-1.46118	6.14E-14
GPATCH2L	-1.31846	2.90E-17
IRF2BPL	-4.80376	3.18E-188
LOC103229379	-2.4051	2.11E-09
CIPC	-1.13516	7.39E-27
SAMD15	-1.41174	7.59E-06
THUMPD1	-1.42466	5.89E-72
DCUN1D3	-2.49368	5.27E-42
GTF2A1	-1.49185	2.97E-59
STON2	-1.53837	5.54E-07
SEL1L	-2.04574	7.60E-297
SPATA7	-1.20443	7.80E-19
FOXN3	-1.02316	5.23E-07
NRDE2	-2.73566	9.10E-114
PPP4R3A	-1.87427	1.43E-231
TRIP11	-1.34032	1.38E-67
RIN3	-1.41479	1.10E-31
GOLGA5	-1.27024	4.33E-67
TMEM251	-1.63114	4.16E-16
BTBD7	-2.86941	0
DICER1	-2.1247	3.13E-240
CDR2	-1.32628	1.47E-42
CDC42EP2	-2.73512	1.99E-62
CCNK	-1.2058	9.32E-25

LOC103229684	-1.53177	1.40E-60
SCNN1B	1.268984	3.76E-07
HSP90AA1	1.04693	0
WDR20	-2.16973	2.90E-50
TNFAIP2	-1.11318	1.38E-21
TRMT61A	1.179716	2.22E-32
BAG5	-1.16866	8.83E-42
PPP1R13B	-1.21387	2.08E-16
AHNAK2	-1.35214	0
GPR132	-2.01859	5.34E-13
CDCA4	-1.92685	1.01E-59
RBBP6	-3.42154	0
TNRC6A	-2.01569	1.15E-196
GCH1	1.069099	3.20E-04
MLH3	-1.87175	1.61E-22
SPTLC2	-1.25245	5.56E-54
ALKBH1	-1.86119	4.35E-14
HIST3H2A	-1.53587	3.34E-29
TRIM11	-1.24848	1.26E-37
ARHGAP17	-1.08195	2.33E-34
LOC103229976	-1.85236	2.82E-21
LOC103229978	-1.62083	8.18E-08
SDE2	-2.15955	1.72E-28
ZKSCAN2	-3.19148	1.22E-95
FBXO28	-2.27417	4.27E-136
TLR5	-4.42491	3.45E-66
DISP1	-1.21009	4.34E-21
MIA3	-1.0784	1.26E-68
DUSP10	-3.54894	6.79E-19
HLX	-2.94276	7.89E-26
KDM8	-1.04995	6.65E-04
TGFB2	-2.59072	0
GPATCH2	-1.27647	6.32E-13
ESRRG	-1.29249	4.38E-33
SYVN1	-1.15662	2.73E-61
CENPF	-1.22302	0
VASH2	-1.41255	5.96E-275
ATF3	-2.1718	1.68E-100
NEK2	-1.77734	1.18E-58
SLC30A1	-1.08378	7.91E-42
TRAF5	-1.35266	5.13E-12
KCNH1	2.249531	3.06E-04
C25H1orf74	-4.19238	2.31E-47
YOD1	-4.19686	6.99E-117

C25H1orf116	-1.86184	1.16E-08
PIGR	2.278685	5.49E-48
IL10	4.341947	4.04E-04
DYRK3	-3.41834	1.27E-28
SLC45A3	1.25546	5.28E-41
ELK4	-1.79283	1.39E-27
MFSD4A	-1.49398	6.72E-14
NUAK2	-2.5693	0
DSTYK	-1.20517	1.33E-39
RBBP5	-1.06846	3.09E-22
PPP1R15B	-3.99333	1.99E-248
ZBED6	-3.50444	1.23E-119
ZC3H11A	-1.58882	2.07E-277
BTG2	-4.49074	8.03E-236
KDM5B	-2.98604	4.89E-209
ARL8A	-2.78888	2.98E-141
GPR37L1	-3.73631	2.06E-06
ELF3	-5.65097	0
NAV1	-1.10954	3.89E-134
TNNI1	3.567726	3.77E-70
INAVA	-1.20716	6.20E-95
CAMSAP2	-2.10038	2.50E-183
DDX59	-1.11507	4.87E-25
ZNF281	-5.32032	5.36E-239
LOC103230365	-1.6391	4.56E-62
CRB1	-2.24099	6.42E-08
ZBTB41	-1.76569	7.17E-49
ASPM	-2.60734	0
RGS2	-4.40057	0
HMCN1	-1.15718	5.31E-09
IVNS1ABP	-1.06077	1.21E-131
SWT1	-1.10912	4.15E-10
RNF2	-1.17498	8.91E-42
EDEM3	-1.66434	1.97E-149
TSEN15	1.149112	5.32E-58
RGL1	-1.90805	3.13E-125
SLX1A	-1.24014	1.86E-11
RNASEL	-2.04307	1.40E-44
GLUL	-2.58743	0
IER5	-3.97447	4.57E-78
LOC103230489	-1.26422	1.71E-18
KIAA1614	-1.17903	7.20E-08
CEP350	-1.76676	3.48E-127
TOR1AIP2	-1.26389	4.89E-66

ABL2	-2.67855	3.68E-92
RASAL2	-1.12028	7.13E-26
ZBTB37	-2.17883	2.59E-05
PIGC	-1.63162	9.96E-37
FMO4	-1.34753	3.49E-15
GORAB	-1.62568	6.24E-17
METTL18	-3.47839	6.12E-55
ATP1B1	-1.15612	4.74E-274
ADCY10	-2.23227	1.32E-05
TADA1	-3.14362	2.44E-109
POGK	-2.43316	1.31E-130
RHOU	-2.27873	4.66E-07
CCSAP	-2.52891	2.11E-15
ACTA1	-2.15135	3.81E-04
URB2	-1.66088	6.96E-39
FAM89A	1.163563	1.52E-27
SPRTN	-1.31652	8.31E-16
EXOC8	-3.76728	7.60E-71
EGLN1	-1.23993	3.40E-53
MAP3K21	-1.67079	3.72E-22
PAGR1	-1.25124	1.42E-37
IRF2BP2	-4.49031	0
ARID4B	-2.67624	4.61E-277
LYST	-1.29306	1.52E-43
ZNHIT2	-2.55044	1.59E-12
AKT3	-1.07272	4.54E-117
ZBTB18	-3.69986	7.25E-188
KIF26B	-1.10491	2.87E-04
ZNF48	-1.92664	4.95E-19
KIF28P	-1.56312	1.58E-08
ZNF670	-2.33843	1.68E-07
ZNF124	-3.11387	5.44E-10
LOC103230923	-1.89002	2.20E-10
DCTPP1	1.113026	4.86E-161
ZNF496	-1.01462	7.31E-27
LOC103230939	-1.17382	9.74E-43
LOC103230963	-2.3069	1.56E-31
PGBD2	-3.86595	8.90E-19
SRCAP	-1.50672	1.05E-184
H3F3A	-2.0355	1.20E-113
WDR26	-1.13095	5.06E-192
PRRC2C	-1.16109	1.15E-222
AHCTF1	-2.02031	4.93E-197
ZNF629	-1.13554	8.57E-47

WHAMM	-1.78939	4.31E-17
ALPK3	-1.5327	2.42E-07
ZNF592	-1.46413	3.87E-95
ZSCAN2	-1.5567	5.06E-11
AKAP13	-2.08081	0
DET1	-1.22944	1.20E-16
AEN	-1.65506	9.71E-82
PEX11A	-2.25826	3.23E-09
PLIN1	-1.83697	2.12E-08
SEMA4B	-1.05911	1.48E-33
SETD1A	-1.67352	6.75E-122
GDPGP1	-3.4953	1.26E-09
ZNF774	-2.0089	1.26E-23
CHD2	-2.77058	0
LOC103231220	-1.16363	8.42E-07
PRSS53	2.549091	6.83E-04
NR2F2	-2.76249	6.06E-44
ARRDC4	-2.11007	1.04E-114
ZNF646	-2.85652	4.76E-82
ZNF668	-2.77284	2.92E-38
LYSMD4	-3.72393	9.96E-17
ASB7	-2.16663	2.40E-67
CHSY1	-1.94733	6.29E-28
LOC103231340	1.63122	1.52E-53
LOC103231342	-4.80176	1.93E-07
LOC103231344	-1.05471	5.23E-37
PNP	1.221582	2.27E-140
TMEM253	-1.48653	5.89E-05
CHD8	-1.41599	4.92E-255
SALL2	-3.46941	1.35E-189
ARMC5	-2.57744	4.86E-51
ABHD4	-1.03493	9.38E-122
C29H14orf93	-1.13163	9.63E-18
C29H14orf119	-2.26907	4.71E-89
RNF212B	-4.09476	2.60E-08
HOMEZ	-3.71713	3.06E-170
NGDN	-1.85917	1.18E-41
ZFHX2	-3.42245	5.51E-64
TICRR	-1.69212	4.68E-37
ZNF267	-4.17883	1.93E-54
ZBED1	-1.04773	7.18E-34
SHROOM2	-2.25104	7.97E-163
TCEANC	-3.3578	1.31E-08
ZRSR2	-1.13856	2.96E-13

CDKL5	-1.72393	1.62E-14
BCLAF3	-1.73867	8.14E-28
MAP7D2	-1.1368	1.07E-05
LOC103231695	-1.38735	3.15E-51
SMS	-1.40979	0
KLHL15	-1.86431	1.46E-38
GK	-1.07425	6.70E-13
CREBBP	-1.83713	2.90E-104
MID1IP1	-2.13299	2.37E-72
BCOR	-3.57469	1.76E-136
DDX3X	-1.99093	0
USP9X	-1.10664	0
FUNDC1	-1.08269	9.23E-35
KDM6A	-1.40461	1.92E-93
PALB2	-4.01569	1.95E-140
JADE3	-1.44546	3.89E-153
LOC103231885	-1.38252	9.29E-10
ZNF41	-2.2058	8.77E-25
ZNF182	-1.00248	4.23E-16
LOC103231911	-1.20026	1.08E-07
LOC103231919	-1.07846	1.20E-16
TBC1D25	-1.2609	6.64E-21
SUV39H1	-1.00238	2.42E-21
PIM2	-1.56071	3.10E-22
CCDC120	-1.71561	1.00E-30
BATF2	-1.23113	3.55E-12
SHROOM4	-1.46848	5.58E-20
MAGEH1	-1.12076	2.39E-05
USP51	-2.30889	4.75E-06
UBQLN2	-2.04943	2.99E-132
LOC103232061	-1.87071	1.83E-209
SPIN4	-1.4618	3.18E-13
AMER1	-1.45091	3.88E-10
EDA2R	-1.23797	1.62E-77
EFNB1	-1.63924	2.62E-110
PJA1	-2.29982	1.12E-27
EDA	-1.14908	4.24E-11
GCNA	-1.93634	6.36E-05
RTL5	-1.64185	4.39E-27
ERCC6L	-2.94719	1.71E-161
CITED1	3.134054	3.01E-05
RLIM	-2.36129	2.63E-72
MAGEE1	-2.37691	5.04E-21
DNAJA2	-1.14858	5.83E-82

LOC103232262	-1.66214	2.03E-77
HDX	-1.2838	1.58E-07
PABPC5	-4.05082	1.34E-28
PCDH11X	2.451837	0
HNRNPH2	-1.27637	2.42E-124
ARMCX4	-1.39822	2.08E-80
LOC103232367	7.610436	2.08E-15
GPRASP1	-1.252	2.13E-09
LOC103232389	1.829199	3.05E-04
BEX4	-1.08022	6.73E-56
TCEAL1	-1.15471	2.05E-21
RAB9B	-1.42265	6.29E-21
PWWP3B	-2.74859	3.22E-07
SIAH1	-2.79076	6.69E-60
CLDN2	-1.52007	2.91E-57
RBM41	3.230572	0
TSC22D3	-2.41526	0
VSIG1	1.847817	2.45E-04
RTL9	-2.52286	1.97E-18
N4BP1	-1.30602	1.24E-53
APLN	2.257629	3.12E-17
BCORL1	-2.00214	7.98E-99
RAB33A	-4.2058	4.63E-05
ZNF75D	-1.03587	1.52E-06
CNEP1R1	-2.79938	3.23E-75
ZIC3	-3.17883	3.77E-26
LOC103232720	-2.43713	2.93E-10
MTM1	-1.17635	5.42E-20
SALL1	-3.60741	6.50E-71
ZNF275	-1.23211	5.39E-41
CHD9	-1.33261	2.67E-113
ZFX	-1.74864	8.98E-28
USP27X	-3.32865	3.16E-19
IRX3	-3.40138	8.59E-13
IRX5	-4.09476	2.60E-08
LPCAT2	-1.09277	2.26E-06
LOC103233030	1.09141	8.94E-14
LOC103233031	1.237963	2.26E-31
LOC103233043	1.165116	6.06E-22
MT1X	-1.05082	2.11E-06
HERPUD1	-1.66654	1.08E-128
RSPRY1	-2.57037	3.35E-299
KATNB1	1.066172	9.63E-94
ZNF319	-1.10471	1.29E-25

USB1	1.213536	9.92E-114
CMTM2	5.134054	7.11E-04
PDP2	-2.39432	5.88E-141
RRAD	-2.83338	1.32E-16
FA2H	-1.08478	2.78E-06
RFWD3	-1.22824	5.14E-52
CMTR2	-3.60741	4.55E-43
MARVELD3	3.685502	2.62E-06
CHST4	-1.75672	2.02E-06
ZNF19	-3.57429	7.86E-33
AP1G1	-1.34614	2.63E-226
ATXN1L	-1.59341	3.21E-134
ZFHX3	-1.81595	5.43E-191
NOB1	-1.07087	1.83E-55
PDF	1.018089	1.11E-16
COG8	-1.54856	1.32E-74
LOC103233222	-4.62182	4.15E-21
ZFP90	-1.11295	4.44E-47
SMPD3	-1.74159	3.58E-09
DDX28	-3.5603	1.47E-63
THAP11	-1.98972	7.88E-74
CTCF	-1.48306	4.22E-103
C5H16orf70	-2.75405	3.82E-84
ZFP1	-3.89937	2.65E-37
BCAR1	-2.18735	2.69E-217
TMEM170A	-1.24532	4.39E-05
MON1B	-1.13576	2.88E-36
MAF	-2.97447	7.73E-09
C5H16orf46	-1.91034	4.81E-04
ATMIN	-2.28591	1.81E-114
GAN	-1.09263	1.58E-20
SDR42E1	-1.89569	1.53E-39
MLYCD	-1.59986	2.35E-36
OSGIN1	-1.15517	4.55E-13
NECAB2	2.461629	1.10E-09
LOC103233384	-1.33693	2.06E-04
MEAK7	-1.05247	5.30E-13
CRISPLD2	-1.87739	1.19E-109
FERMT3	1.347458	3.31E-08
C5H16orf74	1.041058	1.22E-16
FOXC2	-4.94276	4.07E-15
FBXO31	-1.00935	6.78E-46
ZCCHC14	-1.3897	5.28E-107
SLC7A5	1.695231	0

ZFPM1	-2.08478	8.05E-28
ZC3H18	-1.55488	3.82E-118
CDT1	-1.47303	4.89E-67
CBFA2T3	-1.93984	7.54E-18
LOC103233468	3.549091	9.29E-09
ZNF778	-1.90556	8.64E-41
CDK10	1.014813	6.84E-45
FLRT1	-2.71394	6.84E-11
LOC103233485	1.219014	5.73E-47
LOC103233486	2.001604	1.59E-06
SLC25A22	1.256484	1.83E-89
ODF3L2	-1.0929	2.16E-23
ARID3A	-1.67587	8.30E-22
MISP	-1.83296	4.28E-123
MIDN	-3.3116	1.66E-195
EFNA2	-1.69377	3.80E-09
ADAT3	-2.55943	1.01E-07
MOB3A	-1.55338	2.62E-39
MKNK2	-1.59544	4.37E-197
GADD45B	-2.33553	1.13E-295
ZNF554	-2.68357	1.42E-10
ZNF555	-3.2375	1.00E-17
ZNF57	-2.9587	3.13E-11
LOC103233665	-2.82595	1.15E-18
LOC103233674	-1.82942	2.19E-04
TBXA2R	-1.08366	3.43E-04
MATK	1.295598	1.15E-20
LRG1	-3.45091	1.95E-25
PLIN4	-2.15492	2.46E-32
ZBTB7A	-2.08834	2.47E-125
TNFAIP8L1	-1.47809	2.02E-18
FEM1A	-3.79609	4.98E-111
TICAM1	-4.74246	1.29E-72
KDM4B	-1.16322	5.77E-88
SAFB	-1.92985	0
SAFB2	-1.6546	7.26E-113
LOC103233755	-1.83067	3.58E-04
CAPS	-1.09133	2.74E-27
ZNF557	-1.45605	7.94E-14
CAMSAP3	-2.02834	1.83E-28
LRRC8E	-2.08358	2.42E-53
ZNF558	-3.25826	6.57E-45
ZNF317	-3.1575	4.65E-56
ZNF699	-4.77284	1.95E-13

ZNF266	-4.41669	1.04E-19
ZNF121	-2.57192	1.76E-09
LOC103233870	-2.46471	3.68E-58
ZNF846	-1.00111	3.36E-04
ZNF562	-1.2815	8.92E-13
S1PR2	1.612769	2.57E-21
ZBTB3	-5.65058	2.06E-24
S1PR5	-3.37374	1.70E-12
TIMM29	-1.1759	5.43E-27
KANK2	-1.61864	0
ANGPTL8	-2.38808	1.27E-30
EPOR	-1.97447	2.55E-04
SWSAP1	-3.63413	4.40E-44
ZNF653	-1.42953	6.45E-13
ZNF627	-3.64918	9.38E-69
ZNF823	-7.38165	3.05E-37
LOC103233944	-3.03738	9.19E-23
LOC103233945	-2.73631	4.08E-13
LRRN4CL	-1.85616	7.39E-51
LOC103233952	-1.63678	5.02E-06
ZNF136	-2.30436	1.55E-14
ZNF44	-3.5323	1.19E-31
ZNF709	-4.53837	2.12E-11
LOC103233973	-6.21246	2.98E-35
ZNF490	-3.55943	3.89E-10
C1H11orf83	1.227163	1.04E-77
JUNB	-6.41689	0
INTS5	-2.79203	1.16E-118
IER2	-2.48918	8.69E-141
DNAJB1	-3.01554	0
ZNF333	-1.26853	2.62E-06
TUT1	-1.18155	1.81E-18
RAB8A	-1.22888	6.09E-80
CHERP	-1.12951	5.64E-68
MED26	-3.2394	1.54E-37
F2RL3	5.71259	2.61E-05
HAUS8	-1.40568	6.49E-28
ANKLE1	4.216984	1.46E-06
MAP1S	-1.64713	2.34E-75
RAB3A	-2.26561	2.23E-17
IQCN	-2.45091	2.66E-34
JUND	-3.80655	0
GDF15	-2.25663	6.17E-73
ELL	-2.15806	1.24E-67

KLHL26	-2.0731	3.13E-47
DDX49	1.087554	2.37E-68
ZNF14	-5.50619	3.57E-22
ZNF101	-3.13273	3.71E-10
TSSK6	-2.4051	7.22E-04
LOC103234233	-2.2964	2.14E-20
ZNF253	-1.68357	2.14E-06
LOC103234239	-4.99286	2.03E-08
LOC103234241	-3.38079	3.92E-20
LOC103234249	-2.69037	3.42E-18
LOC103234255	-3.60741	3.37E-15
DAGLA	-1.30797	1.06E-60
LOC103234265	-2.10912	8.68E-09
LOC103234275	-1.41774	7.24E-05
ZNF675	-5.47328	1.86E-11
MIER2	-1.64702	6.30E-46
APC2	-1.15839	9.86E-37
PIAS4	-1.88644	2.57E-59
YJU2	-1.42152	9.81E-12
QTRT1	1.229183	3.95E-30
LDLR	-1.66017	0
ZNF441	-4.06562	4.28E-15
ZNF791	-5.25826	9.23E-19
ZSWIM4	-1.07827	1.12E-17
RFX1	-1.15802	2.86E-18
LOC103234377	-1.00248	1.20E-08
PGPEP1	-1.3578	1.23E-06
CRACR2B	2.175277	1.98E-17
POP4	-1.30084	4.16E-18
PLEKHF1	-2.79076	1.29E-09
ZNF507	-1.97663	3.85E-93
RHPN2	-1.20204	1.73E-87
CEBPA	-1.45091	1.69E-15
LOC103234473	-1.56874	4.35E-12
LOC103234475	-1.67317	1.58E-13
WTIP	1.164232	2.42E-30
ZNF599	-5.41669	6.97E-21
ZNF792	-2.12333	5.03E-05
FFAR2	5.134054	7.11E-04
KMT2B	-2.40463	7.19E-136
LIN37	-2.25494	1.76E-36
NFKBID	-3.03587	2.48E-09
LRFN3	-3.31244	9.25E-51
SDHAF1	-2.0055	3.89E-11

ZNF565	-2.03587	5.91E-09
ZNF146	-1.34251	3.07E-116
TSPAN4	1.082578	6.36E-88
ZFP82	-2.88831	3.45E-15
ZNF260	-1.31904	1.34E-24
ZNF567	-2.95341	3.70E-16
ZNF382	-1.13103	1.92E-05
ZNF345	-1.60291	2.66E-05
ZNF420	-3.26853	3.18E-18
LOC103234590	-1.77424	4.86E-11
ZNF585B	-2.96807	3.89E-14
ZNF383	-2.62971	4.26E-17
ZNF875	-4.24532	2.25E-33
ZNF570	-2.83338	1.32E-16
C6H19orf33	1.891979	3.98E-11
GGN	-2.08318	9.31E-06
LRFN1	-1.9587	5.02E-26
ZFP36	-4.29849	9.13E-102
EID2B	-2.554	6.73E-45
EID2	-2.35459	1.00E-39
DYRK1B	-1.1916	1.91E-39
ZNF546	-1.38502	1.26E-05
SERTAD1	-2.99729	4.33E-79
SERTAD3	-4.88554	2.40E-107
C6H19orf54	-1.6466	1.07E-55
ARHGEF1	1.11112	4.00E-119
ZNF574	-2.94632	6.37E-90
ZNF526	-3.2838	2.66E-56
DEDD2	-1.64611	6.08E-66
ERF	-2.62849	2.67E-166
CIC	-1.29933	6.11E-229
CEACAM8	4.009479	4.10E-29
PHLDB3	-1.32776	5.66E-17
SRRM5	-1.91393	4.56E-04
ZNF45	-1.5323	1.09E-12
ZNF221	-2.45091	2.09E-08
ZNF234	-1.19199	6.45E-09
ZNF225	-1.07149	1.44E-04
ZNF284	-3.62083	1.20E-10
LOC103234816	-1.62563	1.01E-48
ZNF226	-2.84684	1.72E-43
ZNF235	-2.97447	1.76E-16
ZNF112	-2.53306	1.57E-26
ZNF285	-2.58019	1.41E-14

ZNF180	-3.38165	6.34E-24
BCL3	-3.28004	2.54E-186
BCAM	-1.21622	3.33E-87
TOMM40	1.31384	0
BLOC1S3	-2.03587	2.38E-06
CD3EAP	-1.52185	3.72E-26
PPP1R13L	-1.13627	1.77E-37
OPA3	-1.68303	8.33E-16
FBXO46	-2.04627	5.00E-23
SIX5	-1.4472	6.99E-53
OSBP	-1.68988	8.07E-251
FOXA3	-1.80141	2.97E-04
IRF2BP1	-2.92896	6.15E-86
NOVA2	-2.60471	2.23E-18
CCDC8	-3.35602	3.66E-200
ARHGAP35	-1.67707	5.44E-247
ZC3H4	-4.14826	1.52E-134
BBC3	-5.37232	4.43E-267
BICRA	-3.98489	4.99E-40
EHD2	-1.28741	7.04E-05
DBP	-1.80386	1.46E-15
KCNA7	3.046403	3.62E-07
PRR12	-3.52237	3.52E-173
SCAF1	-1.06502	1.25E-91
NUP62	-1.30501	5.75E-82
ZNF473	-1.97447	4.89E-66
MYBPC2	-1.56639	9.90E-04
LRRC4B	-1.13896	1.03E-04
CLEC11A	-1.04386	5.40E-06
ZFP91	-1.25881	1.07E-95
CTU1	-1.92896	1.21E-11
VSIG10L	-1.10072	1.84E-06
ZNF175	-1.45431	3.01E-20
ZNF613	-5.3578	4.54E-20
ZNF649	-1.91034	1.58E-05
ZNF577	-1.2235	5.91E-04
ZNF350	-2.65254	2.60E-19
ZNF614	-3.52416	4.03E-27
ZNF841	-2.6733	2.50E-12
ZNF836	-2.78719	1.10E-11
ZNF616	-1.77284	2.43E-05
ZNF766	-3.2235	3.14E-40
ZNF347	-2.77284	1.19E-15
LOC103235192	-1.43073	3.00E-04

ZNF808	-2.23311	9.84E-07
ZNF331	-2.91688	3.50E-25
MYADM	-1.63887	1.26E-52
TMC4	-1.19634	6.13E-05
LENG1	-1.71525	8.50E-14
LENG9	2.137322	1.30E-04
KMT5C	-1.88275	5.66E-18
TMEM190	1.631554	1.38E-09
TMEM238	-1.08318	9.24E-04
ZNF628	-3.04016	3.27E-39
ZNF579	-2.94445	5.49E-48
FIZ1	-1.30452	7.46E-17
ZNF524	-1.18362	1.44E-08
ZNF865	-1.86369	2.23E-28
ZNF784	-4.05379	1.66E-35
ZNF581	-2.41464	3.73E-202
ZDHHC5	-1.61396	1.09E-240
ZNF787	-1.30889	1.21E-26
CLP1	-3.63854	3.37E-87
ZNF667	-1.75347	6.72E-10
ZNF583	-2.17337	2.49E-06
ZNF582	-2.97447	2.15E-11
ZNF471	-1.05724	2.67E-12
ZNF71	-3.60066	5.61E-29
ZNF264	-3.58019	1.01E-05
ZNF805	-1.59596	4.26E-07
ZNF304	-3.2375	3.49E-34
ZNF547	-4.2058	8.35E-17
LOC103235394	-2.8077	4.06E-04
ZNF17	-3.2058	1.28E-23
ZNF549	-2.52332	8.18E-28
ZNF211	-1.99656	1.62E-26
ZNF134	-3.89258	7.72E-79
ZNF416	-5.17883	8.98E-18
ZNF606	-4.4051	9.32E-06
LOC103235422	-3.19148	1.79E-48
RTN4RL2	-1.43276	5.75E-22
LOC103235424	-2.94276	7.89E-26
ZNF776	-2.0327	9.56E-34
LOC103235431	-4.30889	4.46E-35
ZNF551	-2.34399	4.22E-10
ZNF329	-1.54763	1.53E-10
ZNF274	-2.8553	2.18E-39
ZNF8	-2.3638	5.17E-22

ZSCAN22	-2.69884	1.52E-14
ZNF837	-2.19814	1.23E-08
ZNF446	-1.27046	3.63E-27
LOC103235452	-2.81116	4.20E-29
ZBTB45	-1.17941	2.14E-21
TRIM28	1.090797	0
MZF1	1.122277	6.69E-12
TNKS1BP1	-2.01135	4.32E-227
CCNE1	1.205205	9.68E-64
CEBPG	-1.98394	1.84E-80
LOC103235514	-1.38165	3.06E-06
LOC103235575	-1.60934	1.08E-07
ZNF584	-1.32669	1.63E-15
LOC103235581	-4.94276	3.77E-08
YTHDC1	-1.34749	2.66E-109
CENPC	-1.4934	7.14E-19
REST	-1.92059	4.47E-76
AASDH	-1.43398	4.30E-53
CEP135	-1.68868	5.69E-17
TMEM165	-1.25738	1.75E-59
CHIC2	-1.23227	9.17E-11
RASL11B	-4.67008	8.87E-24
LRRC66	4.549091	1.15E-04
UTP3	-3.25966	1.36E-125
ANKRD17	-2.11626	0
CXCL8	3.201168	7.86E-09
CXCL1	1.85842	8.02E-12
CXCL3	2.442176	1.45E-04
SDAD1	-1.12687	4.16E-38
STBD1	-3.13183	1.32E-247
SHROOM3	-1.68357	3.00E-27
CCNG2	-3.65385	1.40E-204
CNOT6L	-2.4181	7.53E-228
BMP2K	-1.33309	1.14E-153
PRDM8	-3.42819	4.06E-09
FGF5	-1.72763	1.86E-53
RASGEF1B	-4.61414	1.37E-33
PTPRJ	-1.1984	2.04E-54
HNRNPD	-1.0271	4.96E-234
THAP9	-1.45783	2.29E-29
FNBP4	-2.18089	2.00E-124
LIN54	2.440831	0
HELQ	-2.90721	5.83E-51
CDS1	-1.252	2.13E-09

WDFY3	-1.62242	1.13E-239
ARHGAP24	-2.17189	1.19E-61
AFF1	-2.46214	1.06E-82
PYURF	-1.12198	1.58E-44
NAP1L5	-1.96778	4.52E-38
TIGD2	-3.13896	3.45E-28
GPRIN3	-2.05223	1.57E-84
SMARCAD1	-1.98289	1.99E-167
KBTBD4	-1.40484	1.04E-58
EIF4E	-1.48528	2.21E-128
TET2	-2.27508	3.12E-79
INTS12	-1.91906	3.39E-45
LOC103236086	-1.82813	1.95E-98
SGMS2	-1.43554	1.04E-119
SEC24B	-3.14943	0
CFI	-1.2838	7.00E-14
PITX2	-1.41438	1.22E-19
ALPK1	-1.12942	5.94E-21
ZGRF1	-1.13228	3.43E-09
ARSJ	-1.37822	3.63E-146
UGT8	-1.20719	2.58E-27
TRAM1L1	-3.77284	5.15E-12
METTL14	-1.79668	4.74E-75
PDE5A	-1.82333	6.14E-48
C7H4orf3	-1.01373	1.42E-115
LOC103236197	-3.91034	3.47E-04
KIAA1109	-1.36246	6.35E-158
BBS12	-4.05247	8.42E-63
SPRY1	-4.55943	6.44E-22
ANKRD50	-3.67923	2.22E-194
FAT4	-3.25826	1.60E-04
PLK4	-2.54683	2.00E-113
PGRMC2	-1.84811	0
JADE1	-3.18575	0
PABPC4L	-2.49044	8.60E-15
NOCT	-2.09078	9.39E-55
ELF2	-2.76266	2.69E-131
RAB33B	-2.30008	1.11E-21
IL15	-1.82014	1.86E-18
USP38	-2.40096	4.18E-123
GAB1	-1.69242	5.86E-45
OTUD4	-1.23865	2.86E-159
ANAPC10	-1.00047	3.45E-17
SMAD1	-2.26588	2.08E-63

MMAA	-1.68795	3.10E-12
ZNF827	-2.01627	1.66E-295
PRMT9	-2.03387	1.31E-52
LOC103236354	-1.87114	1.18E-54
NR3C2	-1.15135	8.31E-05
FHDC1	-3.77284	1.39E-06
LOC103236415	-3.14943	8.03E-89
HARBI1	-3.2838	5.01E-15
PDGFC	-1.38182	4.74E-264
GASK1B	-3.2058	2.37E-04
AMBRA1	-1.33938	4.62E-61
FNIP2	-1.71336	1.54E-29
RAPGEF2	-1.68177	1.38E-98
NPY1R	-2.33843	1.68E-07
MSMO1	-1.28133	0
TLL1	-1.31045	3.76E-89
CLCN3	-1.01471	7.89E-135
MFAP3L	-1.17337	8.01E-12
DUSP8	-3.17337	3.36E-32
TENM3	-1.05674	4.04E-229
CDKN2AIP	-2.52701	2.77E-48
IRF2	-1.21564	7.65E-50
ANKRD37	-2.04917	1.02E-32
LRP2BP	-1.07172	2.89E-10
CCDC110	-1.20894	4.88E-05
TLR3	-1.66988	5.06E-78
FAT1	-1.30698	0
LOC103236665	1.113742	2.94E-232
CRY2	-1.13849	5.98E-32
CEBPD	-5.49114	0
SNAI2	-6.5098	2.80E-125
IFITM10	-1.01651	6.26E-05
RB1CC1	-1.61925	1.02E-48
LOC103236809	-1.25826	2.04E-04
RP1	-1.15786	1.20E-04
TGS1	-1.72602	3.98E-185
PLAG1	-2.38538	2.87E-102
FAM110B	-2.48433	3.36E-13
RAB2A	-1.78862	9.49E-300
CHD7	-1.2998	3.62E-123
YTHDF3	-1.39706	1.12E-132
ADHFE1	-1.12647	4.00E-08
RRS1	-2.01741	1.17E-50
VCPIP1	-4.1416	1.52E-148

CSPP1	-1.01491	7.76E-17
ARFGEF1	-2.33944	0
MSC	-3.24099	3.73E-11
RDH10	-1.57465	1.48E-208
ZFHX4	-2.95473	4.94E-41
ZC2HC1A	-1.01958	1.75E-30
IL7	-1.39432	5.64E-39
ZBTB10	-3.44589	1.85E-145
PAG1	-2.3578	1.92E-42
SNX16	-1.45091	5.37E-18
TMEM64	-1.02316	1.18E-160
NECAB1	-3.90094	2.34E-43
C8H8orf88	-1.02849	2.10E-16
RUNX1T1	-2.29655	2.25E-204
RBM12B	-3.42643	1.10E-154
PDP1	-1.94567	8.77E-85
GEM	-1.21287	5.23E-53
VIRMA	-1.68762	0
TP53INP1	-3.56756	1.48E-265
PLEKHF2	-3.60795	2.21E-117
TSPYL5	5.134054	7.11E-04
OSR2	-3.13741	3.36E-13
FBXO43	-2.13896	4.47E-10
RNF19A	-2.01432	4.19E-137
ZNF706	-1.33008	5.06E-181
RRM2B	-1.04723	2.10E-79
KLF10	-3.86916	0
SLC25A32	-1.37398	1.78E-21
TMEM74	-2.42053	1.55E-34
KCNV1	-2.53837	3.82E-17
LRRC4C	-1.77284	4.01E-04
TRPS1	-1.93984	7.92E-134
UTP23	-1.24329	1.24E-41
ENPP2	-1.30889	5.96E-09
DEPTOR	-1.32953	1.25E-100
COL14A1	-1.23227	5.55E-05
ZHX2	-2.03869	7.57E-66
LOC103237375	-2.60038	2.62E-06
ZHX1	-2.10268	4.41E-151
FBXO32	-2.9481	0
RNF139	-3.07149	7.07E-281
TRMT12	-2.24232	2.39E-30
SQLE	-2.91044	0
ZNF572	-3.60066	3.33E-38

TRIB1	-5.50132	0
LRATD2	-4.09717	2.01E-86
LOC103237427	-1.39221	1.86E-11
MYC	-3.09949	0
LRRC6	-1.30596	7.46E-06
SLA	-2.62083	1.19E-04
TG	-1.07103	9.23E-44
NDRG1	-1.07031	0
CHRAC1	-2.30323	3.05E-107
PSCA	-3.30889	1.08E-04
JRK	-1.7856	1.62E-28
TRAF6	-3.00301	8.41E-98
RHPN1	1.044523	2.21E-20
MAFA	-4.15135	6.93E-05
TIGD5	-1.86119	6.23E-27
PYCR3	1.375528	2.76E-170
ZNF623	-1.8478	6.24E-51
ZNF707	-2.63082	2.36E-15
FAM83H	-2.8394	5.83E-41
NRBP2	1.437784	1.79E-100
SCRIB	1.016178	5.02E-159
BOP1	1.159663	2.37E-176
FBXL6	1.106437	1.77E-35
ADCK5	1.103839	2.30E-68
TONSL	1.218258	2.24E-139
RECQL4	1.143879	6.45E-61
MFSD3	1.246195	1.86E-37
KIFC2	1.248077	4.09E-50
FJX1	-3.97741	0
ZNF34	-2.1	8.16E-22
ZNF7	-1.15504	6.55E-38
COMMD5	-1.49412	1.27E-29
ZNF250	-2.06404	2.94E-23
LOC103237660	-2.2612	2.51E-41
ATAD2	-1.01851	9.52E-134
WDR37	-1.0913	8.06E-37
IDI1	-1.59324	0
KLF6	-4.47689	0
NET1	-2.26469	2.75E-240
TASOR2	-1.25525	1.94E-69
IL15RA	1.408229	5.91E-11
KIN	-2.15606	2.03E-48
USP6NL	-1.68931	5.90E-127
PROSER2	-2.29418	5.01E-62

MCM10	1.146259	2.52E-92
RPP38	-2.39108	8.39E-55
NMT2	-1.28773	9.26E-73
FAM171A1	-1.01811	1.14E-93
SKIDA1	-1.9319	1.46E-09
LOC103237988	-1.3328	2.97E-67
SPAG6	-1.47644	9.61E-04
PIP4K2A	-1.17226	5.40E-109
MSRB2	-1.12588	7.13E-46
OTUD1	-6.34573	7.71E-20
KIAA1217	-1.45369	7.90E-94
ARHGAP21	-1.4897	5.35E-132
THNSL1	-2.25323	9.94E-48
WAC	-1.00074	2.07E-197
DEPDC7	-1.2394	1.55E-14
BAMBI	-4.69503	1.33E-58
JCAD	-1.36625	3.35E-81
MTPAP	-2.88387	6.76E-163
ARHGAP12	-2.47964	2.32E-130
EPC1	-3.72237	1.01E-124
NRP1	-1.65183	1.58E-241
CUL2	-1.11563	8.03E-58
FZD8	-2.2058	1.25E-07
LARP4B	-1.84881	2.75E-115
LRRK2	-1.24947	5.64E-36
PRICKLE1	-4.49782	8.41E-257
ARID2	-2.13674	1.88E-83
SLC38A2	-4.98877	0
AMIGO2	-2.59972	1.08E-55
VDR	-1.2199	4.93E-24
ASB8	-1.43351	1.74E-26
KANSL2	-1.395	3.88E-104
CCNT1	-2.98348	3.10E-303
KMT2D	-1.13501	1.04E-92
TROAP	-1.62083	4.51E-37
BCDIN3D	-1.18903	1.46E-28
PAX6	-2.48123	0
METTL7A	-1.06708	9.08E-17
FIGNL2	-2.59387	2.86E-06
ACVR1B	-2.79668	2.80E-159
LOC103238332	-3.87717	4.21E-07
NR4A1	-1.69037	6.44E-06
LOC103238343	1.451944	1.84E-73
LOC103238345	1.080939	2.33E-28

KRT5	1.026933	2.45E-05
KRT6A	1.633449	3.71E-27
KRT76	1.666928	2.29E-09
EIF4B	-1.05124	0
ZNF740	-2.04895	4.12E-105
MFSD5	-2.22043	2.58E-76
ARL14EP	-1.07354	8.14E-23
SP1	-1.64285	5.80E-188
HOXC13	-2.12898	6.13E-11
HOXC10	-1.15135	4.11E-25
HOXC6	-1.51952	7.33E-23
HOXC9	-1.35591	1.33E-16
DNAJC14	-2.64592	2.80E-103
PYM1	-1.88903	8.51E-45
RAB5B	-1.02188	3.16E-177
SUOX	-2.34509	2.02E-49
IKZF4	-4.19507	9.03E-40
NABP2	1.130562	4.97E-75
BAZ2A	-1.23685	1.98E-208
BDNF	-3.47328	1.04E-17
ZBTB39	-3.73631	6.00E-43
NAB2	-2.23663	1.50E-54
LGR4	-3.17484	3.94E-255
INHBE	-2.517	2.06E-07
DDIT3	-1.89359	8.80E-46
9-Mar	-1.65216	2.42E-41
LRIG3	-1.61637	5.99E-35
C11H12orf66	-2.06351	4.21E-51
SRGAP1	-1.12922	2.50E-37
TBK1	-2.82807	1.48E-90
RASSF3	-1.26404	2.51E-46
LEMD3	-3.90529	4.08E-158
HELB	-2.42053	1.49E-09
DYRK2	-2.4173	1.01E-53
MDM2	-2.27776	6.76E-173
YEATS4	-1.09476	1.21E-30
FRS2	-2.28598	8.74E-97
CNOT2	-1.39567	8.84E-182
LGR5	-1.11225	8.58E-71
THAP2	-2.24264	4.17E-18
ZFC3H1	-1.44943	1.62E-132
ATXN7L3B	-3.33804	0
PHLDA1	-3.279	0
BBS10	-2.32823	7.68E-30

ZDHHC17	-1.79174	7.89E-57
E2F7	-2.15873	5.14E-122
PPP1R12A	-1.39531	9.60E-200
ALX1	-2.54777	1.04E-15
DUSP6	-4.60485	0
BTG1	-4.91555	0
NUDT4	-2.34283	2.51E-303
TMCC3	-1.77884	1.20E-15
USP44	-1.68496	4.58E-47
NTN4	-1.53104	9.83E-96
CFAP54	-1.27291	8.93E-05
NAV2	-1.12898	1.49E-46
APAF1	-2.61306	8.82E-55
UHRF1BP1L	-1.62016	1.14E-113
GAS2L3	-1.02289	1.91E-15
GNPTAB	-1.58346	3.62E-57
E2F8	-3.4051	3.04E-109
ZDHHC13	-1.13987	8.22E-34
NUAK1	-1.36491	3.18E-137
TCP11L2	-2.10753	3.06E-18
MTERF2	-1.1088	1.52E-09
FICD	-1.3578	5.86E-17
SSH1	-1.15007	1.89E-88
USP30	-1.66787	4.80E-59
ALKBH2	-1.04629	1.26E-08
UNG	1.006487	1.06E-62
KCTD10	-1.04099	2.19E-53
LOC103239100	-2.21837	1.27E-14
SPTY2D1	-2.52208	1.86E-163
SH2B3	-1.06289	5.31E-41
ATXN2	-1.42283	3.66E-78
B4GALNT4	1.359663	3.66E-107
NAA25	-1.36873	3.01E-117
TBX3	-3.5287	2.33E-96
MED13L	-1.09446	3.52E-173
LOC103239208	-2.00331	1.66E-04
FBXO21	-1.15625	2.22E-47
BICDL1	-1.49983	2.83E-221
SIRT4	-1.45091	8.09E-06
TRIAP1	-1.34772	1.27E-80
HNF1A	-1.68357	9.74E-04
KDM2B	-1.81255	2.15E-55
TMEM120B	1.070629	7.22E-23
SETD1B	-2.67554	8.24E-44

BCL7A	-1.55327	1.24E-15
RSRC2	-2.16229	2.34E-103
VPS37B	-1.70389	1.11E-105
C11H12orf65	-1.70484	6.92E-24
SNRNP35	-2.21644	2.39E-28
DNAH10	-1.06389	5.40E-04
UBC	-1.43289	0
DDX51	1.056859	9.18E-35
GALNT9	-2.09476	7.00E-05
LOC103239448	1.549091	1.38E-12
PGAM5	1.034518	6.36E-111
ZNF605	-1.21498	2.78E-21
ZNF84	-1.92844	5.52E-28
ZNF10	-1.27688	8.40E-10
MUC19	-3.03587	7.64E-04
LOC103239500	1.084773	1.96E-65
ZCCHC8	-3.00208	1.44E-118
SBNO1	-1.32266	1.77E-76
ZMYND19	-1.31598	7.13E-19
NELFB	1.023735	1.89E-153
TOR4A	-1.50536	2.83E-06
TPRN	-1.52772	1.09E-33
C12H9orf139	2.622931	1.53E-06
PAXX	1.134054	2.24E-15
C8G	2.385593	1.40E-07
AJM1	-1.2262	3.27E-04
LCN15	-3.3578	7.31E-05
NOTCH1	-2.21179	8.19E-193
SOX6	-1.44768	1.05E-60
C12H9orf116	-1.27647	1.30E-24
PPP1R26	-1.37983	9.43E-128
SURF2	1.012063	2.31E-31
RALGDS	-2.51592	5.07E-125
TSC1	-1.19156	6.58E-70
GTF3C4	-2.8521	7.02E-122
TTF1	-1.83105	3.58E-78
SETX	-1.77907	1.83E-153
NTNG2	-1.19413	1.75E-16
ABL1	-1.09025	5.13E-192
PRDM12	-4.43959	4.75E-20
PTGES	-2.45091	5.06E-04
IER5L	-6.41862	4.45E-118
DOLK	-2.1034	8.90E-39
LRRC8A	-1.48775	2.95E-23

CYP2R1	-1.46275	2.54E-34
GOLGA2	-1.1032	8.38E-110
PTGES2	1.44603	2.44E-202
FAM102A	-2.08951	2.90E-82
ZNF79	-1.33674	6.73E-08
ZBTB34	-4.4953	4.20E-06
ZBTB43	-3.22701	7.48E-34
GAPVD1	-1.02983	2.92E-69
NR5A1	4.356446	3.72E-04
ZBTB6	-3.29683	3.11E-102
ZBTB26	-2.87237	3.03E-17
PDCL	-1.58607	5.42E-20
SENP6	-1.39114	1.31E-50
PHIP	-1.76958	1.94E-132
TTK	-1.15625	2.46E-24
TENT5A	-1.79195	2.09E-10
TPBG	-2.25245	2.31E-242
RWDD2A	-1.81031	3.51E-13
PRSS35	-1.62083	5.64E-04
TBX18	-1.33674	2.60E-21
LOC103240069	-1.98953	1.15E-24
CNR1	-1.81348	4.57E-10
PNRC1	-5.27285	0
CASP8AP2	-3.21246	1.18E-52
EPHA7	-2.12226	7.28E-305
FUT9	-1.16278	3.28E-07
LOC103240143	1.680785	7.29E-04
LATS1	-1.87717	6.74E-29
LOC103240189	-2.97447	1.45E-21
SASH1	-1.76398	3.15E-25
FBXO30	-3.3951	9.12E-55
HIVEP2	-1.77568	6.92E-92
CITED2	-4.86495	0
HECA	-1.26095	5.70E-20
TNFAIP3	-1.61447	2.07E-95
PDE7B	-3.38637	3.12E-20
SGK1	-3.84323	2.58E-41
SLC2A12	-2.07852	5.70E-24
ADM	-7.4339	2.04E-38
THEMIS	3.549091	1.05E-05
RNF146	-1.4769	3.41E-42
RNF217	-2.24651	1.03E-50
WEE1	-3.97447	1.83E-116
TBC1D32	-1.2328	6.25E-13

ASF1A	-1.27194	7.98E-13
MCM9	-2.46883	6.31E-38
ZNF143	-1.13005	1.29E-25
CEP85L	-1.0238	2.52E-07
RSPH4A	-1.80141	2.97E-04
TSPYL1	-2.33005	5.09E-158
TSPYL4	-1.42421	3.22E-52
FRK	-1.05425	3.62E-16
MARCKS	-2.41223	6.37E-225
TSSC4	-1.50257	6.32E-20
MFSD4B	-1.56569	9.03E-36
REV3L	-2.29184	2.38E-109
TRAF3IP2	-1.2372	7.53E-18
CDK19	-1.25031	9.64E-72
CDC40	-1.28986	1.89E-54
ZBTB24	-3.52416	4.97E-40
SESN1	-3.40299	6.16E-86
FOXO3	-2.41877	1.87E-193
SOBP	-1.56393	4.70E-79
C13H6orf203	-1.59505	1.80E-32
CD24	-3.65907	0
CRYBG1	-1.2955	1.00E-140
PRDM1	-3.15135	3.51E-04
LIN28B	-1.34903	1.99E-04
UFL1	-1.0304	1.67E-69
ZBTB2	-2.70357	8.58E-32
SYNE1	-2.20085	1.32E-265
FBXO5	-3.35965	2.43E-97
RGS17	-1.18787	2.06E-08
SCAF8	-2.65385	2.76E-196
ARID1B	-1.12818	2.83E-66
SYNJ2	-1.02207	3.58E-65
TULP4	-1.93565	6.92E-86
RSPH3	-1.50338	9.30E-12
LMO1	-1.13541	7.01E-04
WTAP	-1.61314	1.75E-205
UNC93A	-1.63533	1.67E-06
C13H6orf120	-1.27969	1.14E-28
FAM120B	-1.60404	2.78E-89
DLL1	-3.23227	9.09E-08
LOC103240987	-3.33355	1.93E-08
TBP	-1.03891	9.32E-14
LOC103241052	-3.4953	2.24E-05
ZNF2	-4.16973	6.70E-24

ZNF514	-1.97447	2.55E-04
ITPRIPL1	-1.1654	1.08E-12
NEURL3	-1.77284	9.41E-08
ANKRD23	-1.6639	9.50E-07
SEMA4C	-2.31545	4.64E-66
CNGA3	-1.52948	1.67E-28
UNC50	-1.32585	4.18E-41
LIPT1	-1.82595	2.46E-06
REV1	-2.02649	1.67E-77
CNOT11	-1.01434	6.52E-35
TGFBRAP1	-1.04792	2.27E-49
C14H2orf49	-1.56597	4.64E-59
ST6GAL2	3.015501	7.42E-04
RANBP2	-2.17436	0
SOWAHC	-2.72037	1.80E-28
BCL2L11	-3.84323	5.20E-04
ZC3H6	-1.10965	3.10E-31
CDKN1C	-1.50329	6.74E-29
CKAP2L	-2.17347	2.35E-246
LOC103241298	-2.05696	8.63E-04
RRP8	-1.52726	1.48E-10
RPIA	1.088165	4.10E-23
EIF2AK3	-2.45011	2.60E-163
PPM1L	-1.06123	4.75E-09
TRIM59	-1.62799	3.42E-33
SMC4	-1.13151	0
LOC103241405	-1.06277	2.55E-32
SHOX2	-1.38808	2.75E-15
CCNL1	-1.85262	3.05E-103
TIPARP	-3.22068	0
C15H3orf33	1.472412	4.52E-42
PLCH1	-1.55851	2.02E-49
DHX36	-1.03648	3.15E-63
ARHGEF26	-1.39844	4.87E-08
RAP2B	-3.18725	4.05E-92
P2RY1	-5.82595	2.05E-27
MED12L	1.13393	2.39E-06
SIAH2	-1.65207	6.36E-34
TSC22D2	-3.91782	1.00E-214
TM4SF1	-1.01222	0
PLSCR4	-1.59387	2.33E-09
DIPK2A	-1.37106	1.89E-41
TRPC1	-1.23113	5.98E-45
XRN1	-1.02154	4.56E-55

RASA2	-1.0294	2.43E-24
ZBTB38	-2.3988	0
PXYLP1	-1.92271	3.73E-71
DBR1	-1.4794	2.14E-36
NCK1	-2.0651	4.24E-52
MSL2	-3.9119	1.14E-128
AMOTL2	-3.73603	0
SLCO2A1	-1.43797	2.25E-35
LOC103241658	-1.02929	3.18E-137
ASTE1	-2.4236	3.56E-15
PIK3R4	-1.93136	2.80E-167
PLCL2	-1.99064	4.40E-23
SATB1	-2.0754	4.79E-69
NR1D2	-1.80846	7.34E-124
THRB	-1.11624	2.91E-13
RARB	-1.28963	6.30E-29
OXSM	-2.16107	4.18E-93
EOMES	-2.87717	8.84E-06
TGFBR2	-2.34299	2.53E-298
LOC103241812	-2.96187	8.88E-14
ZNF860	-1.65602	1.14E-05
EIF4A2	-1.10285	1.26E-216
BCL6	-3.8682	1.44E-176
CLDN1	-1.10469	5.00E-68
GMNC	-5.30889	2.59E-10
MB21D2	-2.14074	3.11E-63
HES1	-4.0106	1.88E-80
AHNAK	-1.43659	0
FAM43A	-2.14499	6.01E-29
LOC103241951	-1.5864	7.82E-04
LOC103241983	-1.63751	7.49E-43
CEP19	-1.07709	3.69E-09
WDR53	-2.37467	1.35E-23
FBXO45	-1.24857	5.44E-17
RNF168	-2.15135	3.28E-27
UBXN7	-1.61631	1.18E-67
LOC103242077	-2.04784	5.85E-10
GEMIN4	-2.08766	1.28E-53
C16H17orf97	-3.4953	3.00E-22
SMYD4	-1.11387	5.14E-13
LOC103242100	-1.83557	4.56E-07
WDR81	-1.0996	2.51E-29
TLCD2	-1.17527	4.45E-06
SCARF1	4.270095	0

MNT	-2.2289	3.75E-44
HASPIN	-3.33083	1.17E-33
UBE2G1	-1.82535	1.16E-132
SMTNL2	-2.10435	4.33E-80
ZFP3	-2.41174	4.40E-30
ZNF594	-3.54261	4.61E-45
MIS12	-2.20245	2.55E-35
WSCD1	1.441778	1.14E-34
MED31	-2.03814	7.03E-47
SLC16A13	-1.89148	1.01E-40
PHF23	-2.95782	1.05E-180
KCTD11	-3.58724	7.92E-161
TMEM102	-1.98229	7.79E-27
ZBTB4	-1.16006	8.73E-72
SOX15	-3.77284	7.79E-04
KDM6B	-2.6896	3.08E-63
ALOXE3	1.382442	4.62E-19
PER1	-2.3578	3.70E-41
BORCS6	-3.96479	8.82E-84
KRBA2	-2.62083	3.43E-08
SOX18	-3.7177	1.68E-11
ADPRM	-1.39733	5.23E-09
MAP2K4	-1.30652	2.48E-24
HS3ST3A1	-1.53728	3.52E-62
HS3ST3B1	-2.09174	4.93E-128
LOC103242438	-2.87717	8.84E-06
CENPV	1.157002	5.49E-84
MED9	-1.60066	2.07E-41
RAI1	-2.14592	1.92E-82
ALKBH5	-1.29694	2.05E-100
GID4	-1.85092	1.28E-119
MIEF2	-2.04193	7.35E-87
SMCR8	-2.49729	6.62E-68
FAM83G	-2.11116	8.44E-28
MAPK7	-2.56985	3.37E-80
AKAP10	-2.16515	2.58E-74
USP22	-1.35824	5.17E-199
NATD1	-1.73731	4.00E-22
NCBP3	-1.01517	8.46E-19
PIMREG	1.922135	1.35E-16
ARFRP1	1.020824	5.13E-53
GMEB2	-1.0374	4.99E-26
TMEM97	-1.46348	1.23E-37
TNFAIP1	-1.04478	2.20E-44

SDF2	-1.14846	2.50E-78
FAM222B	-3.09115	1.79E-92
PHF12	-2.14542	6.55E-224
NUFIP2	-2.01651	0
CORO6	1.268282	9.83E-40
SSH2	-2.17644	6.09E-60
GOSR1	-1.14524	1.07E-55
TEFM	-1.90437	7.70E-33
ATAD5	-1.36393	5.85E-30
CDK5R1	-2.57331	4.26E-21
ZNF830	-4.99449	5.28E-102
SLFN5	-4.62083	6.20E-23
SLFN12	-2.3578	5.94E-09
PEX12	-2.51962	5.69E-65
CCL5	7.178351	3.03E-66
HEATR6	-1.22364	5.66E-114
HNF1B	-3.34345	0
SYNRG	-1.43672	8.75E-99
ACACA	-1.03507	0
LHX1	-4.23586	0
GGNBP2	-1.78733	1.48E-193
USP32	-1.10795	4.45E-136
APPBP2	-1.31607	0
PPM1D	-3.12231	0
INTS2	-1.53801	3.07E-97
MED13	-2.11638	0
RPS6KB1	-1.34789	7.32E-111
PTRH2	-2.41462	1.43E-108
VMP1	-1.12238	0
YPEL2	-3.5098	1.76E-90
SMG8	-3.86485	3.51E-240
MTMR4	-1.4641	1.10E-263
RNF43	-1.28963	1.27E-04
SRSF1	-1.03825	8.49E-193
VEZF1	-2.60428	0
YTHDF1	-3.40299	1.29E-170
COIL	-2.09133	2.86E-156
MBTD1	-1.05918	2.64E-45
TOB1	-4.88509	2.12E-213
LUC7L3	-1.28539	1.28E-215
XYLT2	-1.30889	4.05E-75
DIDO1	-2.11465	1.11E-187
SGCA	-1.08478	2.78E-06
ITGA3	1.098764	0

DLX4	-1.02953	4.28E-07
TNRC6C	-1.69414	5.10E-44
JMJD6	-1.63248	5.85E-79
SPHK1	1.35333	3.96E-35
QRICH2	2.286057	8.45E-05
FOXJ1	-1.2798	3.72E-06
TRIM47	-1.52714	1.79E-53
SLC25A19	1.481977	5.01E-21
USH1G	-1.41027	3.88E-04
NAT9	1.125045	9.78E-40
LOC103243079	-2.25826	1.38E-04
GPRC5C	-1.87257	1.65E-271
SSTR2	4.402112	1.78E-63
FAM104A	-1.31026	4.52E-60
CDC42EP4	-2.63082	1.57E-43
MAP2K6	-1.91034	2.90E-06
SLC16A6	-1.4051	4.07E-09
BPTF	-1.71928	4.77E-260
HELZ	-1.32301	1.78E-95
AXIN2	-1.17337	2.49E-04
GNA13	-2.76927	0
TANC2	-1.05123	5.85E-76
TLK2	-1.59676	4.80E-129
WNT9B	-2.66036	1.66E-08
KANSL1	-3.02582	0
PLEKHM1	-1.15902	1.01E-42
MAP3K14	-1.64845	1.55E-193
HEXIM2	-1.74159	3.82E-17
HEXIM1	-2.3258	0
KIF18B	-1.38215	6.45E-31
GFAP	-1.17358	0
PPP1R3D	-4.27746	3.18E-34
LOC103243306	-2.70725	1.66E-18
CCDC103	-1.62083	2.14E-15
FAM217B	-2.69005	4.00E-50
GJC1	-1.77284	4.34E-57
FZD2	-3.73762	0
ITGA2B	1.848652	4.25E-04
C16H17orf53	-1.56768	1.79E-38
ETV4	1.733195	3.93E-70
ARL4D	-4.15135	1.18E-08
PSME3	1.141686	0
PLEKHH3	-1.52215	2.58E-33
RETREG3	-2.45818	3.74E-198

PSMC3IP	1.562086	1.44E-91
KAT2A	1.258326	5.43E-252
KLHL11	-1.48333	2.55E-05
KRT14	1.790595	3.89E-17
KRT16	1.27575	2.65E-07
KRT19	-1.17705	4.56E-24
KRT13	1.348044	1.01E-13
LOC103243474	1.863261	9.78E-11
LOC103243485	-5.06562	8.03E-09
KRT28	1.610492	8.20E-05
RARA	-1.60741	1.21E-67
MSL1	-1.06225	1.42E-91
NR1D1	-2.01908	1.75E-31
GRB7	-2.3887	2.20E-157
CDK12	-1.25981	6.31E-168
STAC2	-1.31236	3.84E-25
CWC25	-1.96748	1.12E-71
PCGF2	-1.04654	9.90E-81
EPOP	-1.1241	2.23E-15
ARHGAP23	-1.22191	5.51E-115
TBKBP1	-1.28729	2.34E-66
SP2	-1.88149	2.09E-71
HOXB6	-1.12737	3.62E-28
HOXB5	-2.44047	3.72E-38
HOXB4	-1.21958	1.76E-07
HOXB3	-1.1434	2.21E-12
HOXB7	-1.40448	6.88E-26
HOXB8	-2.25444	6.36E-32
IGF2BP1	-1.71394	7.26E-04
ZNF652	-1.82014	1.25E-14
RAB22A	-1.07145	5.64E-40
SOCS3	-6.91034	2.25E-215
CANT1	-1.78421	0
CBX2	-1.23518	1.11E-07
CBX8	-3.8872	1.12E-60
CBX4	-4.01256	3.96E-173
PMEPA1	-1.5261	0
PCK1	-2.93817	2.24E-18
BAHCC1	-1.35248	5.68E-50
FSCN2	-2.02076	5.99E-08
MRPL12	1.093514	1.14E-223
PYCR1	1.128069	5.10E-197
CENPX	1.514534	1.13E-115
LRRC45	1.166409	8.21E-36

DUS1L	1.306766	2.17E-141
TEX19	-2.60471	8.58E-10
BMP7	2.262001	8.58E-43
METRNL	-2.42819	2.28E-68
TFAP2C	-2.91034	6.84E-18
TAOK1	-2.04827	0
LOC103243832	-3.00119	9.53E-04
SMURF2	-1.01173	1.34E-35
WIPF2	-1.58533	5.39E-44
MED1	-1.09555	7.17E-133
OXLD1	-2.02245	8.12E-09
CASS4	-2.65254	2.92E-10
AURKA	-1.3546	1.65E-118
CSTF1	-2.54442	9.59E-91
OGFRL1	-1.44099	2.48E-52
MC3R	-4.36977	5.01E-19
PHF3	-2.37068	1.22E-292
PTP4A1	-1.22797	0
CYP24A1	-1.58841	6.25E-08
ZNF451	-1.30744	1.35E-29
KIAA1586	-3.0055	5.71E-17
LOC103243978	-1.09476	3.26E-06
DST	-1.21886	0
FAM83B	-2.38951	3.45E-05
PARD6G	1.307483	7.87E-31
ADNP2	-3.89753	4.92E-217
PQLC1	1.061456	5.11E-31
NFATC1	-1.59834	3.53E-37
ZNF217	-4.24053	5.68E-305
ZNF407	-1.62083	5.21E-30
TIMM21	-2.61488	2.44E-141
SOCS6	-3.43823	1.09E-100
DSEL	-2.34953	7.37E-46
BCL2	-2.14752	0
PHLPP1	-1.65983	7.30E-111
ZCCHC2	-2.34538	5.76E-90
RELCH	-1.27248	9.68E-72
ZFP64	-1.68795	6.69E-27
MALT1	-2.62228	0
ALPK2	-1.77566	0
NFATC2	-1.80846	3.32E-06
ONECUT2	-1.8569	2.59E-11
LOC103244249	-1.06234	3.72E-04
RGMB	-2.97447	1.76E-16

CHD1	-2.33656	8.86E-236
MOCS3	-2.24886	4.44E-26
GIN1	-2.46089	1.98E-27
NUDT12	-1.10589	9.33E-47
EFNA5	-1.0991	4.40E-18
ADNP	-3.09179	0
STARD4	-1.59004	1.60E-55
EPB41L4A	-1.41018	4.09E-80
APC	-2.42087	2.24E-127
PARD6B	-4.83288	5.49E-65
LOC103244341	-1.47476	9.11E-07
FEM1C	-5.05906	3.62E-137
TICAM2	-1.54488	5.96E-23
SEMA6A	-2.60357	0
TNFAIP8	-2.72435	2.66E-116
LOX	-1.27782	7.49E-83
PTPN1	-1.08522	3.10E-85
ZNF608	-4.05011	1.58E-144
CEBPB	-5.46822	0
GRAMD2B	-1.76931	1.66E-25
PHAX	-1.13216	1.10E-39
MEGF10	-1.0089	3.64E-05
TMEM189	-1.58019	1.21E-43
PRRC1	-1.33055	2.21E-99
CDC42SE2	-1.08968	7.32E-38
FNIP1	-2.05473	1.65E-101
RAPGEF6	-1.6639	4.58E-40
IRF1	-2.21807	1.41E-43
SPATA2	-2.50499	2.80E-56
GDF9	-1.42476	2.02E-60
AFF4	-1.5071	0
ZCCHC10	-1.22852	3.79E-16
FSTL4	-1.41174	7.59E-06
CDKL3	-1.14021	3.99E-05
JADE2	-1.48017	6.60E-47
B4GALT5	-1.65378	1.39E-139
LOC103244555	-1.60778	1.59E-69
LOC103244556	-1.83219	5.21E-66
CAMLG	-1.26328	1.96E-63
PCBD2	1.192606	5.87E-14
C23H5orf66	-1.80846	9.70E-09
SLC25A48	1.605675	4.17E-05
HNRNPA0	-1.1385	8.83E-97
EGR1	-4.51586	1.13E-190

KDM3B	-1.0328	3.45E-111
FAM53C	-1.30111	1.76E-60
BRD8	-1.54278	8.31E-153
FAM13B	-1.47853	4.17E-34
PREX1	1.050508	2.18E-40
CXXC5	-1.57233	6.24E-306
IGIP	-2.4868	1.67E-101
PURA	-1.12446	2.30E-20
TAF7	-1.86954	0
LOC103244694	-3.53837	1.50E-05
NR3C1	-2.14388	2.18E-148
KCTD16	-2.32538	6.94E-05
ZMYND8	-2.17566	4.04E-178
RBM27	-1.99909	4.89E-158
JAKMIP2	-1.11157	6.82E-38
FBXO38	-1.97142	2.75E-163
ADRB2	-4.0938	1.33E-213
GRPEL2	-1.24738	7.53E-06
PCYOX1L	-2.19148	9.31E-33
PPARGC1B	-3.96818	7.85E-288
PDE6A	5.838926	1.07E-05
SLC26A2	-1.04964	2.56E-46
TIGD6	-4.38601	2.92E-19
SYNPO	-1.03688	6.70E-39
RBM22	-1.49805	4.79E-81
DCTN4	-1.00275	6.56E-67
SLC36A2	2.964242	5.50E-15
MFAP3	-1.73142	5.08E-90
SAP30L	-1.11685	5.20E-30
CNOT8	-1.99752	2.95E-133
HAVCR1	-1.50136	0
MED7	-3.1532	3.56E-92
TP53RK	-2.44661	2.78E-31
RNF145	-2.98853	0
PWWP2A	-1.72271	1.83E-46
CCNJL	-1.13896	1.16E-13
ZBED8	-2.93741	1.42E-30
ZNF334	-2.26294	1.63E-26
PANK3	-1.39666	3.36E-69
FOXI1	5.134054	7.11E-04
LOC103244958	-1.86595	2.94E-05
TLX3	-1.77284	4.01E-04
FGF18	-2.64731	1.39E-06
UBTD2	-1.34799	1.11E-56

NEURL1B	-1.52891	7.40E-27
DUSP1	-3.58146	3.69E-296
CPEB4	-1.36297	2.00E-80
SLC35C2	1.200232	8.95E-119
MSX2	-5.91034	5.65E-29
FAF2	-1.02559	1.35E-141
HK3	2.786131	3.20E-11
UIMC1	-1.35038	7.80E-44
NSD1	-1.14689	1.48E-245
RAB24	1.060156	2.83E-57
SLC34A1	-2.33355	3.37E-06
LOC103245076	1.093662	1.70E-31
LOC103245077	1.159956	0
CLK4	-2.70854	5.12E-76
NCOA5	-3.00013	3.60E-165
ZNF354A	-1.71705	2.58E-44
ZNF879	-3.45091	1.95E-25
MAML1	-1.21479	1.02E-89
LOC103245129	-1.82014	5.40E-05
ZFP62	-1.9392	9.09E-76
LOC103245175	-3.2058	2.37E-04
ZSWIM1	-2.96014	3.11E-56
ZFP2	-1.27403	1.61E-04
ABHD17C	-1.45977	7.89E-104
MORF4L1	-1.27832	6.89E-244
IREB2	-2.90308	0
ZSWIM3	-2.96748	6.14E-24
PEAK1	-1.27369	2.91E-25
ISL2	-1.11387	1.14E-05
SIN3A	-2.67774	0
IMP3	-2.23518	3.91E-51
SNX33	-2.39342	5.96E-55
C26H15orf39	-2.79432	2.66E-22
WFDC8	3.905547	3.57E-07
SEMA7A	-2.17779	0
PML	-2.13605	0
MYO9A	-1.96381	1.06E-143
SENP8	-1.57192	5.94E-10
LARP6	-1.54402	2.99E-26
KIF23	-1.34087	7.80E-95
GLCE	-1.25685	6.95E-74
SPESP1	-1.87445	9.90E-27
CORO2B	1.08776	6.65E-30
FEM1B	-3.84393	1.02E-281

PIAS1	-2.06646	2.70E-126
C26H15orf61	-2.34861	5.24E-28
SMAD6	-1.69037	1.08E-10
MEGF11	1.286057	3.60E-13
DENND4A	-1.70461	8.30E-121
SLC24A1	-1.56053	1.62E-17
IGDCC3	-1.11016	4.76E-24
CLPX	-1.06294	2.43E-45
RASL12	-3.84323	6.26E-07
PLEKHO2	-2.97753	1.66E-105
STK4	-1.07502	1.81E-50
RORA	-1.19238	1.10E-04
RIMS4	1.215668	3.73E-06
BNIP2	-1.33988	1.44E-48
RNF111	-1.66427	1.17E-116
SLTM	-1.01393	1.13E-83
ADAM10	-1.39076	0
POLR2M	-1.30596	1.47E-38
CGNL1	-1.65711	6.07E-97
RFX7	-1.6528	5.85E-183
C26H15orf65	-1.2394	8.49E-04
FAM214A	-1.12405	5.69E-24
LEO1	-1.83512	1.40E-67
USP8	-1.21955	3.78E-115
GABPB1	-1.18718	6.84E-24
FGF7	2.034518	4.44E-04
SECISBP2L	-1.6573	1.02E-191
CEP152	-1.26926	1.39E-15
OSER1	-2.57339	1.08E-131
BLOC1S6	3.784814	0
MFAP1	-1.96859	2.60E-160
LCMT2	-2.96748	6.14E-24
ZSCAN29	-2.18026	1.11E-89
TTBK2	-1.45898	1.53E-57
FADD	-1.84048	1.18E-17
STARD9	-1.18115	5.52E-29
SPTBN5	1.26037	8.50E-19
LTK	1.116776	2.05E-08
MGA	-2.04932	1.10E-156
INO80	-1.57727	8.22E-113
CHAC1	-3.73631	1.54E-32
VPS18	-1.89527	2.24E-100
RAD51	1.067909	1.33E-32
C26H15orf62	-3.53837	5.74E-10

RPUSD2	-3.53572	7.00E-71
CCDC32	-2.18643	2.39E-27
BAHD1	-1.42439	1.42E-52
DISP2	-1.27453	1.53E-17
ANKRD63	-3.91034	3.47E-04
PAK6	-1.3961	1.06E-19
BMF	-4.64454	0
THBS1	-1.36704	0
SPRED1	-1.28157	4.11E-53
ZNF770	-1.1647	6.07E-58
PGBD4	-4.4051	2.25E-10
FMN1	-2.24778	0
GREM1	-1.19355	0
ARHGAP11A	-1.3783	2.06E-189
MTMR10	-1.74973	1.96E-297
NSMCE3	-1.65212	5.89E-74
TJP1	-1.10429	1.32E-143
MTG1	1.029931	9.91E-73
TUBGCP5	-1.63785	4.30E-51
GABRA5	-1.40236	2.27E-12
LOC103246028	-1.97969	1.33E-04
KNL1	-1.23438	4.58E-22
FRYL	-1.00943	2.03E-98
FAM83D	-4.03372	1.82E-95
TEC	-2.13541	8.47E-22
NFXL1	-1.18037	5.23E-24
GABRA2	-1.64457	0
DCAF4L1	-1.37902	7.50E-07
RBM47	-1.10621	8.73E-89
TLR1	-2.23122	1.34E-05
KLF3	-3.57866	0
RELL1	-2.13273	6.05E-13
C27H4orf19	5.212436	1.30E-18
PCDH7	-1.05972	1.77E-04
RBPJ	-1.06214	3.01E-66
ZCCHC4	-1.24886	4.53E-12
KIAA1755	-1.24532	4.39E-05
SLIT2	-1.32872	1.84E-40
DCAF16	-1.84142	3.90E-51
LCORL	-1.31441	2.62E-16
RPRD1B	-1.0328	6.26E-38
FBXL5	-1.94742	1.04E-168
CPEB2	-2.32538	4.06E-16
BLCAP	-1.29418	5.85E-56

BOD1L1	-1.51446	6.39E-119
NKX3-2	-1.25826	1.93E-05
HS3ST1	-4.20198	0
ZNF518B	-3.12847	2.41E-66
ZBTB49	-2.4953	1.94E-22
MSX1	-1.31271	1.90E-08
STK32B	1.317766	9.99E-04
C27H4orf50	-1.97586	6.75E-37
WFS1	-1.15111	4.41E-282
MRFAP1L1	-3.06407	1.08E-106
BLOC1S4	-2.0651	4.24E-52
KIAA0232	-2.72703	5.17E-189
TBC1D14	-1.26765	4.48E-163
TADA2B	-2.64845	7.98E-84
SH3TC1	-1.01559	7.54E-41
TRMT44	-1.88514	1.32E-30
RBL1	-1.18787	9.37E-04
MFSD10	1.057337	9.26E-53
FAM193A	-1.21598	1.98E-43
MXD4	-1.23707	5.57E-93
HAUS3	-2.78589	3.37E-24
LOC103246471	1.223851	5.86E-10
FAM53A	1.129792	3.56E-16
CCND1	1.078373	3.88E-235
JAG1	-1.55887	4.80E-17
IDUA	1.094768	3.62E-55
ZNF721	-1.00111	1.48E-20
LOC103246525	-1.92619	1.11E-25
VKORC1L1	-1.14512	2.05E-71
KCTD7	-1.69824	1.06E-68
LOC103246604	-4.2058	5.32E-09
AUTS2	-2.97582	2.32E-237
CASTOR2	-1.09789	1.52E-29
LOC103246613	-2.25826	3.70E-49
CLDN4	-1.42516	0
STX1A	1.426194	5.99E-94
DNAJC30	-2.03219	6.60E-85
VPS37D	-2.93204	7.82E-16
POM121C	-1.21549	5.12E-134
YWHAG	-1.2376	0
LOC103246669	2.785108	7.76E-09
ALKBH4	-2.71457	1.38E-47
SH2B2	-1.41723	4.33E-23
TRIM56	-1.36132	1.88E-67

UFSP1	-2.46586	1.40E-27
POP7	-1.80803	6.45E-52
BMP2	-3.5262	1.76E-31
TSC22D4	-1.07408	1.18E-41
MEPCE	-1.95166	4.43E-224
ZSCAN21	-1.62785	1.50E-55
ZNF3	-3.5879	3.10E-147
GJC3	-1.74369	5.40E-04
ZSCAN25	-2.24207	4.81E-37
CYP3A7	1.88771	8.90E-04
ZKSCAN5	-1.38406	1.88E-41
FAM200A	-2.63172	4.75E-40
ZNF394	-3.6432	5.62E-78
TMEM130	1.068466	1.24E-04
LMTK2	-1.1828	8.43E-118
USP42	-3.52449	1.89E-186
ZNF12	-3.30291	8.14E-89
FBXL18	-1.39627	3.18E-13
TNRC18	-1.81816	2.44E-150
RBAK	-1.31155	7.96E-74
FOXK1	-1.79928	1.05E-124
LFNG	-1.44248	4.62E-99
CHST12	-1.17588	1.24E-34
MRM2	-1.23465	7.81E-33
TMEM184A	-1.59775	2.45E-04
GPR146	-2.0089	1.12E-08
ADAP1	1.288856	5.79E-20
GTF2IRD1	-1.48728	2.07E-125
TMEM230	4.456795	0
ZFY	-1.71902	1.14E-07
DDX3Y	-1.91034	7.84E-13
NANP	-2.24532	3.12E-19
LOC103247079	4.118465	5.02E-100
ZNF419	-3.62083	6.78E-06
NOA1	2.52084	0
LOC103247132	-2.2676	5.97E-14
HIST2H2BE	-1.97447	6.66E-14
BOLA1	-3.0202	5.94E-73
PLEKHO1	-1.13746	3.00E-53
FASTKD5	-3.26648	8.00E-86
CIART	-2.84323	1.75E-34
MCL1	-3.18731	0
GOLPH3L	-1.86662	2.26E-92
C20H1orf56	-1.125	1.03E-07

RPRD2	-2.83904	1.09E-254
BCL9	-1.74034	9.97E-126
CDC42SE1	-1.29153	2.45E-14
LOC103247288	-1.09014	5.31E-19
ZCCHC12	-1.10456	7.00E-11
NKRF	-3.61536	3.95E-104
RNF113A	-3.62083	4.42E-20
ZBTB33	-2.29548	3.38E-50
XIAP	-1.25826	8.88E-14
ATRN	-1.04465	8.45E-124
WDR44	-1.62291	1.16E-37
C2H20orf27	1.216136	8.26E-79
LOC103247451	-3.84323	5.20E-04
PRRG1	-1.53735	1.92E-35
ADAM33	1.057238	9.17E-37
CENPB	-1.53672	6.10E-86
LOC103247559	-3.69385	2.14E-20
PDPK1	-1.02732	5.36E-23
ZCCHC3	-4.13429	5.21E-133
LOC103247767	-3.01859	7.71E-20
SOX12	-1.83634	1.88E-105
LOC103247796	-2.30057	1.12E-15
LOC103247810	-2.58019	1.71E-04
LOC103247823	-2.45091	7.42E-10
LOC103247851	-2.61353	2.80E-20
TRIM6	-1.12052	1.21E-33
TRIM68	-1.57108	6.05E-27
TRIM21	-2.99612	7.10E-61
RHOG	-1.90222	4.48E-117
ID1	-3.01859	2.13E-38
RELT	1.080705	3.32E-07
DNAJB13	-1.3578	5.04E-06
LOC103248030	-2.89452	5.65E-34
LIPT2	-4.0235	5.61E-28
POLD3	-1.36845	1.69E-34
RNF169	-3.06177	1.35E-128
LOC103248048	-3.84323	5.20E-04
LOC103248058	-1.05915	1.24E-36
EMSY	-2.2058	1.27E-69
LRRC32	-3.01261	1.83E-296
LOC103248084	-1.63224	6.19E-08
TSKU	-2.8844	3.70E-290
B3GNT6	4.051592	3.13E-08
RSF1	-2.42161	1.67E-238

THRSP	-2.69192	2.15E-63
KCTD21	-2.47143	7.95E-72
RAB30	-3.70964	6.76E-37
PCF11	-4.31557	0
DLG2	-1.02738	1.84E-107
CREBZF	-1.44673	1.52E-77
LOC103248163	-2.85841	1.60E-21
PLAGL2	-2.08413	7.83E-91
EED	-1.0784	3.11E-28
FZD4	-1.95462	3.40E-28
LOC103248202	-1.8603	4.84E-12
ASXL1	-2.42826	0
NOL4L	-1.83772	6.50E-34
MED17	-2.014	2.94E-61
ANKRD49	-3.83173	3.09E-52
FUT4	-1.45091	5.37E-04
KDM4D	-1.66814	2.15E-11
LOC103248271	-1.62357	1.33E-56
SESN3	-1.41284	3.16E-09
MAML2	-2.1851	8.52E-15
JRKL	-2.92014	3.13E-49
DNMT3B	-1.2364	9.76E-38
BIRC3	-2.4986	0
BIRC2	-1.23868	3.25E-121
DYNC2H1	-1.14434	8.07E-52
MSANTD4	-2.6733	9.80E-79
KBTBD3	-1.46826	1.27E-12
RAB39A	-2.1455	4.39E-20
NPAT	-2.42582	1.56E-122
EXPH5	-3.74414	2.54E-174
LOC103248413	5.146027	6.72E-04
ZC3H12C	-1.88049	3.09E-78
BTG4	-1.25826	5.96E-06
TIMM8B	-1.14373	2.67E-30
NNMT	-3.42819	4.06E-09
RBM7	-1.87131	1.75E-150
BUD13	-2.61099	1.23E-85
PAFAH1B2	-1.44328	9.97E-143
SCN2B	5.470718	4.34E-36
KMT2A	-2.61232	0
PHLDB1	-1.23355	0
E2F1	1.711171	4.22E-149
DDX6	-2.05058	8.13E-165
BCL9L	-2.91467	0

TESMIN	-1.94567	1.36E-09
HMBS	1.080137	3.48E-63
C2CD2L	1.111906	5.21E-36
HINFP	-1.47251	1.07E-22
CBL	-1.196	1.94E-42
RNF26	-2.09994	9.32E-303
TMEM136	-1.00376	5.80E-09
GRIK4	1.911662	1.24E-04
TBCEL	-1.34021	4.05E-14
SC5D	-1.46178	7.26E-241
HSPA8	1.113784	0
GRAMD1B	-1.56297	3.51E-29
ZNF202	-2.58556	8.79E-40
ROBO3	1.143835	6.43E-18
DDX25	-3.97447	2.32E-04
HYLS1	-1.92221	1.51E-25
PUS3	-2.99317	9.11E-79
TP53INP2	-1.71313	1.48E-61
KIRREL3	1.43806	2.26E-04
NCOA6	-1.54936	3.36E-85
KCNJ1	-2.64731	5.15E-12
ARHGAP32	-1.45091	1.17E-49
NFRKB	-1.30545	4.74E-33
LOC103248832	-1.33843	4.91E-07
ZBTB44	-1.12032	3.91E-10
ADAMTS15	-2.4584	1.18E-18
ACSS2	-1.02533	8.36E-198
LATS2	-1.40304	3.36E-75
IL17D	1.140738	3.74E-05
GJB2	-1.84323	8.46E-08
ZMYM5	-1.53162	5.69E-27
RTEL1	1.00002	3.07E-53
SACS	-1.4491	1.23E-107
TNFRSF19	-2.17509	0
TAF4	-2.12607	3.29E-31
SHISA2	-1.1758	5.34E-05
ZNFX1	-1.9027	1.16E-72
RASL11A	-1.15662	3.97E-14
MTIF3	-1.0048	1.00E-20
LNX2	-1.30529	2.22E-54
PAN3	-1.16937	2.09E-65
